# Taming Neutral Silylidenebismuthanes (Si=Bi) with a Low-Coordinate Bismuth Atom

**DOI:** 10.1021/jacs.6c10667

**Published:** 2026-06-25

**Authors:** Annapurna Saxena, Shenglai Yao, Jan Dirk Epping, Matthias Driess

**Affiliations:** Metalorganics and Inorganic Materials, Department of Chemistry, 26524 Technical University of Berlin, Straße des 17. Juni 135, Secr. C2, 10623 Berlin, Germany

## Abstract

Unsaturated main-group compounds containing heteroleptic double bonds of bismuth remain exceedingly rare due to inherent p_π_–p_π_ bond weakness. Here we report the synthesis and characterization of the first isolable silylidenebismuthane complexes, L­(Me_3_Si)­Si=Bi­(SiMe_3_) and L­(Me_3_Si)­Si=Bi­(Si^*i*^Pr_3_) ((*E*)-**1a**,**b**) (L = PhC­(N^*t*^Bu)_2_), containing a neutral but strongly polarized silicon–bismuth double bond. The complexes were obtained as the *E* isomers in 55% and 60% yield through the salt metathesis reaction of LSi^II^Cl with the corresponding potassium bis­(silyl)­bismuthanide complexes. Remarkably, LSi^II^–Bi­(SiMe_3_)_2_ and LSi^II^–Bi­(SiMe_3_)­(Si^*i*^Pr_3_) could not be observed but undergo a trimethylsilyl group migration from the Bi^III^ atom to the Si^II^ atom to form a Si=Bi moiety containing σ^3^λ^4^-coordinate Si^II^ and σ^2^λ^2^-coordinate Bi^I^ atoms. The reactions of (*E*)-**1a**,**b** with [W­(CO)_5_(thf)] cause the (*E*) → (*Z*) isomerization with respect to the silyl groups attached to furnish the corresponding terminal Bi→W­(CO)_5_ complexes (*Z*)-**2a,b**, in which the Si=Bi π bonding interaction is significantly weakened. This and the nature of the Si=Bi bonds are corroborated by DFT calculations.

## Graphical abstract

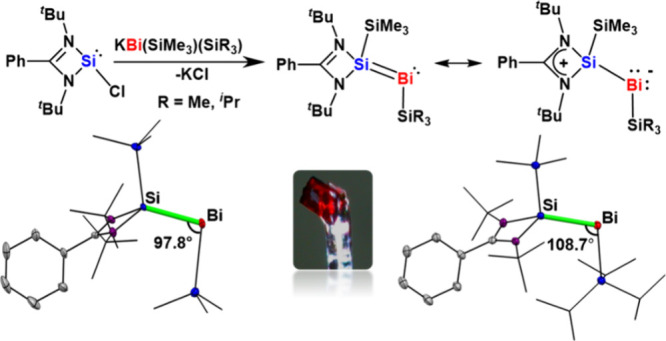

Isolation of multiply bonded heavy main-group compounds in low oxidation states has represented a formidable challenge in synthetic chemistry.^1,2^ Since West and co-workers’ landmark report of the first room-temperature stable disilene (>Si=Si<) in 1981,^3^ significant progress has been achieved in isolation of olefin analogs.^4–7^ Isolation of heteroleptic compounds of group 14 and 15 double bonds is more challenging due to an innate π-bond polarity leading to dimerization and oligomerization products.^8,9^ The seminal synthesis of the first silylidenephosphane derivative (>Si=P−) by Bickelhaupt and co-workers in 1984 ^10^ spurred extensive investigation of structure–reactivity relationships of Si=P bonds. Bulky substituents on low-valent silicon and phosphorus atoms have been instrumental for kinetic stabilization of the Si=P bond in **I** (Chart 1a).^11–16^ Similarly, isolable heavier Si=P analogues **I** (Si=As,^17–20^ Si=Sb^21,22^ ) are scarce (Chart 1a). Bismuth, the heaviest pnictogen, is of interest due to its accessible oxidation states and applications in catalysis and material science.^23–25^ However, its large atomic radius and relativistic effects create a large s–p energy gap. This results in pure 6p bonding, a fragile σ- framework, and poor p_π_–p_π_ overlap, making the isolation of multiple-bonded bismuth species a formidable challenge. Despite these hurdles, the carbene–bismuthinidene **II** (Chart 1a) was reported, which represents a rare C=Bi bond system, stabilized by ligand’s synergistic σ-donor and π-acceptor properties.^26^ Recently, the cationic gallyl–heterovinyl species **III** was synthesized as further instance of a formal C=Bi double bond.^27^ The recent report by Zhao and co-workers on isolable bis­(silylene)-stabilized bismuth­(I) cation **IV** ^28^ and our realization of redox-noninnocent bis­(silylenyl)­carborane-stabilized cationic Bi^I^ species **V** ^29^ have also advanced the coordination chemistry of low-valent bismuth atoms. Despite these milestones, neutral complexes featuring a formal silicon–bismuth double bond or Si^II^-stabilized Bi^I^ remain elusive. DFT calculations have predicted their potential accessibility^30,31^ yet no example has been realized, likely due to the paucity of suitable precursors. Beyond direct salt metathesis strategies, an attractive alternative involves the conversion of silylene-type bis­(trimethylsilyl)­pnictane species (−Si^II^–E^15^(SiMe_3_)_2_) into their doubly bonded counterparts (−Si­(SiMe_3_)=E^15^(SiMe_3_)). DFT calculations show for heavier pnictogens that the rearrangement becomes thermodynamically favorable with lower activation barriers for SiMe_3_ migration (Figure S46). Building on this strategy and following our earlier realization of the Si=P double bond,^32,33^ we now report the synthesis, isolation, and structural characterization of the first silylidenebismuthanes LSi­(SiMe_3_)=Bi­(SiMe_3_) ((*E*)-**1a**) and LSi­(SiMe_3_)=Bi­(Si^*i*^Pr_3_) ((*E*)-**1b**) (L = PhC­(N^*t*^Bu)_2_) (Chart 1c), featuring a polarized Si=Bi bond.

**Chart 1:**
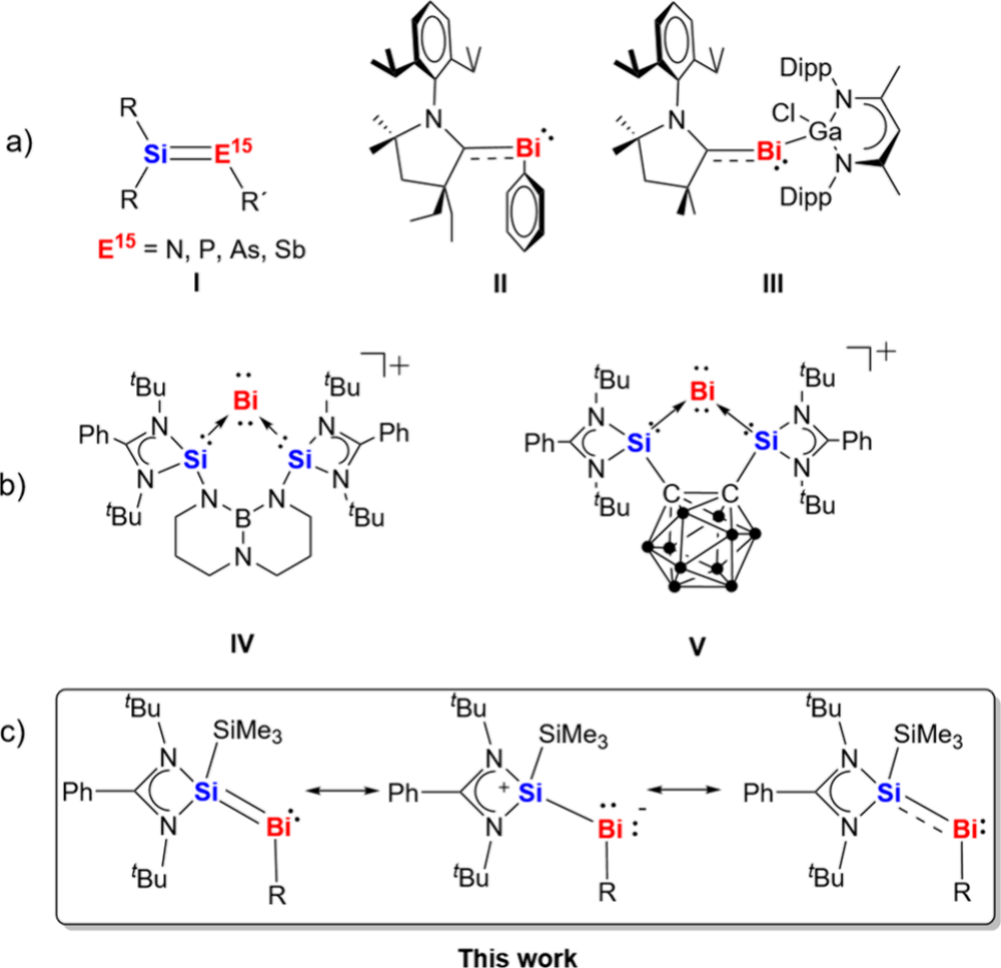
(a) Isolable Si=E^15^ Compounds **I** (R = Organic, Silyl; R′ = H, Organic, Silyl) and Bi=C Complexes **II** and **III** (Dipp = 2,6-Diisopropylphenyl); (b) Previous Reports on Bis­(silylene)-Stabilized Bismuth­(I) Cations **IV** and **V**; (c) First Isolable Neutral Silylidenebismuthane (Si=Bi) (This Work)

Complex (*E*)-**1a** was synthesized by reaction of the N-heterocyclic chlorosilylene (LSi^II^Cl) with potassium bis­(trimethylsilyl)­bismuthanide [K­(thf)_*x*_Bi­(SiMe_3_)_2_][Bibr ref34] in diethyl ether at −70 °C (Scheme 1). Unlike LSi=P­(SiMe_3_)_2_, which can be isolated and thermally converted to the Si=P species only upon heating to 100 °C,^32^ the putative silylene-type Si–Bi compound **1a′** could not be detected, even at low temperature. Instead, a rapid intramolecular migration of a silyl group from bismuth to silicon occurs. Dark-red crystals of (*E*)-**1a** were isolated in 55% yield using diethyl ether at low temperature. The use of toluene, tetrahydrofuran, or ambient temperature results in decomposition to elemental bismuth.

**Scheme 1:**
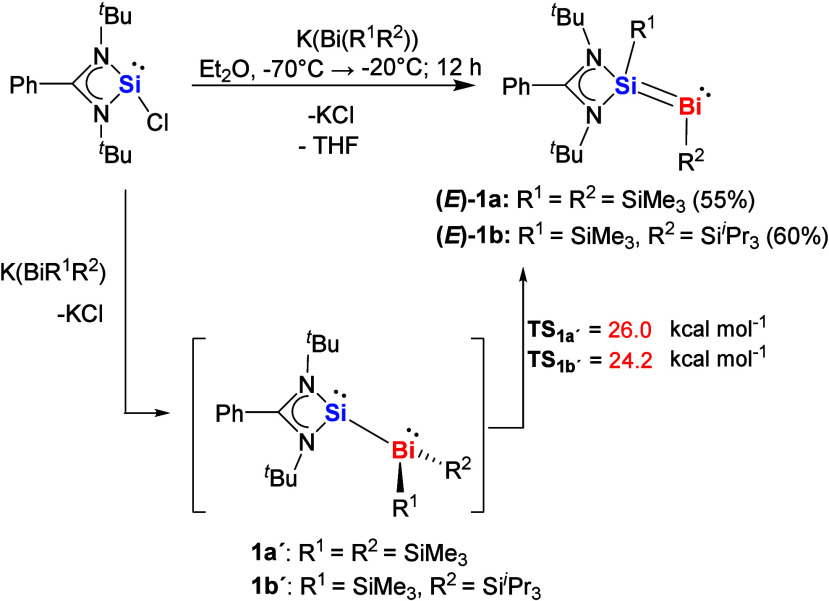
Synthesis of Silylidenbismuthanes (*E*)-**1a**,**b** via **1a′** and **1b′** ^a^

a The relative Gibbs free energies are −9.5 kcal mol^–1^ for **1a′** to (*E*)-**1a** and −12.3 kcal mol^–1^ for **1b′** to (*E*)-**1b**. The reaction energy barriers are 26.0 and 24.2 kcal mol^–1^, respectively (see details in Figure S46).

The molecular structure of (*E*)-**1a** was confirmed by single-crystal X-ray diffraction (scXRD) analysis (Figure 1a), NMR spectroscopy, and mass spectrometry. The compound crystallizes in the monoclinic space group *P*2_1_/*n*. The Si1 atom adopts a tetrahedral geometry reminiscent of the Si=E^15^ (E^15^ = P, N) analogs^35,36^ and related Si=E^16^ (E^16^ = O, S) species.^37,38^ The Si1–Bi1 distance in (*E*)-**1a** (2.536(1) Å) is notably shorter relative to the Si3–Bi1 single bond (2.637(1) Å), indicating a higher Si1–Bi1 bond order, which is corroborated by DFT calculations (Figure 3). The Si=Bi distance is slightly shorter than that reported for Zhao’s bis­(silylene)-stabilized bismuth­(I) cation **IV** ^28^ and our previously described bis­(silylenyl)­carborane-stabilized Bi^I^ complex **V**.^29^ However, the latter systems rely on an overall monocationic charge that facilitates Si→Bi σ- donation and stabilizes the two-coordinate bismuth­(I) center. (*E*)-**1a** represents the first neutral framework with a Si=Bi interaction. The Si1–Bi1–Si3 bond angle of 97.83(3)° is considerably smaller than the Si–P–Si angle of its Si=P analog (106.6(1)°).^32^ Accordingly, the calculated Si–E^15^–Si angles (E^15^ = P, As, Sb, Bi) decrease down the Group 15 element series (Figure S44), reflecting the typical reluctance of heavy main-group elements to undergo s–p hybridization.^39^ In addition, the p_π_–p_π_ orbital interactions become weaker and the Si=E^15^ distances longer due to increasing Pauli repulsion between the filled core orbitals.^40^

**Figure 1:**
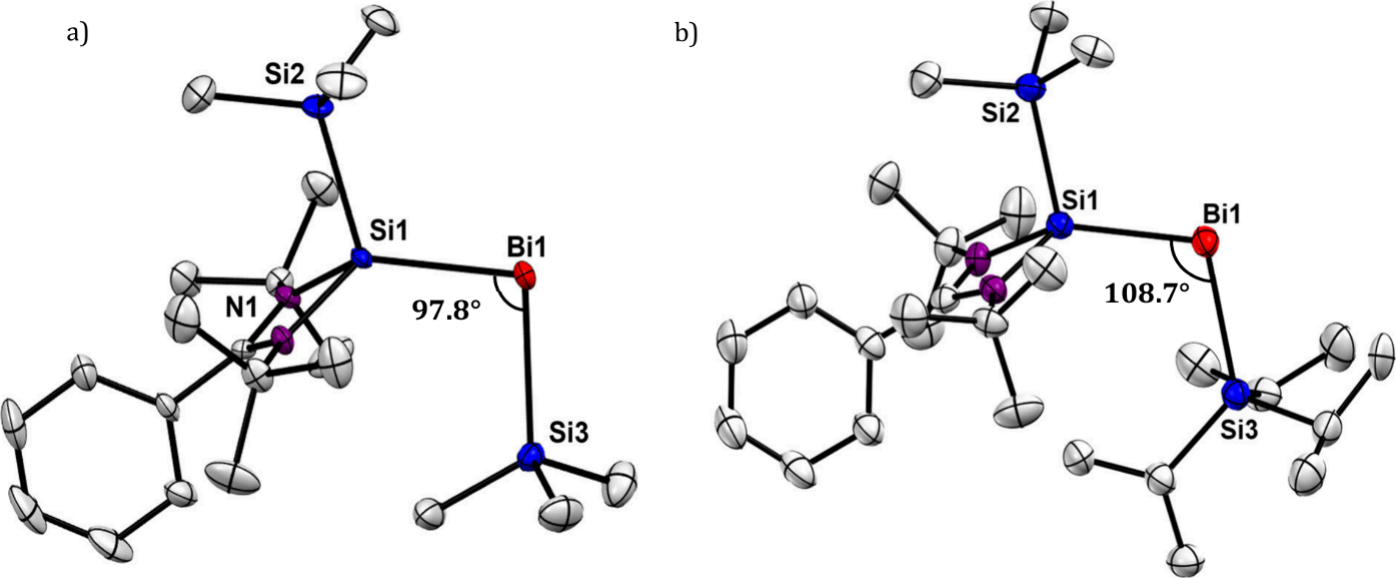
Molecular structures of (*E*)-**1a** and (*E*)-**1b** (50% probability ellipsoids; H atoms omitted for clarity).

The ^1^H NMR spectrum of (*E*)-**1a** in C_6_D_6_ shows that the Si-bound silyl group (δ = 0.3 ppm) is more shielded than the Bi-bound silyl group (δ = 1.2 ppm). The ambient-temperature ^29^Si­{^1^H} NMR spectrum reveals only two resonances at δ = −28.4 (Me_3_ *Si*–Bi, sharp) and 40 ppm (*Si*=Bi, broad), respectively. However, the ^29^Si­{^1^H} NMR spectrum recorded at −70 °C in C_7_D_8_ resolves three signals: two sharp resonances at δ = −28.4 (Me_3_ *Si*–Bi) and −7.6 ppm (Me_3_ *Si*–Si=Bi) and a broad resonance at 37.5 ppm (*Si*=Bi). Consistently, the variable-temperature (VT) ^1^H NMR spectra show temperature-dependent chemical shift changes that are most pronounced for the Bi-bound SiMe_3_ moiety together with a slight narrowing of the Si-bound SiMe_3_ (Figure S4). These observations support fluxional behavior of complex (*E*)-**1a** in solution, which may be due to conformational or ligand-based dynamics at the Bi center. Although DFT calculations predict an *E*/*Z* isomerization barrier of 6.6 kcal mol^–1^ (Figure S47), indicating that interconversion is theoretically feasible, the process is not sufficiently rapid to be detected on the NMR time scale under experimentally accessible conditions. To investigate whether steric congestion suppresses fluxionality, we synthesized and isolated the heteroleptic salt [KBi­(SiMe_3_)­(Si^*i*^Pr_3_)] in 68% yield via in situ formation of Bi­(Si^*i*^Pr_3_)­(SiMe_3_)_2_ (Figure S19). Subsequent reaction of [KBi­(SiMe_3_)­(Si^*i*^Pr_3_)] with LSi^II^Cl at −70 °C in diethyl ether yields L­(Me_3_Si)­Si=Bi­(Si^*i*^Pr_3_) ((*E*)-**1b**) in 60% yield as a red solid via stereoselective 1,2-migration of the SiMe_3_ group to silicon­(II), whereas the more sterically demanding Si^*i*^Pr_3_ substituent remains bound to Bi. The molecular structure of (*E*)-**1b** was established by scXRD analysis (Figure 1b). (*E*)-**1b** crystallizes in the monoclinic space group *P*2_1_/*n*, similar to (*E*)-**1a**, and the Si1 atom adopts the tetrahedral coordination geometry with a Si1–Bi1 distance of 2.543 (1) Å, which is only marginally longer than that of (*E*)-**1a**. However, the Si1–Bi–Si3 angle of 108.77(4)° in (*E*)-**1b** is about 10° larger than that of (*E*)-**1a** due to the steric congestion. The ^1^H NMR spectrum of (*E*)-**1b** exhibits a highly shielded Si–Si*Me*_*3*_ resonance (δ = 0.30 ppm) and overlapping Si^*i*^Pr_3_ signals (δ = 1.30–1.40 ppm). The lack of ^3^ *J*_H–H_ coupling is characteristic of isopropyl groups bound to heavier pnictogens.^41^ The room-temperature ^29^Si­{^1^H} NMR spectrum reveals three distinct signals: a sharp resonance at δ = −6.81 ppm (Me_3_ *Si*–Si=Bi), a broad resonance at 20.36 ppm (*Si*=Bi), and a deshielded resonance at 44.05 ppm (^*i*^Pr_3_ *Si*–Bi). This downfield shift, which mirrors the precursor, results from the bulky ^*i*^Pr_3_Si group enforcing a wider Si–Bi–Si angle.^42^ Unlike (*E*)-**1a**, VT ^1^H NMR spectra show no temperature-dependent shifts, which indicates that steric bulk suppresses the dynamics, demonstrating steric control over fluxionality in this Si=Bi compound. DFT studies also suggest a much higher rotational barrier compared to the parent system (Figure S47).

Reacting (*E*)-**1a** and (*E*)-**1b** with [W­(CO)_5_(thf)] afforded the terminal adducts (*Z*)-**2a** and (*Z*)-**2b** (Figures 2 and S38) in 50% and 47% yield, respectively. Notably, the coordination of the tungsten complex fragment induces *E* → *Z* isomerization of the silyl substituents (Scheme 2). While the *E* isomer is preferred in the “free” silylidenebismuthanes, coordination of the Lewis acidic [W­(CO)_5_] moiety to Bi provides the thermodynamic driving force to override the steric congestion and favor the *Z* configuration. Sc-XRD analysis revealed that the Si^II^ atom remains four-coordinate and tetrahedral in both cases, whereas the Bi atom is tricoordinated with pyramidal geometry (the sum of the bond angles around Bi1 is 315.91° for (*Z*)-**2a**) (Figures 2). The Si–Bi distance increases from about 2.5 Å in (*E*)-**1a**,**b** to 2.61–2.64 Å in the products, approaching common Si–Bi single-bond lengths.^43^ Structural and computational analyses further indicate substantial weakening of Si–Bi π interaction upon coordination due to a push–pull Si→Bi→W effect. The Bi–W distances of 2.9593(3) and 2.817(5) Å in (*Z*)-**2a**,**b** are in the range of related coordinative Bi→W bonds.^43^ The red-shifted ν_CO_ vibrations for (*Z*)-**2a** (2031–1875 cm^–1^) and (*Z*)-**2b** (2034–1878 cm^–1^) are comparable to those reported for the related Si=P tungten complex^44^ (ν_CO_ = 2054–1872 cm^–1^) and relative to [W­(CO)_5_(BiPh_3_)][Bibr ref45] (2074, 1942 cm^–1^) indicate strong ligand σ donation consistent with the weakened Si–Bi π bonding. In contrast to the formation of the [W­(CO)_5_] complexes, the reactions of (*E*)-**1a**,**b** with [Fe­(CO)_5_] result in Si=Bi bond scission to afford silylene–Fe­(CO)_4_ complex **3** (Scheme 2). For the reaction with (*E*)-**1a**, the known dibismuthane (Me_3_Si)_2_Bi–Bi­(SiMe_3_)_2_ ^34^ (Figure S39) was also formed and isolated in 20% yield along with elemental bismuth. Interestingly, the reaction of (*E*)-**1b** with [Fe­(CO)_5_] afforded few dark-black crystals of the cyclotetrabismuthane [BiSi^*i*^Pr_3_]_4_, whose connectivity was unambiguously established by scXRD analysis (Figure S42). This highlights substituent-dependent oligomerization of the bismuth fragments liberated during Si=Bi bond scission.

**Scheme 2:**
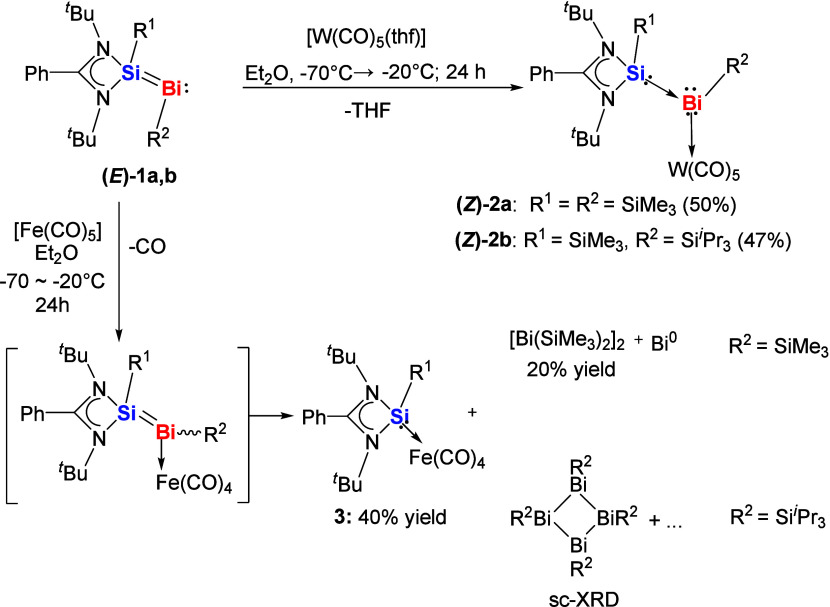
Coordination of (*E*)-**1a**,**b** with (top) [W­(CO)_5_] and (bottom) [Fe­(CO)_4_]

**Figure 2:**
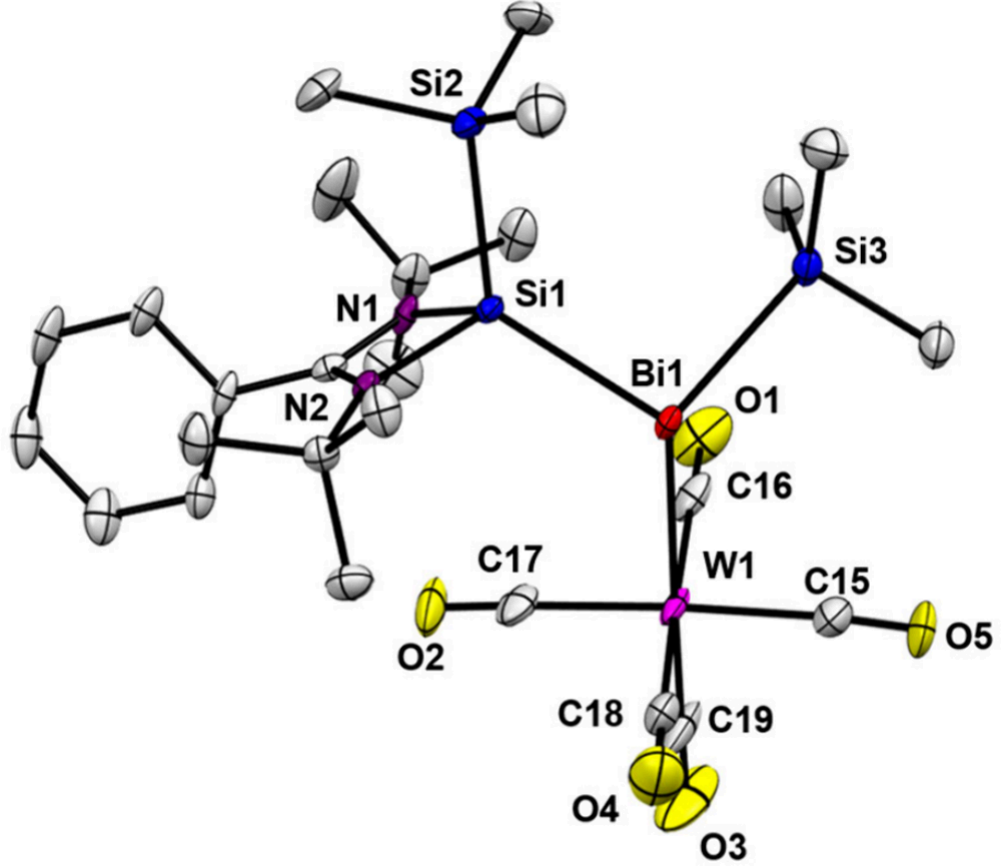
Molecular structure of complex (*Z*)-**2a** (50% probability ellipsoids; H atoms omitted for clarity).

To elucidate the electronic structures of (*E*)-**1a**,**b** and (*Z*)-**2a**,**b**, DFT calculations with ORCA 6.0 ^46^ were performed. The DFT-computed metric values for (*E*)-**1a**,**b** and (*Z*)-**2a**,**b** are in good agreement with the experimental data from scXRD analyses (Figures S44 and S45). The highest occupied molecular orbitals (HOMOs) of (*E*)-**1a**,**b** at the PBE0-D4/X2C-TZVPPall level of theory show a π-orbital interaction between the Si^II^ and Bi^I^ atoms with polarization toward the Bi atom, while the LUMO is a π* orbital with contribution of the Si^II^ site (Figures 3a, S49, and S50). In agreement with the presence of a polarized Si=Bi π bond, the Wiberg bond index (WBI) of (*E*)-**1a**,**b** amounts to 1.39 (Figure S48). As expected, the calculated WBI of 1.65 for the hypothetical (*E*)-silylidenebismuthane Me_2_N­(Me_3_Si)­Si=Bi­(SiMe_3_), containing a three-coordinate Si^II^ atom, is larger. The calculation of (*Z*)-**2a**,**b** revealed that coordination of Bi to the [W­(CO)_5_] complex fragment markedly diminishes the Si=Bi double bond character, resulting in a push–pull Si→Bi→W structure. This is consistent with the significantly lower WBI values of 1.06 and 1.05 as well as the elongation of the Si^II^–Bi bond distance as mentioned above.

**Figure 3:**
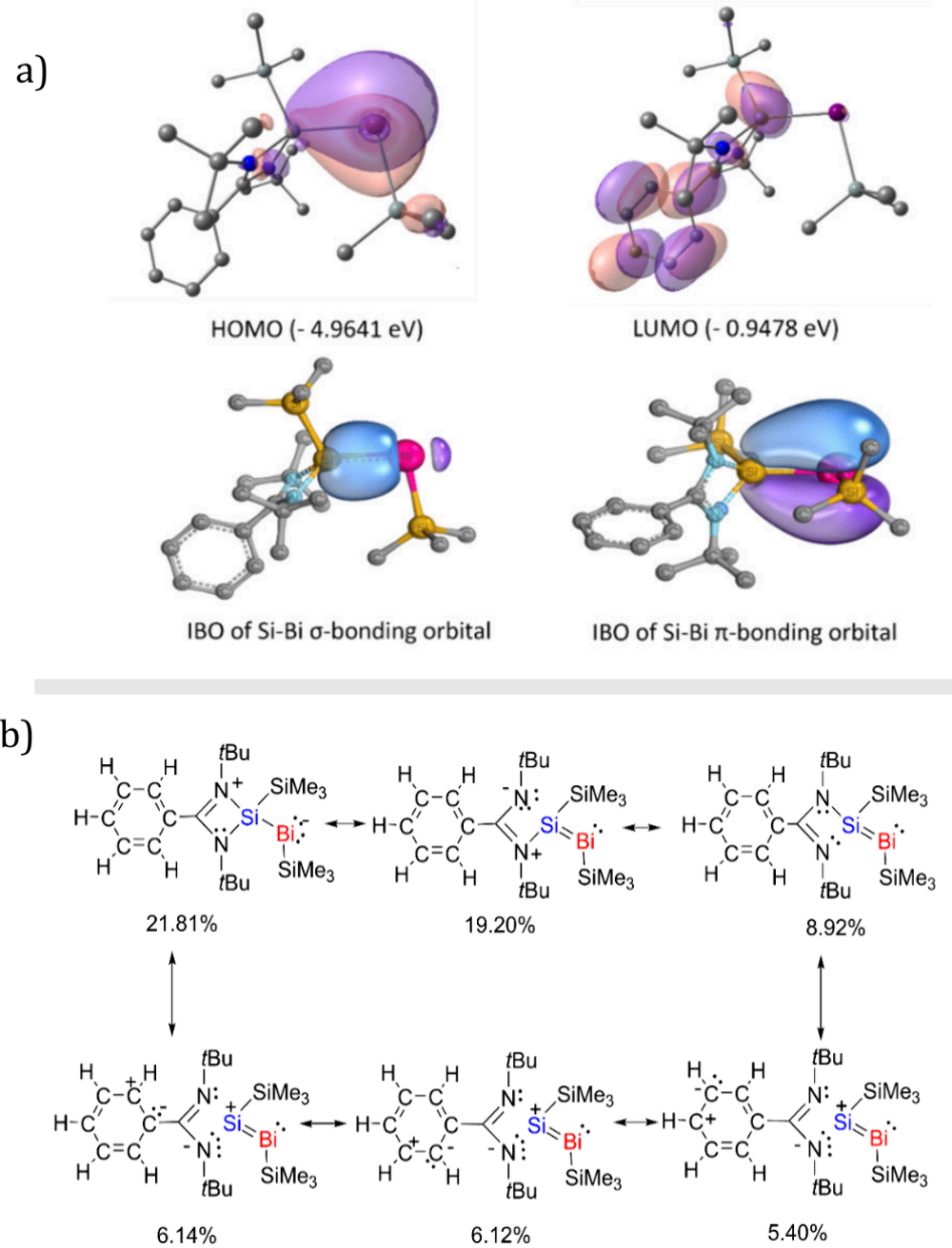
(a) Depiction of the DFT-derived HOMO and LUMO at the PBE0-D4/X2C-TZVPPall level and selected IBOs of (*E*)-**1a** at the PBE0-D4/def2-TZVP level (isovalue = 0.03). (b) Predominant resonance structures (weight > 5%) of (*E*)-**1a** calculated at the PBE0-D4/def2-TZVP level (see more in Scheme S7 and Table S17).

The WBI of 1.39 for (*E*)-**1a**,**b** is considerably larger than that for the dative Si^II^→Bi bonds in **IV** (1.17)^28^ and those in **V** (1.21, 1.24)^29^ (Figure S48). Intrinsic bond orbital (IBO)^47,48^ analysis confirms the presence of a Si=Bi double bond in (*E*)-**1a**,**b** consisting of σ and π orbitals that are strongly polarized toward the Bi atom (Figures 3a, S51, and S52). Moreover, the NBO and NLMO analyses reveal a σ bond arising from overlap between an sp-hybridized orbital on the central Si atom and a p-type orbital on Bi (Figures S53–S56), accompanied by a strongly polarized π interaction involving p orbitals on both atoms. The NPA charges in (*E*)-**1a** (Bi, −0.52; Si, +0.89) and (*E*)-**1b** (Bi, −0.49; Si, +0.87) indicate substantial charge separation, consistent with pronounced bond polarization (Figure S48). Second-order perturbation analysis of (*E*)-**1a**,**b** identifies a donor–acceptor interaction from a filled p-type orbital on bismuth into an acceptor orbital on the central silicon, with a stabilization energy of 16.9 kcal mol^–1^. This interaction accounts for the additional π-type contribution and the resulting partial Si=Bi double-bond character. Furthermore, natural resonance theory (NRT) analysis of (*E*)-**1a** reveals that the dominant resonance structures can be classified into two types featuring Si=Bi and Si–Bi bond character (Figure 3b; see more in Scheme S7 and Table S17), pointing out a larger weight of the Si=Bi bond character.

In summary, we have isolated the first neutral silylidenebismuthanes (*E*)-**1a**,**b** realized by a 1,2 silyl group migration. DFT analysis shows a highly polarized Si=Bi (zwitterionic) bond with electron density shifted toward bismuth. NMR studies indicate that increasing bismuth’s steric demand suppresses the fluxional behavior seen in (*E*)-**1a**. Notably, [W­(CO)_5_] coordination induces the *E* → *Z* transformation while [Fe­(CO)_4_] coordination causes bond scission. Further reactivity studies and application of (*E*)-**1a**,**b** to serve as Bi^I^(silyl) transfer reagents are currently in progress.

## Supplementary Material



## Notes

Additional experimental details, materials, spectroscopic data, and DFT results (PDF)
