# Supplementary material for: Taming Neutral Silylidenebismuthanes (Si=Bi) with a Low-Coordinate Bismuth Atom
Source: J Am Chem Soc. 2026 Jun 25;148(26):27066–71. doi: 10.1021/jacs.6c10667 (PMC13352506; doi:10.1021/jacs.6c10667)
Supplement: Supplementary file 1 [file ja6c10667_si_001.pdf]

Supporting Information

Taming Neutral Silylidenebismuthanes (Si=Bi) with a Low-Coordinate Bismuth Atom

Annapurna Saxena, Shenglai Yao, Jan Dirk Epping and Matthias Driess*

Table of Content

A. Experimental Section……………………………………….………………...…..……...S2

A1 General Considerations……………………………………….……….…….……….……S2

A2 Single-Crystal X-ray Structure Determination………………………..…..….……….…..S2

A3 Synthesis and Characterization………………………………….……………..……….….S3

A4 Details of the Single Crystal X-ray Diffraction Analyses…………………….……..……..…S43

A5 UV-Visible Spectra ………………………………….…….….….……….…….......……..S59

B. Computational Details …………….…………….……………….….….….………....……..S60

C. References……………………………………………………….……….….….…...…....……..S76

A. *Experimental Section*

A1. General Considerations

All experiments were carried out under dry oxygen-free nitrogen using standard Schlenk techniques or an MBraun glove box fitted with a gas purification and recirculation unit. Solvents were dried by standard methods and freshly distilled prior to use. The starting material, LSiCl [L = PhC(NtBu)2] was prepared according to the literature procedure.[1] $[W(CO)_6]$ was purchased from Thermo Scientific Fischer and was sublimed before use. The NMR spectra were recorded with Bruker spectrometers Avance II 400, Avance III 400, and Avance III 500 referenced to residual solvent signals as internal standards. Abbreviations: s = singlet; d = doublet; t = triplet; sept = septet; m = multiplet; br = broad. High-resolution ESI-MS were measured on a Thermo Scientific LTQ orbitrap XL. IR spectra were measured with a Nicolet iS5 FT-IR-Spectrometer from the company of Thermo Scientific. Melting points were measured on a Stuart SMP30 melting point apparatus. UV/Vis spectra were recorded on an Analytik Jena Specord S600 diode array spectrometer.

A2. Single-Crystal X-ray Structure Determination

The crystals were mounted on a glass capillary in per-fluorinated oil and measured in a cold N_2 flow. The data of ***(E)*-1a, *(E)*-1b *(Z)*-2a**, ***(Z)*-2b**, **3**, $[K(2.2.2\text{-cryptand})Bi(SiMe_3)_2]$ and$[K(2.2.2\text{-cryptand})Bi(SiMe_3)(Si^iPr_3)]$ were collected on an Oxford Diffraction Supernova, Single source at offset, Atlas at 150 K (Cu- Kα-radiation, λ = 1.5418 Å). The structures were solved with the SHELXT[2] and refined with Olex2[3,4] software package. For the crystal of ***(Z)*-2b**, residual electron density was observed close to the center of the bismuth, which may be due to anharmonic displacement of the heavy metal atoms. **CCDC**: 2500648 **((*E*)-1a**), 2555120 (**(*E*)-1b**), 2500647 (**(*Z*)-2a**), 2555119 (**(*Z*)-2b**), 2500649 **(3),** 2500650 **($[K(2.2.2\text{-cryptand})Bi(SiMe_3)_2]$),** 2555122 **($[K(2.2.2\text{-cryptand})Bi(SiMe_3)(Si^iPr_3)]$)**, and 2555121 **$[Bi_4(Si^iPr_3)_4]$)** contain the supplementary crystallographic data for this paper. These data can be obtained free of charge from The Cambridge Crystallographic Data Centre via www.ccdc.cam.ac.uk/structures/

A3. Synthesis and Characterization

Scheme S1. Synthesis of compound ***(E)*-1a.**

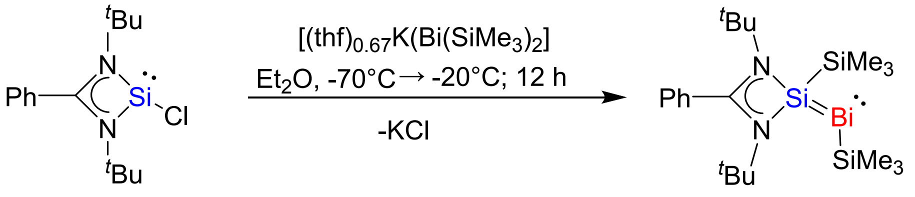

***(E)*-1a** Yeild: 55%

Preparation of [K(2.2.2-cryptand)Bi(SiMe$_3$)$_2$] **:** [KBi(SiMe$_3$)$_2$.(thf)$_{0.67}$] was synthesized as per the literature procedure.[5] KBi(SiMe$_3$)$_2$.(thf)$_{0.67}$ (100 mg, 0.244 mmol) was dissolved in 2 mL of THF. 2.2.2-cryptand (91.8 mg, 0.244 mmol) was also dissolved in 2 mL of THF, and this solution was slowly added to [KBi(SiMe$_3$)$_2$.(thf)$_{0.67}$] solution at -70°C without stirring and was kept at -20°C overnight. After 12 h, the solvent was removed, and ether (1 mL) was slowly added to obtain [K(2.2.2-cryptand)Bi(SiMe$_3$)$_2$] as greenish yellow crystals at room temperature, which was suitable for X-ray crystallography. (Fig. S40).

Synthesis of compound (E)-1a**:** In a 100 mL Schlenk flask, chlorosilylene LSiCl(1 g, 3.3 mmol) was dissolved in 10 mL of diethyl ether, and the solution was kept at -70ºC in an acetone bath. To this, a greenish-yellow solution of [KBi(SiMe$_3$)$_2$.(thf)$_{0.67}$] (1.45 g, 3.3 mmol) in 10 mL of diethyl ether was added dropwise over a period of 15 min without stirring. On addition of greenish yellow solution of [KBi(SiMe$_3$)$_2$.(thf)$_{0.67}$] to colourless solution of chlorosilylene, formation of dark red solution was observed. This solution was further stored at -20ºC for 14 h. Formation of dark red crystals can be observed on the flask, which were suitable for X-ray measurement. The solution was then filtered, and the solvent was removed under vacuum. The red residue was washed with cold pentane (2 x 2mL), and the pure complex ***(E)-1a*** was isolated and characterized. The pentane solution was further concentrated and stored at -20 °C, yielding a second fraction of red crystals. Yield = 55% (1.13 g, 1.81 mmol).

M.p. 120.4 ºC (decomp.).

^{1}H NMR (400 MHz, C$_6$D$_6$, 298K): δ/ppm = 0.37 (s, 9 H, SiSi*Me$_3$*), 1.16 (s, 18 H, NC(C*H$_3$*)$_3$), 1.28 (s, 9 H, BiSi*Me$_3$*), 6.83-7.00 (m, 4 H, Ph-*H*), 7.20 (d, J = 8 Hz, 1H, *o*-Ph-*H*).

^{13}C{^{1}H} NMR (125 MHz, C$_6$D$_6$, 298K): δ/ppm = -0.42 (s, SiSi*Me$_3$*), 12.19 (s, BiSi*Me$_3$*), 32.31 (s, C*Me$_3$*), 55.29 (s, *C*Me$_3$), 127.99, 128.60, 128.89, 130.25, 130.73, 134.16 (Ph), 166.51 (s, N*C*N).

^{29}Si{^{1}H} NMR (99 MHz, C$_7$D$_8$, 203 K): δ/ppm = -28.41 (Bi*Si*Me$_3$), -7.61 (Si*Si*Me$_3$), 37.48 (*Si*=Bi).

^{1}H,^{29}Si-HMQC NMR (400 MHz, C$_7$D$_8$, 203 K, J_{SiH} = 7 Hz, NUS 50%) (δ_H, δ_{Si})/ppm = (1.41, -28.655), (0.42, -7.699), (0.42, 37.959).

IR (cm^{-1}): 2960.84(m), 2883.58(w), 1973.41(br), 1576.99(w), 14569.69(m), 1444.54(w), 1392.49(s), 1364.03(s), 1270.39(m), 1230.47(w), 1241.24(s), 1197.76(m), 1182.95(w), 1159.49(w), 1083.51(w), 1032.61(w), 1016.60(w), 984.10(w), 926.09(w), 823.17(s), 797.28(s), 757.66(s), 736.65(w), 708.48(m), 678.49(m).

HR-ESI-MS: (m/z): calcd for (C$_{21}$H$_{41}$BiN$_2$Si$_3$)$^+$ [M-H]$^+$: 614.23759; found: 614.23819.

UV-Visible: λ_{max} = 432 nm; λ_{max} = 325 nm.

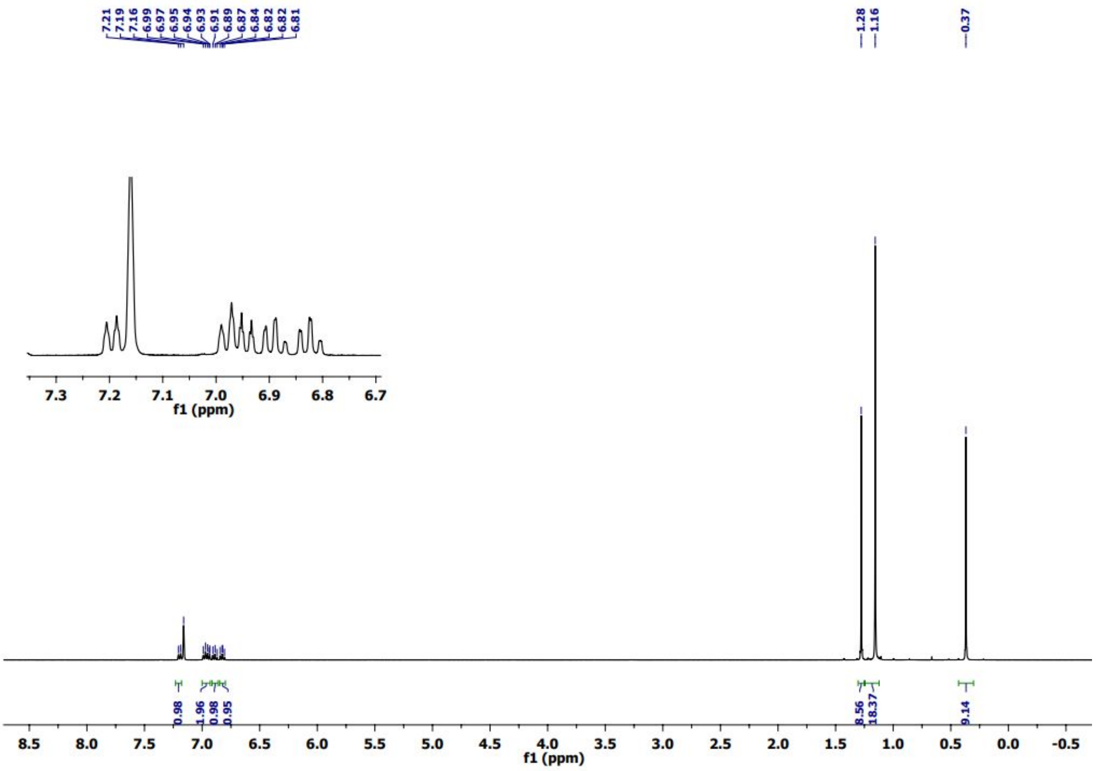

Figure S1. ^{1}H-NMR spectrum of compound ***(E)*-1a** (400 MHz, C_6D_6, 298K)

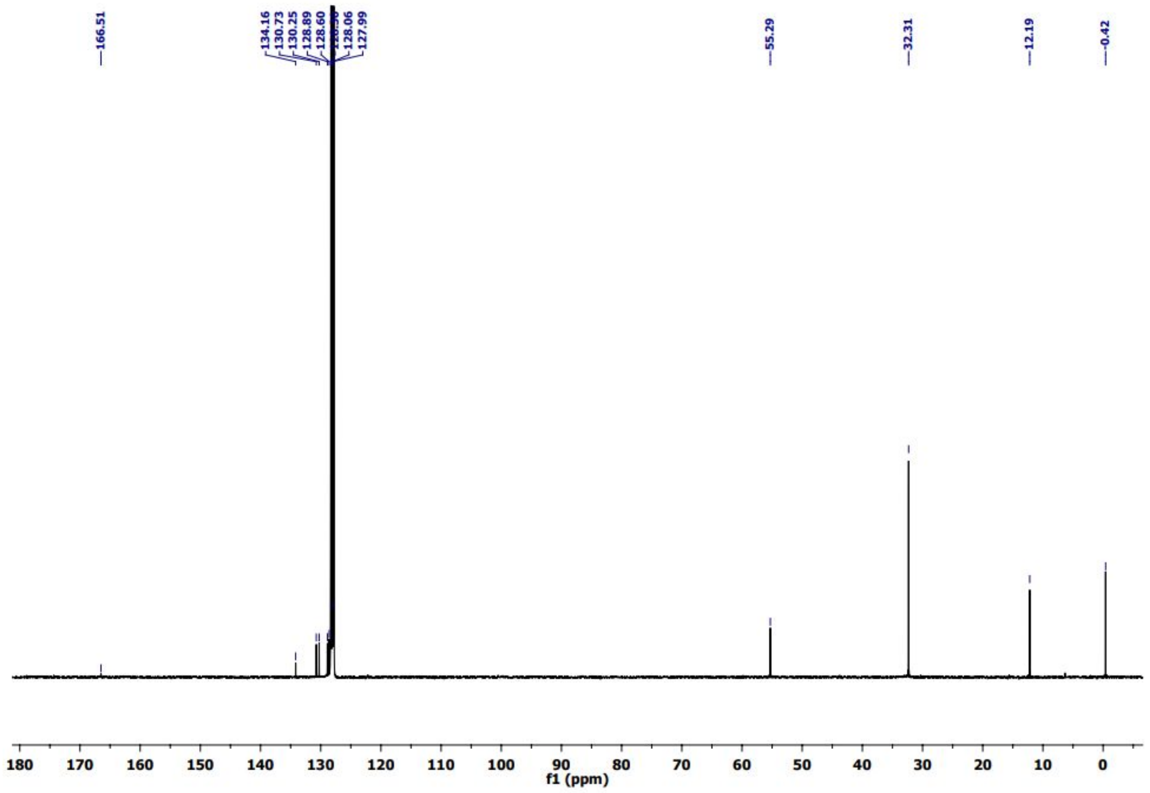

Figure S2. $^{13}C\{^{1}H\}$-NMR spectrum of compound ***(E)*-1a** (125 MHz, C_6D_6, 298 K).

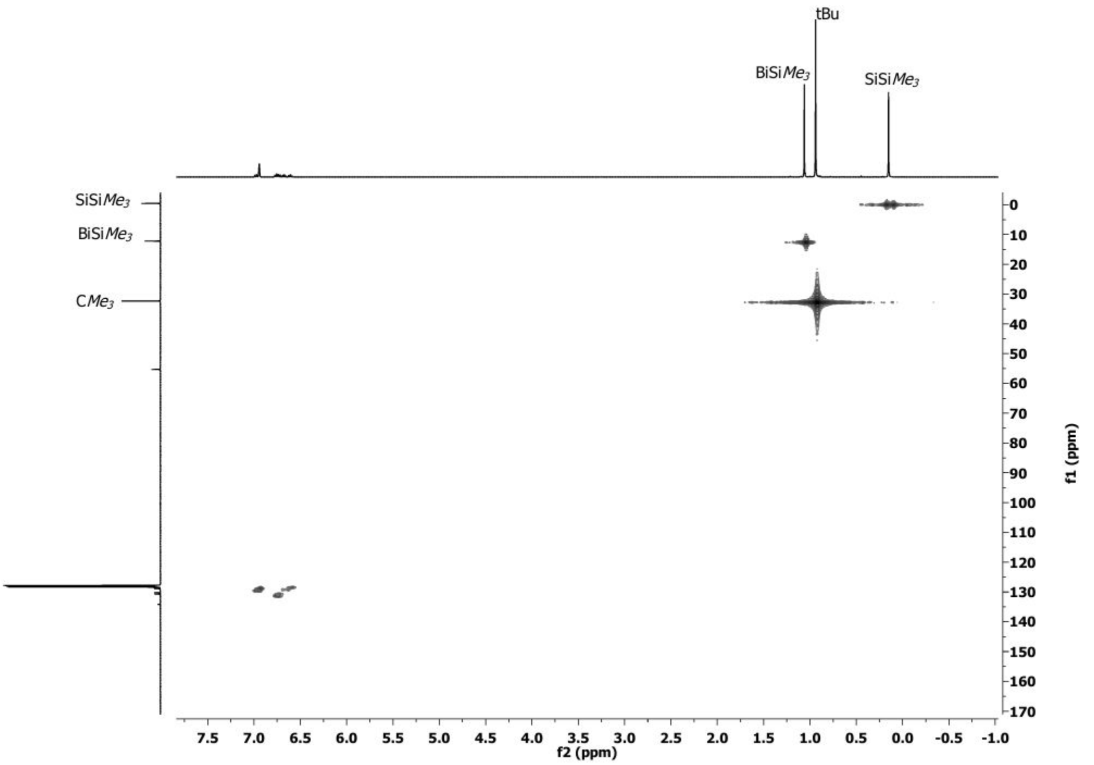

Figure S3. $^{1}H,^{13}C$ HMQC NMR spectrum of compound ***(E)*-1a** (125 MHz, C_6D_6, 298 K).

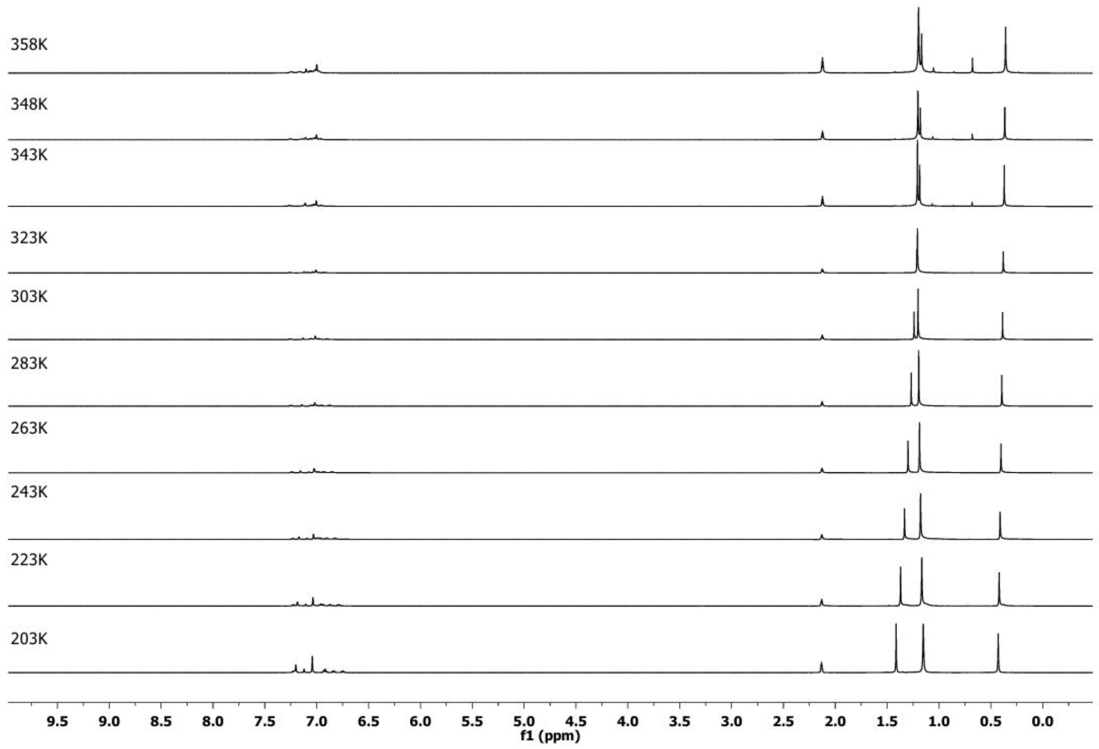

Figure S4. Variable temperature ^{1}H-NMR spectrum of compound ***(E)*-1a** (500 MHz, C_7D_8, 203 – 358K).

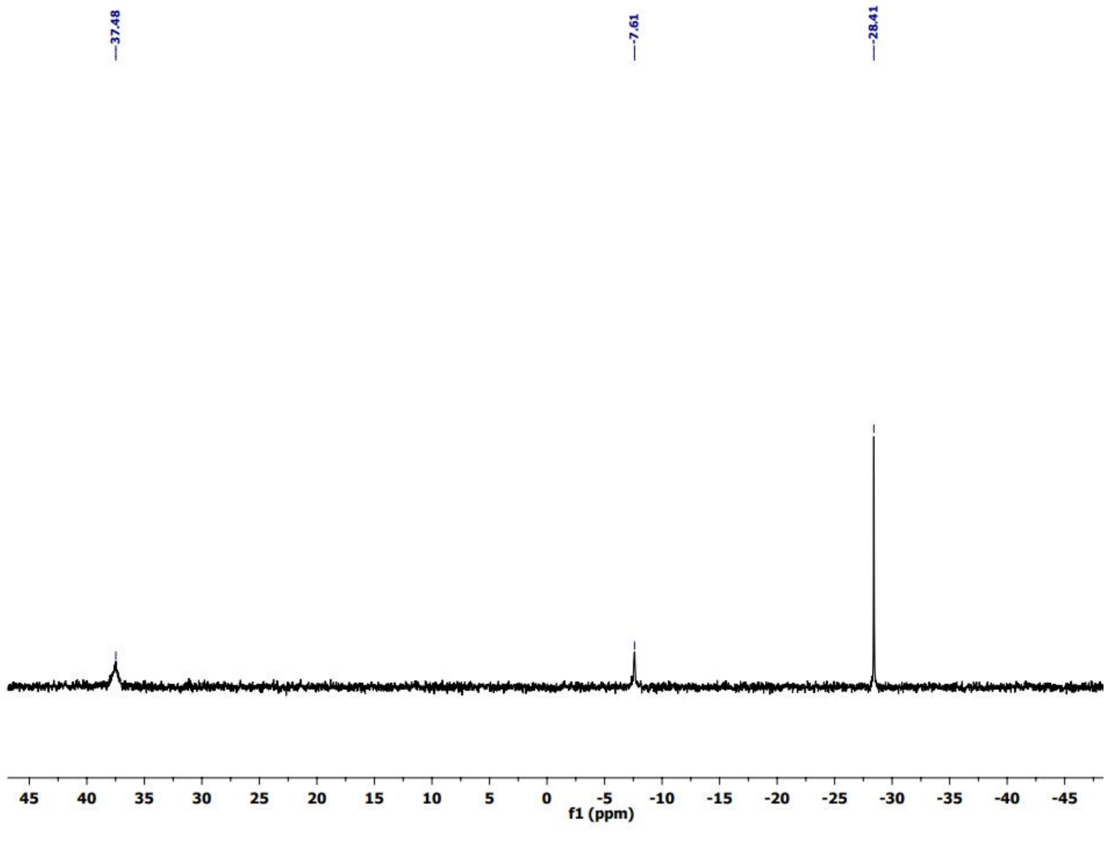

Figure S5. $^{29}Si\{^{1}H\}$-NMR spectrum of compound ***(E)*-1a** (99 MHz, C_7D_8, 203 K)

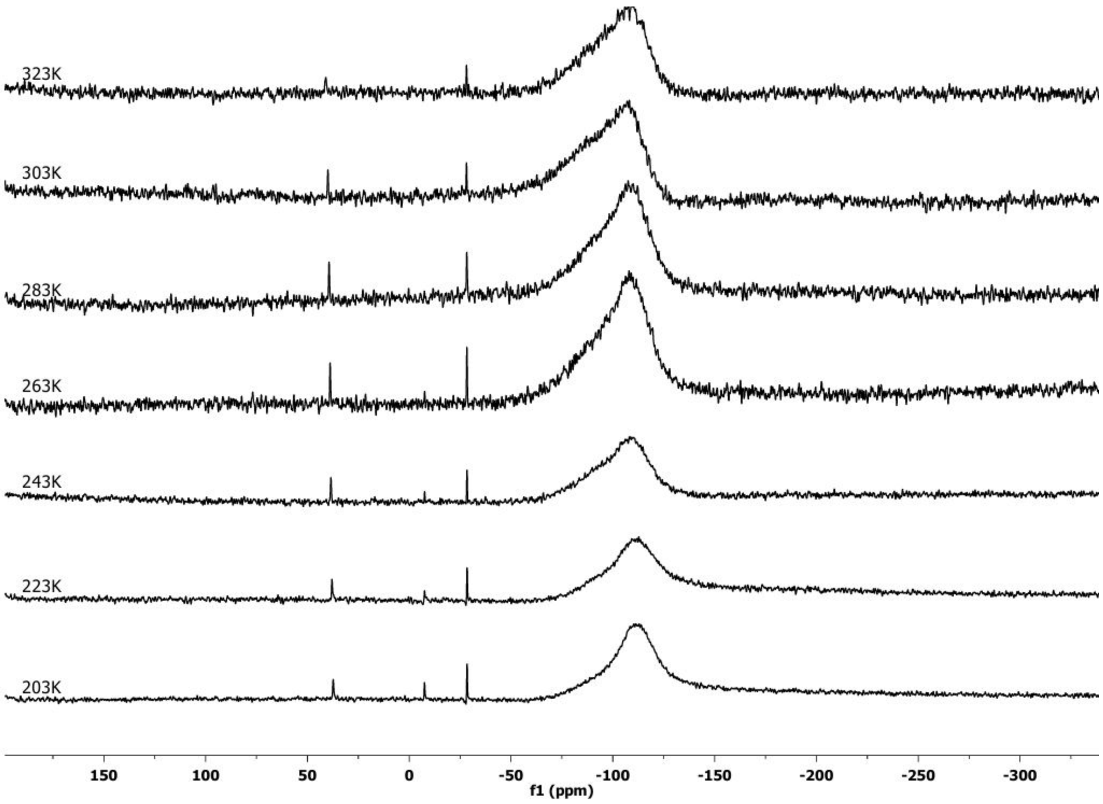

Figure S6. Variable temperature ^{29}Si-NMR spectrum of compound ***(E)*-1a** (500 MHz, C_7D_8, 203 – 323K).

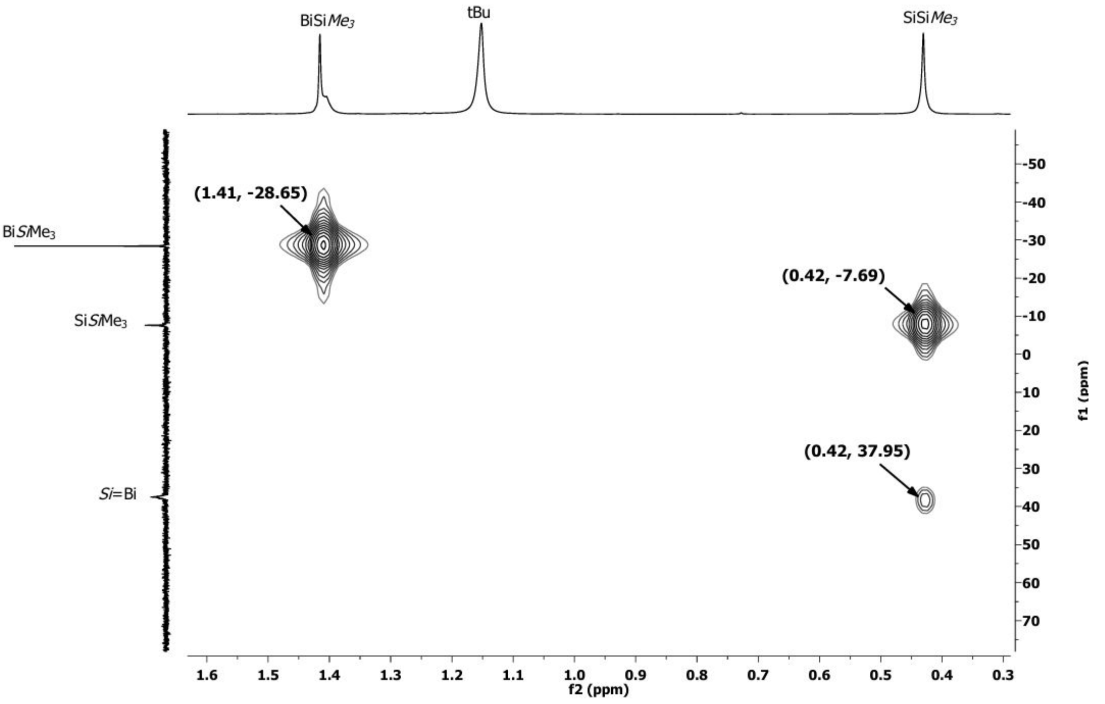

Figure S7. ^{1}H,^{29}Si-HMQC NMR Spectrum of compound ***(E)*-1a** (500 MHz $J_{Si\text{-}H}$ = 7Hz, C_7D_8, 203 K)

NOTE: The spectra has been zoomed in to show better correlation of peaks.

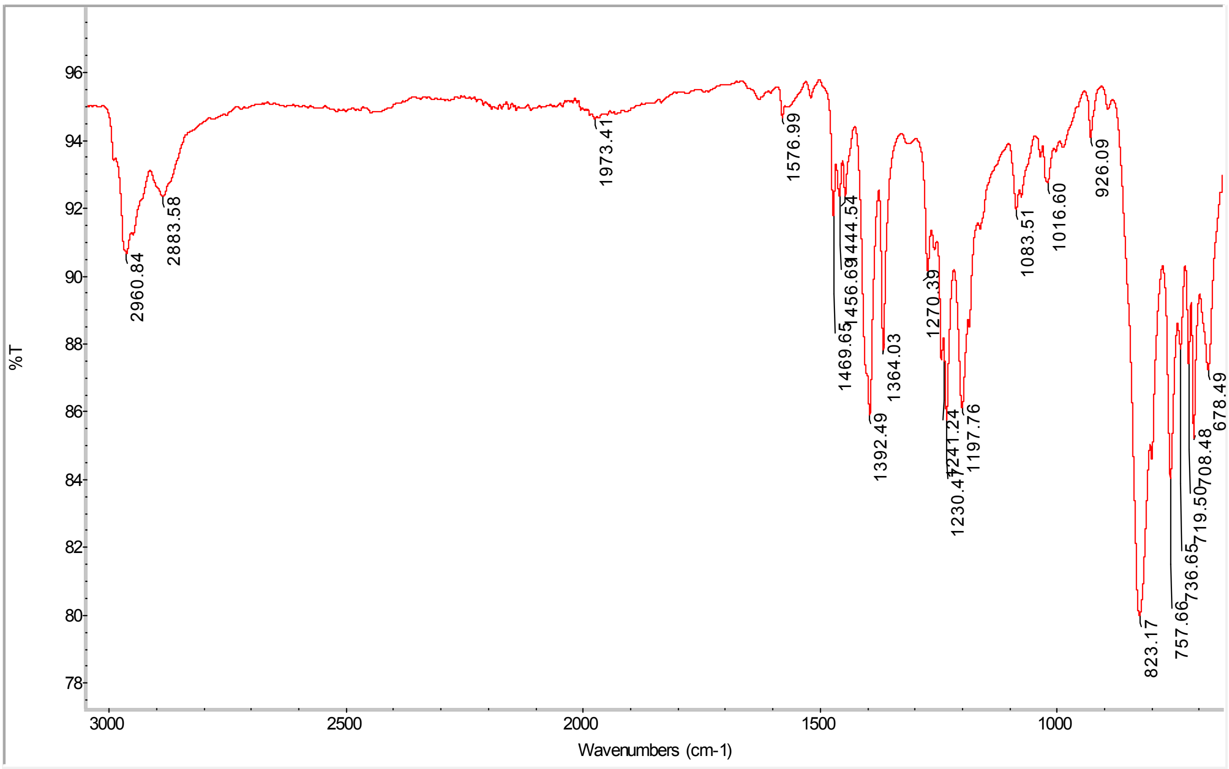

Figure S8. IR spectrum of compound ***(E)*-1a**

Scheme S2. Synthesis of compound ***(Z)*-2a**.

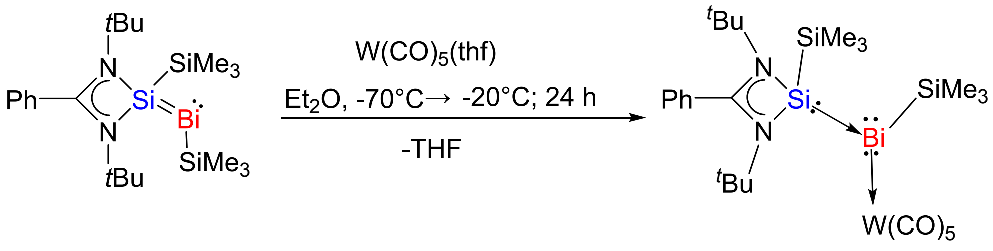

Synthesis of compound (Z)-2a*:** In a 100 mL Schlenk flask [W(CO)$_6$] (114 mg, 0.324 mmol) was stirred in 5 mL of THF and irradiated with UV light (320-400 nm) for 4 h under N_2 flow to obtain a freshly prepared yellow solution of [W(CO)$_5$(thf)] in situ. The freshly prepared [W(CO)$_5$(thf)] was further added to a solution of complex ***(E)-1a (200 mg, 0.324 mmol) in 10 mL of ether at -70ºC without stirring. The reaction mixture was then kept at -20ºC for 2 days. After 2 days, some amount of black precipitate corresponding to elemental bismuth was observed; thus, the solution was filtered, and the solvent was evacuated to obtain a brownish-black residue. This residue was washed with 4-5 times (2-5 mL) with cold pentane to remove any unreacted [W(CO)$_6$] and the product was extracted from toluene (4 mL). The toluene solution was concentrated and kept at -20 ºC overnight to obtain orange colour crystals suitable for X-ray measurements. Toluene was decanted and the crystals were further washed with cold pentane to obtain pure compound ***(Z)-2a***. Yield = 50% (152 mg, 0.162 mmol).

M.p. 118.6 ºC (decomp.).

^{1}H NMR (400 MHz, C_6D_6, 298K): δ/ppm = 0.20 (s, 9 H, SiSi*Me$_3$*), 0.97 (s, 9 H, BiSi*Me$_3$*), 0.99 (s, 18 H, NC(C*H$_3$*)$_3$), 6.79-6.83 (m, 1H, Ph-*H*), 6.90-6.96 (m, 3H, Ph-*H*), 7.41 (d, J = 8 Hz, 1H, *o*-Ph-*H*).

^{13}C{^{1}H} NMR (125 MHz, C_6D_6, 298K): δ/ppm = -1.11 (s, SiSi*Me$_3$*), 8.15 (s, BiSi*Me$_3$*), 31.62(s, C*Me$_3$*), 55.33 (s, *C*Me$_3$), 128.79, 128.97, 129.39, 130.99, 131.55 (Ph) 168.50 (s, N*C*N), 203.94 (s, W(*C*O)$_5$).

^{29}Si{^{1}H} NMR (99 MHz, C_6D_6, 298K): δ/ppm = -29.34 (Si*Si*Me$_3$), -26.48 (Bi*Si*Me$_3$), -12.48 (Si=Bi).

^{1}H,^{29}Si-HMQC NMR (400 MHz, C_6D_6, 298 K, J_{SiH} = 7 Hz, NUS 50%) (δ_H, δ_{Si})/ppm = (0.20, -29.436), (0.98, -26.683), (0.20, -12.970).

IR (cm^{-1}): 2968.86(w), 2031.81(m), 1939.86(w), 1893,11(s) 1875.27(s), 1605.73(w), 1469.66(w), 1391.90(m), 1367.16(m), 1273.99(w), 1238.10(w), 1198.49(w), 831.92(s), 800.35(w), 763.57(m), 740.92(w), 704.13(m), 721.94(m).

HR-ESI-MS: m/z: calcd for ($C_{26}H_{42}BiN_2O_5Si_3W$)$^+$ [M]$^+$: 938.16309; found: 938.16387

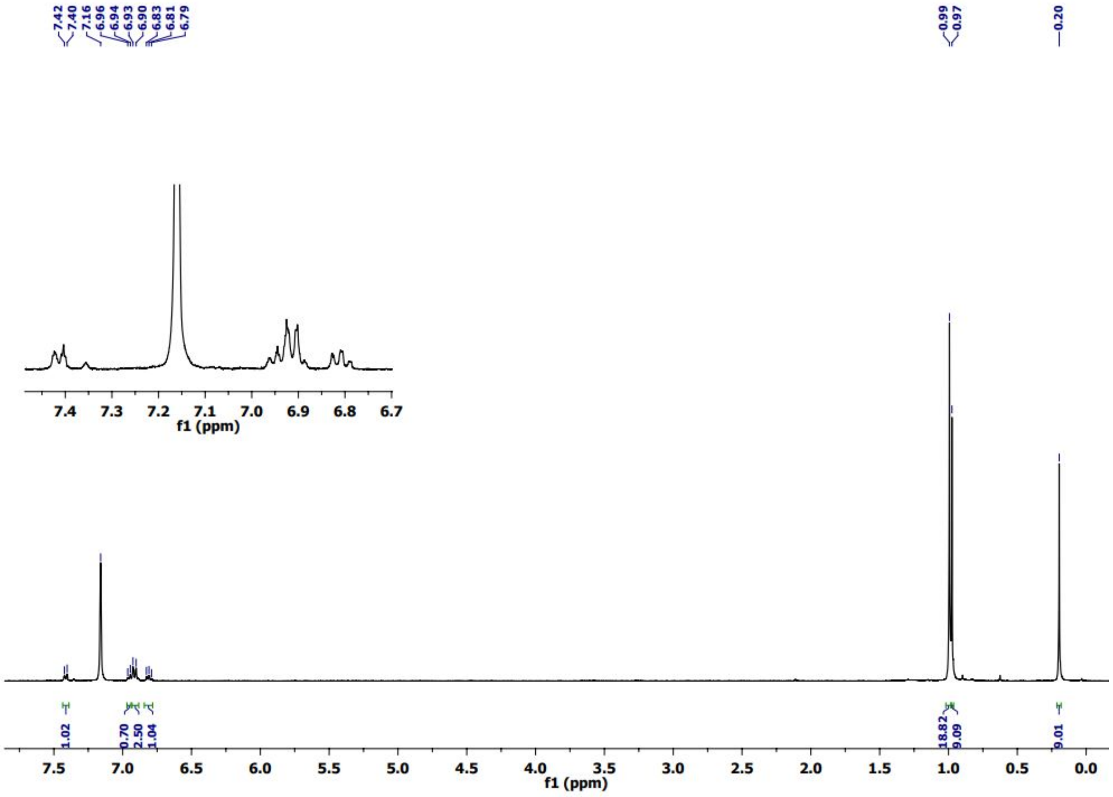

Figure S9. ^{1}H-NMR spectrum of compound ***(Z)*-2a** (400 MHz, C_6D_6, 298K)

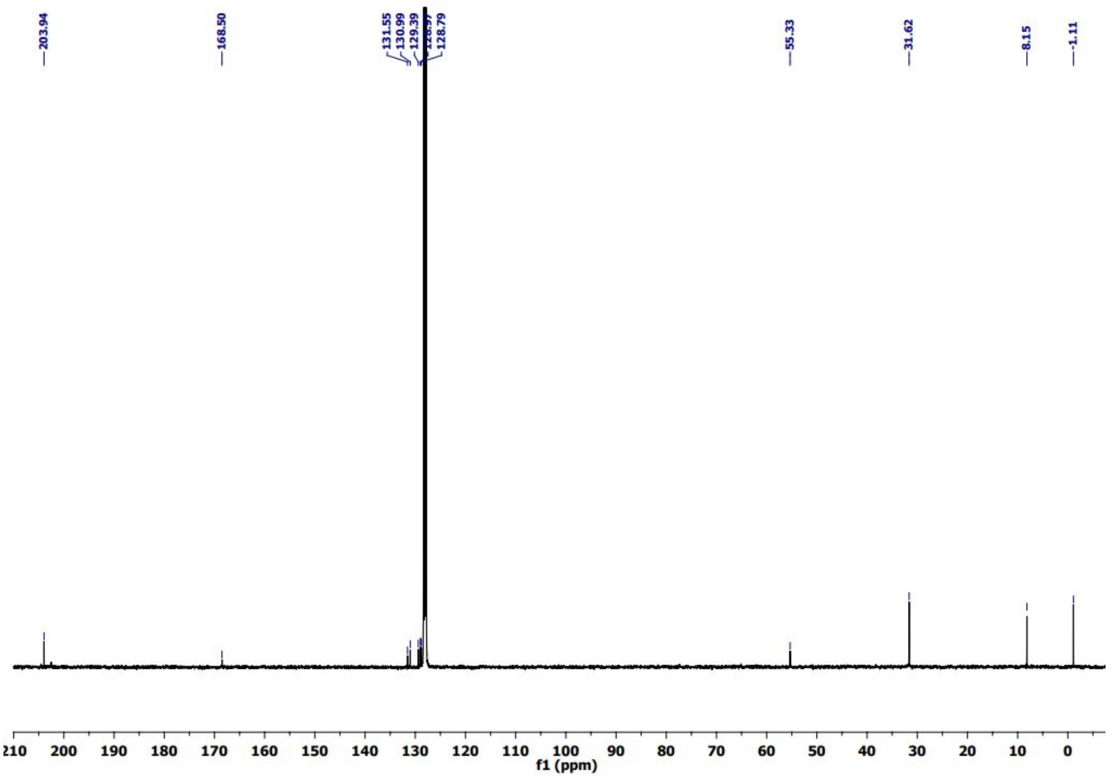

Figure S10. ^{13}C-NMR spectrum of compound ***(Z)*-2a** (125 MHz, C_6D_6, 298K)

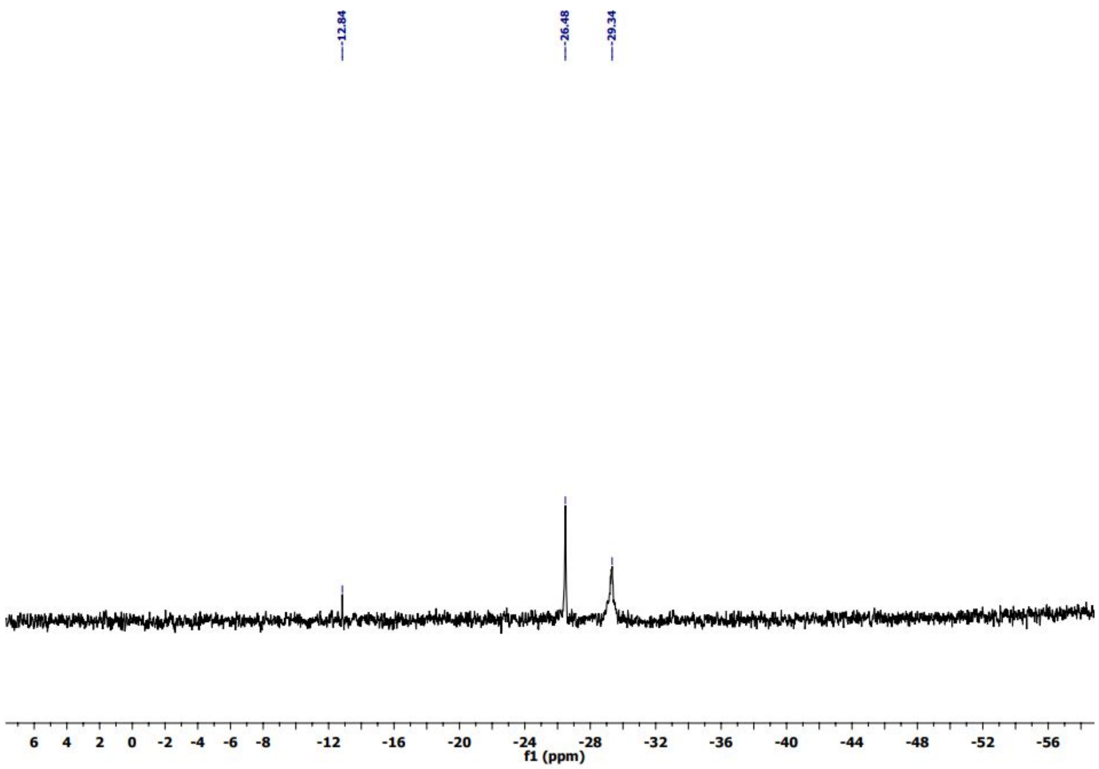

Figure S11. $^{29}Si\{^{1}H\}$-NMR spectrum of compound ***(Z)*-2a** (99 MHz, C_6D_6, 298 K)

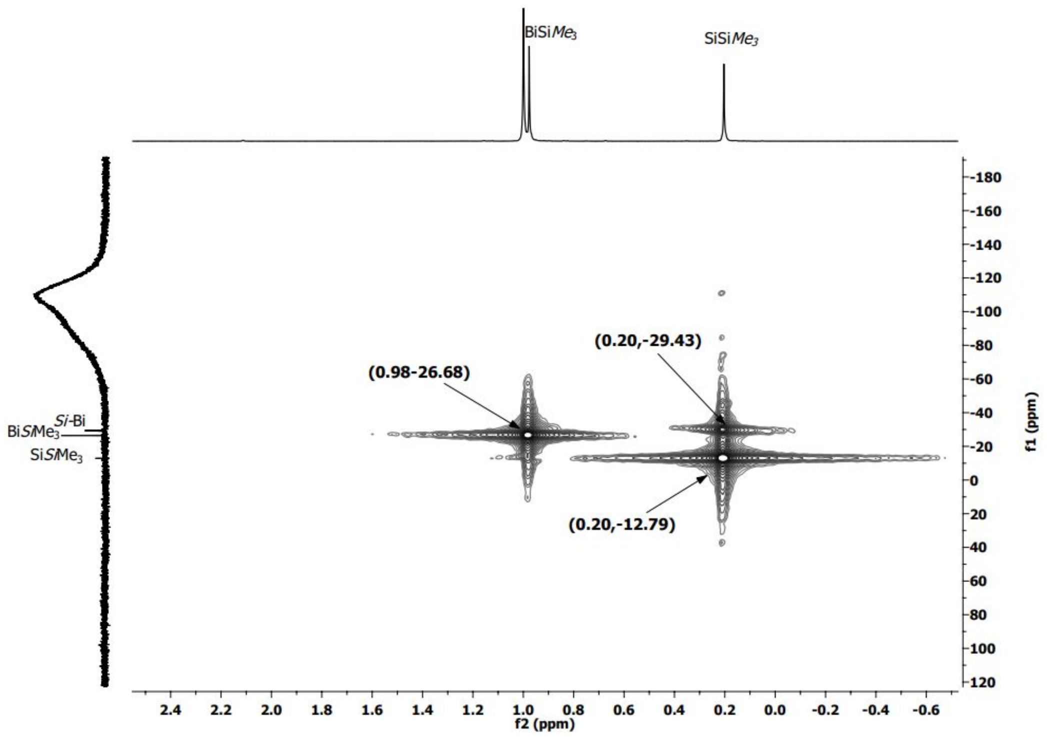

Figure S12. ^{1}H, ^{29}Si-HMQC NMR Spectrum of compound ***(Z)*-2a** (400 MHz J_{Si-H} = 7Hz, C_6D_6, 298 K)

NOTE: The spectra has been zoomed in to show better correlation of peaks.

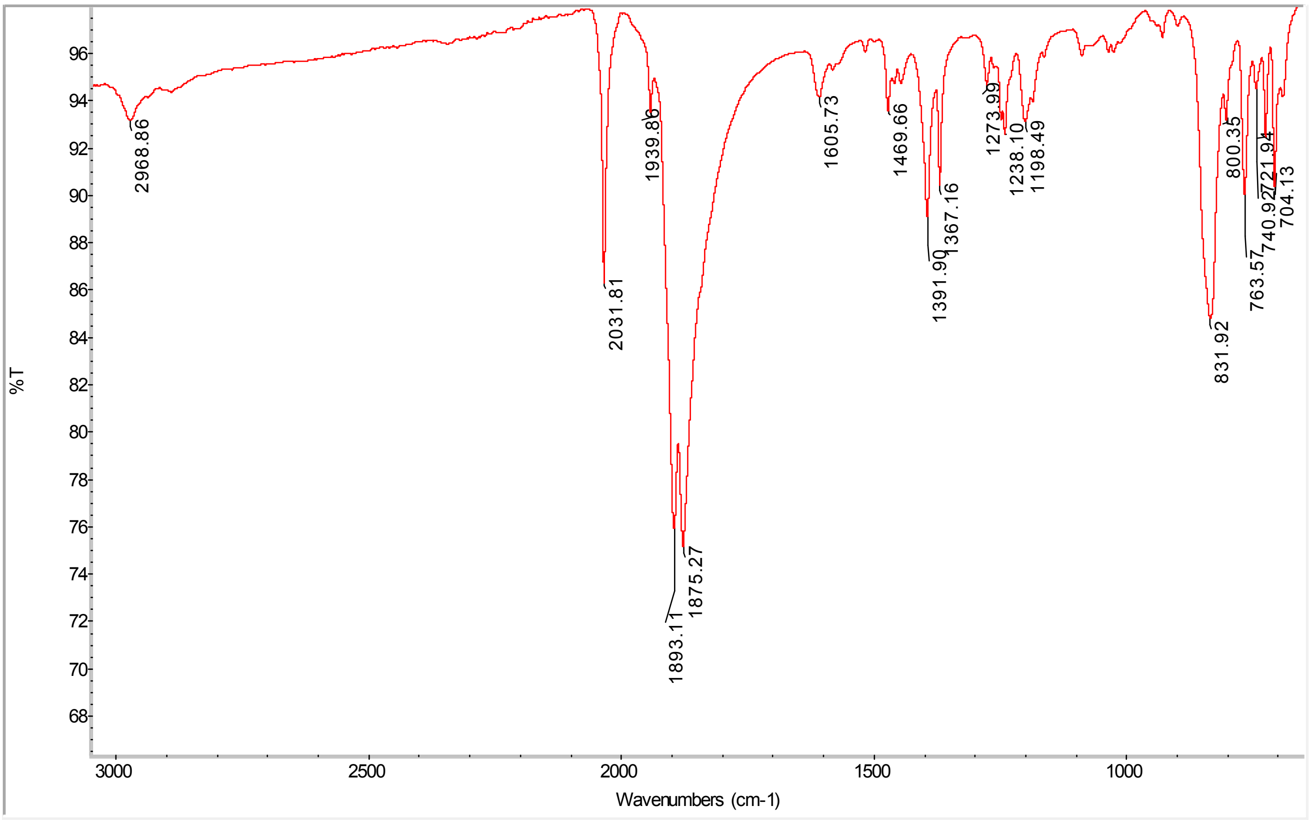

Figure S13. IR spectrum of compound ***(Z)*-2a**.

Scheme S3. Synthesis of compound **3**.

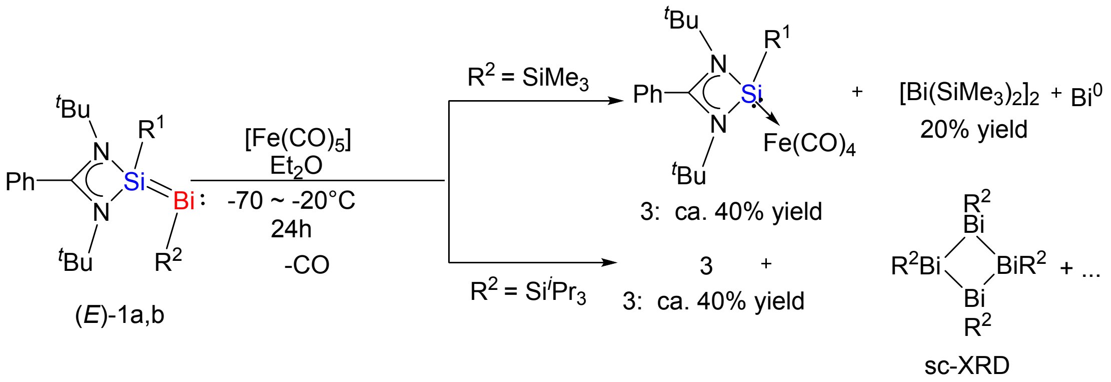

Synthesis of compound 3: In a 100 mL Schlenk flask, compound ***(E)-1a*** (100 mg, 0.162 mmol) was dissolved in 5 mL of ether. To this solution [Fe(CO)$_5$] (0.021 mL, 0.162 mmol) previously dissolved in 5 mL of ether was added dropwise at -70ºC solution without stirring. The reaction mixture was further kept at -20 ºC for 24 h. Over a period of 24 h, black ppt was observed which was removed through filtration. Solvent was further removed and the residue was washed with cold pentane(2ml). The pentane solution was concentrated and kept at -20 ºC to obtain (Me$_3$Si)$_4$Bi$_2$ as green crystals. The residue was again dissolved in ether to obtain colourless crystals suitable for X ray crystallography. The solvent was decanted and pure product was isolated as white solid. Yield = 40% (32.5 mg, 0.0648 mmol). For ***(E)-1b*** (100 mg, 0.143 mmol) the same reaction was performed under similar conditions. After 24 h, few black crystals at the bottom of the flask were obtained, these crystals were analyzed through X ray crystallography which confirmed the formation of [Bi$_4$(Si^{i}Pr)$_4$], the ether solution was carefully decanted, concentrated and kept at-20ºC. After 2 days colourless crystals of ***3*** were obtained. Yield 40% (28.6 mg, 0.0572 mmol). Several attempts to characterize [Bi$_4$(Si^{i}Pr)$_4$] were unsuccessful due to highly sensitive nature of the compound.

M.p. 109.4 ºC (decomp.).

^{1}H NMR (400 MHz, C$_6$D$_6$, 298K): δ/ppm = 0.42 (s, 9 H, SiSi*Me$_3$*), 1.03 (s, 18 H, NC(C*H$_3$*)$_3$), 6.80-6.83(m, 2H, Ph-*H*) 6.90-6.96 (m, 2H, Ph-*H*), 7.41 (d, *J* = 8 Hz, 1H, *o*-Ph-*H*).

^{13}C{^{1}H} NMR (125 MHz, C$_6$D$_6$, 298K): δ/ppm = -0.22(s, SiSi*Me$_3$*), 31.51(s, C*Me$_3$*), 54.76 (s, *C*Me$_3$) 141.67, 128.86, 129.68, 130.59, 131.71 (s, Ph), 166.96(s, N*C*N), 216.92 (Fe(*C*O)$_4$).

^{29}Si{^{1}H} NMR (99 MHz, C$_6$D$_6$, 298K): δ/ppm = -11.46 (Si*Si*Me$_3$), 120.92 (*Si*SiMe$_3$).

^{1}H,^{29}Si-HMQC NMR (400 MHz, C$_6$D$_6$, 298 K, J_{SiH} = 7 Hz, NUS 50%) (δ_H, δ_{Si})/ppm = (0.426, -11.4), (0.426, 120.9).

IR (cm^{-1}): 2018.86(s), 1943.85(s), 1905.88(s), 1891.01(s), 844.52(m), 764.16(w), 727.91(w), 705.65(m), 626.44(s), 617.29(s),

HR-ESI-MS: m/z: calcd for $(C_{22}H_{33}FeN_2O_4Si_2)^+$ [M]$^+$: calc. 501.13228 found: 501.13161.

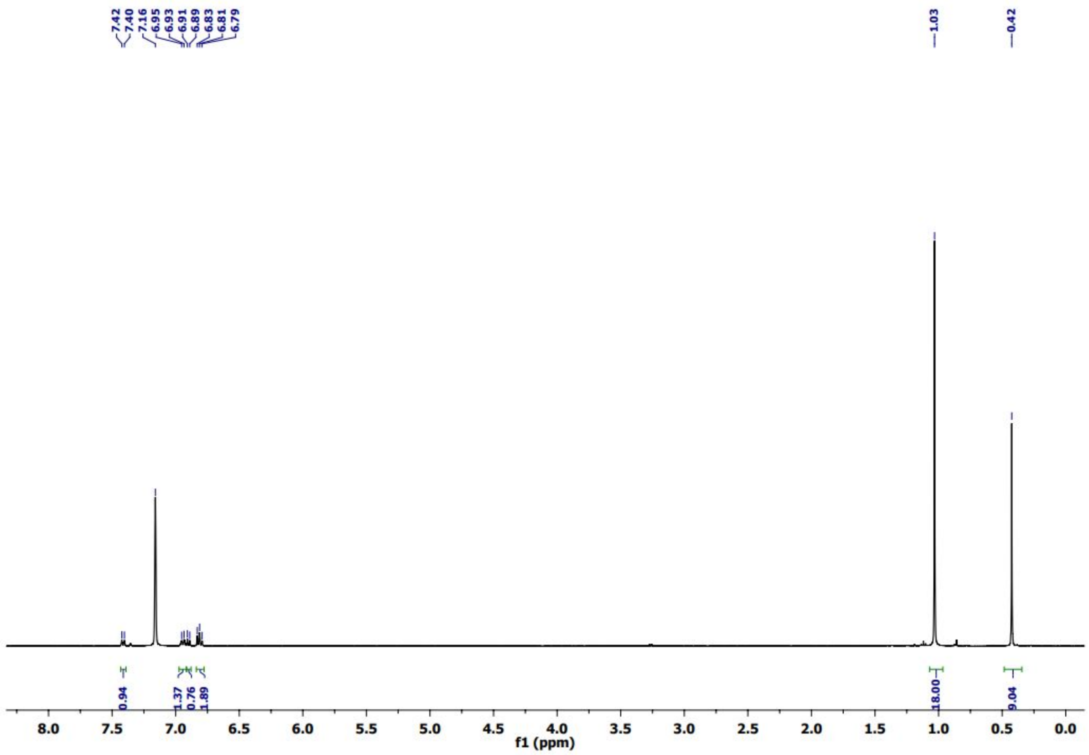

Figure S14. ^{1}H-NMR spectrum of compound **3** (400 MHz, C_6D_6, 298K)

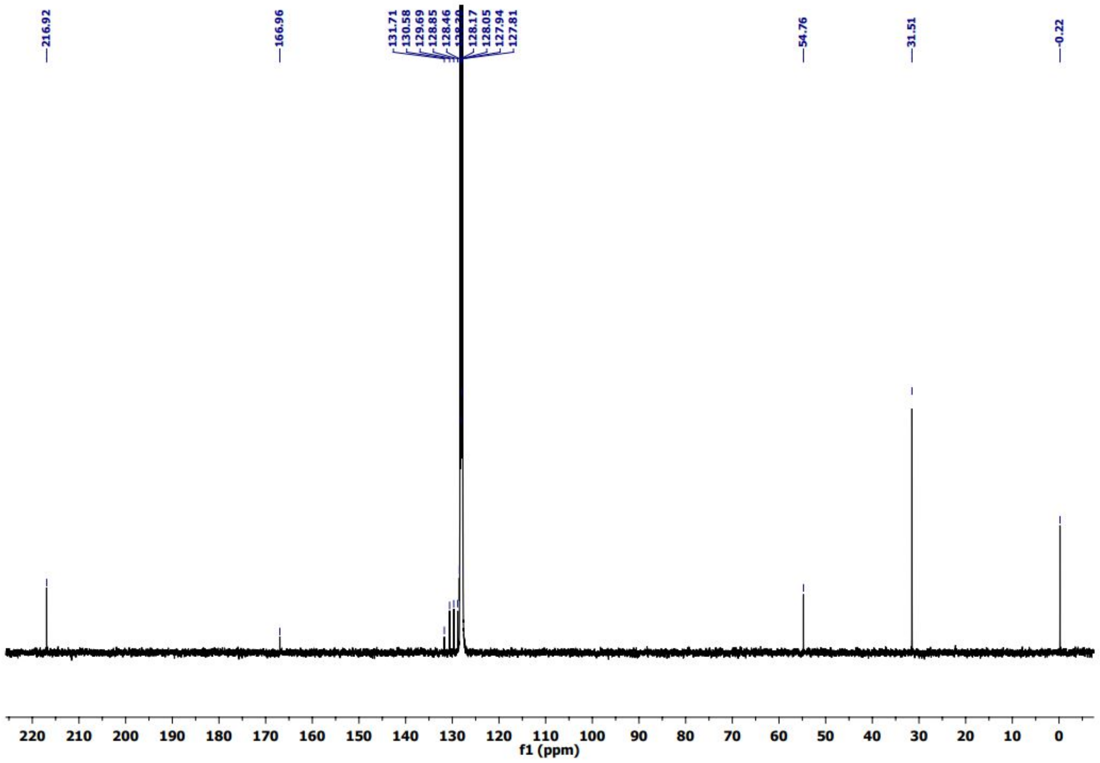

Figure S15. $^{13}C\{^1H\}$-NMR spectrum of compound **3** (125 MHz, C_6D_6, 298 K).

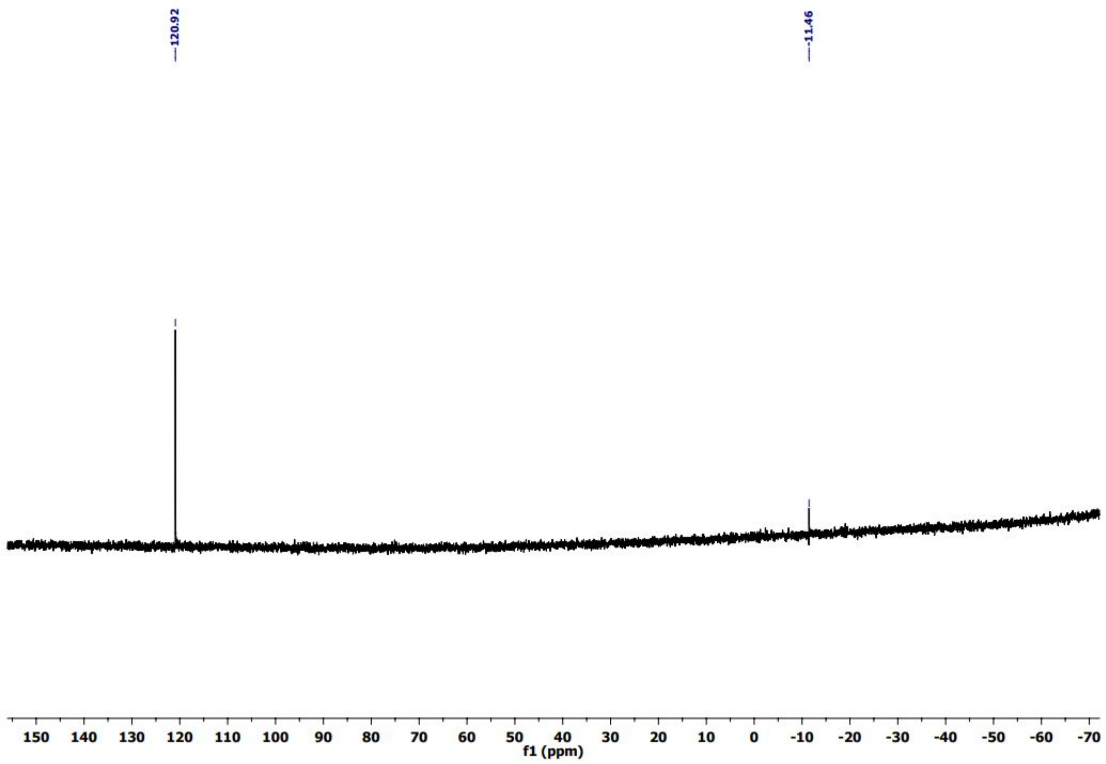

Figure S16. $^{29}Si\{^1H\}$-NMR spectrum of compound **3** (99 MHz, C_6D_6, 298 K)

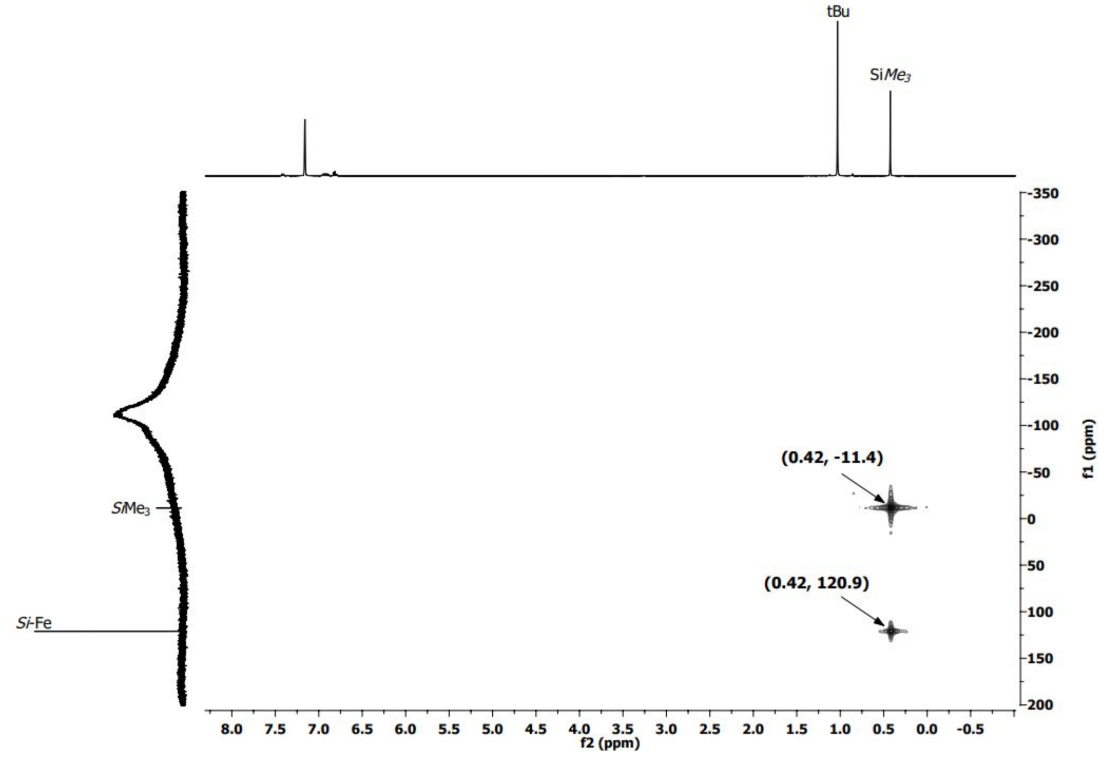

Figure S17. ^{1}H,^{29}Si-HMQC NMR Spectrum of compound **3** (400 MHz $J_{Si\text{-}H}$ = 7Hz, C_6D_6, 298 K)

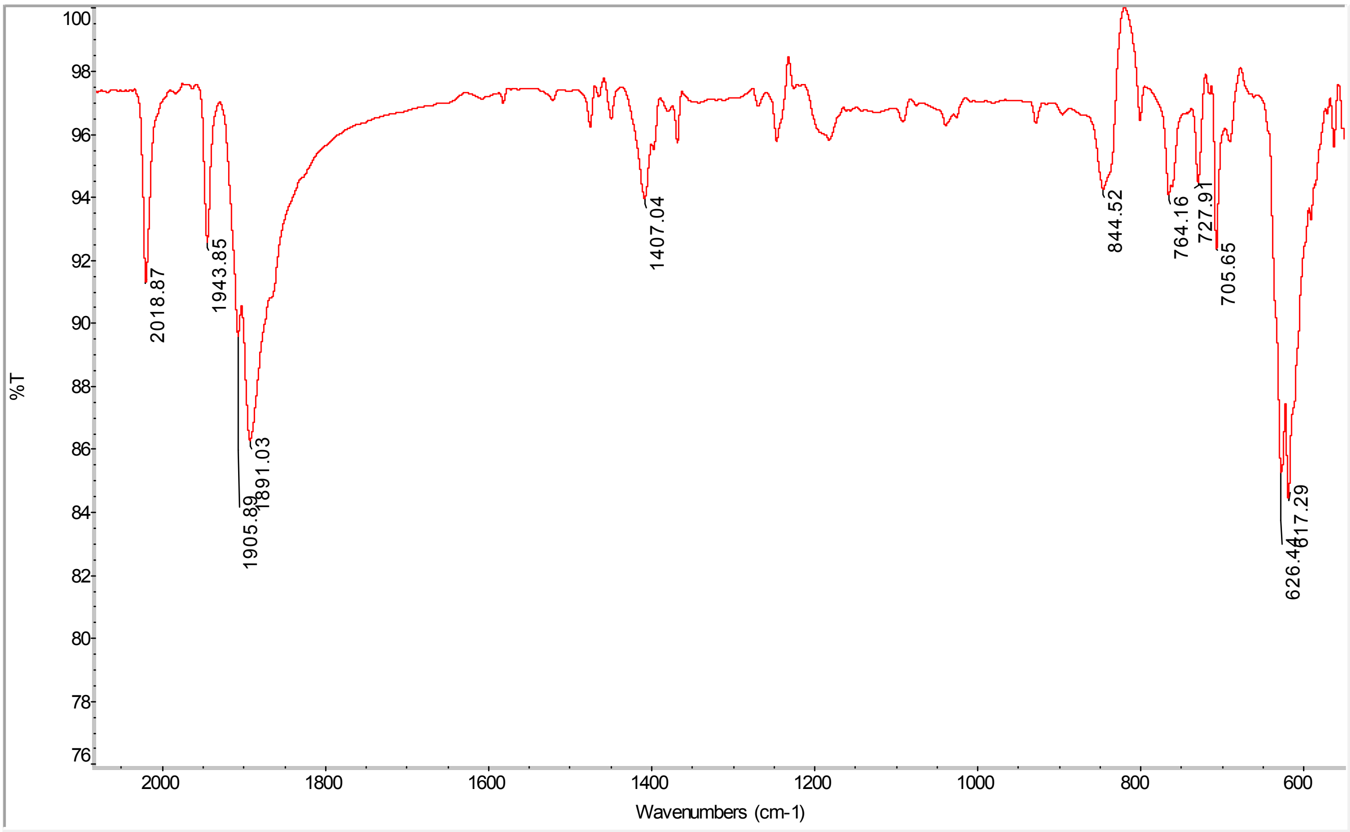

Figure S18. IR spectrum of compound **3**.

Scheme S4. Synthesis of compound **[K(2.2.2-cryptand)Bi(SiMe$_3$)(Si^{i}Pr$_3$)]**.

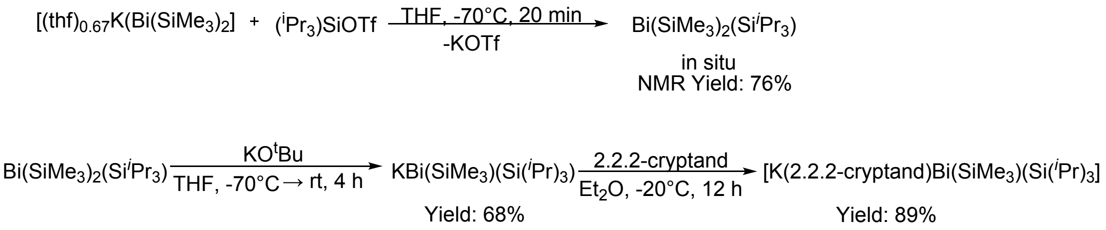

***Preparation of [K(2.2.2-cryptand)Bi(SiMe$_3$)(Si^{i}Pr$_3$)]*:** (^{i}Pr$_3$)SiOTf (2.4 g, 2.1 mL, 7.83 mmol) was dissolved in THF and stirred at -70°C to which [(thf)$_{0.67}$KBi(SiMe$_3$)$_2$] (4.5 g, 10.17 mmol) solution in THF was added dropwise over period of 5 min and the reaction was quickly monitored through NMR. The formation of Bi(SiMe$_3$)(Si^{i}Pr$_3$) in situ was clearly seen in 76% NMR yield with a period of 15 min. When almost complete consumption of starting material was seen, KO^{t}Bu (0.878 mg, 7.83 mmol) was slowly added at this temperature and the mixture was stirred for further 4 h at room temperature. After 4 h, solvent was removed and the product was extracted through toluene and washed with hexane (5 x 2 mL). The isolated green solid was used further for the reaction. Yield = 68% (2.5 g, 5.32 mmol). 2.2.2-cryptand (1.4 g, 5.32 mmol) was also dissolved in 2 mL of diethyl ether, and this solution was slowly added to [KBi(SiMe$_3$)(Si^{i}Pr$_3$)] (2.5 g, 5.32 mmol) solution in 5 mL diethyl ether at -70°C without stirring and was kept at -20°C overnight. After 12 h, greenish yellow crystals of [K(2.2.2-cryptand)Bi(SiMe$_3$)(Si^{i}Pr$_3$)] (4.0 g, 4.74 mmol) were obtained which was suitable for X-ray crystallography.

M.p. 88.6 (decomp.).

^{1}H NMR (400 MHz, C_7D_8, 298K): δ/ppm = 1.28 (s, 9 H, BiSi*Me$_3$*), 1.42-1.47 (m, 3 H, ^{i}Pr(C-*H*), 1.59 (d, 18H, ^{i}Pr-C*H$_3$*), 2.15-2.17 (m, 12H, crypt-N*CH$_2$*), 3.18-3.20 (m, 12H, crypt-*CH$_2$*), 3.30 (s, 12H, crypt-O*CH$_2$*).

^{13}C{^{1}H} NMR (125 MHz, C_7D_8, 298K): δ/ppm = 14.55 (s, BiSi*Me$_3$*), 16.94 (s, ^{i}Pr(*C*-H), 23.99 (s, iPr-*C*H$_3$), 54.36 (s, crypt-*C*H$_2$), 60.01 (s, crypt-*C*H$_2$), 70.96 (s, crypt-*C*H$_2$).

^{29}Si{^{1}H} NMR (99 MHz, C_7D_8, 298K): δ/ppm = -62.96 (*Si*Me$_3$), 31.68(*Si*(^{i}Pr)$_3$).

^{1}H,^{29}Si-HMQC NMR (400 MHz, C_7D_8, 298 K, J_{SiH} = 7 Hz, NUS 50%) (δ_H, δ_{Si})/ppm = (1.260, -62.960), (1.58, 31.667).

HR-ESI-MS: m/z: calcd for $(C_{18}H_{36}KN_2O_6)^+$ [M]$^+$: calc. 415.22 found: 415.219

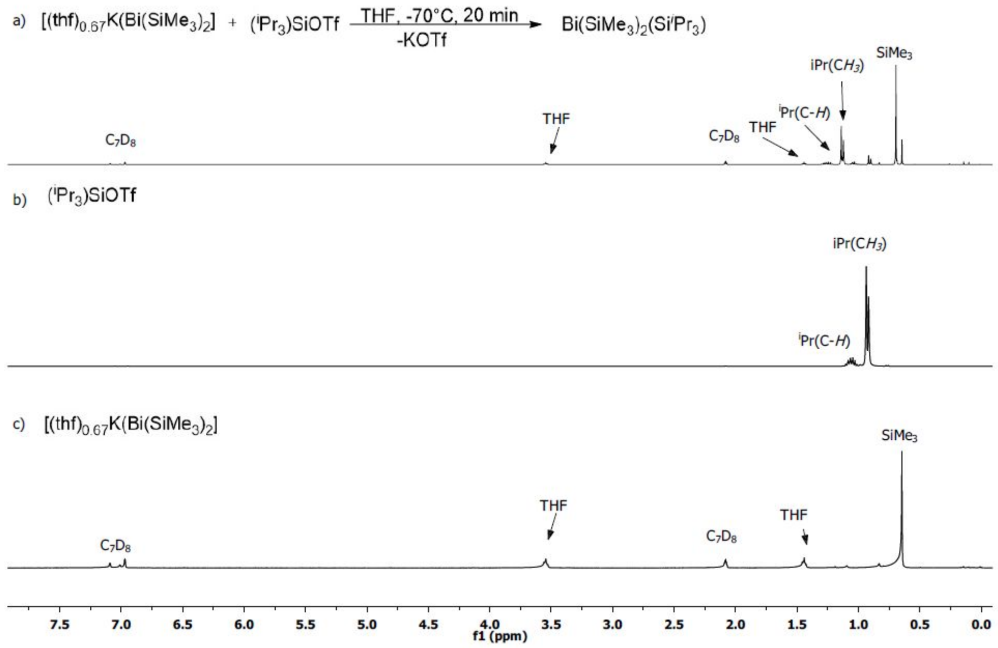

Figure S19. In-situ ^{1}H NMR Spectrum of compound $Bi(SiMe_3)_2(Si^iPr_3)$ (400 MHz C_7D_8, 298 K) Spectrum a): In situ reaction mixture of $Bi(SiMe_3)_2(Si^iPr_3)$, b) $(^iPr_3)SiOTf$ c) $[(thf)_{0.67}K(Bi(SiMe_3)_2]$.

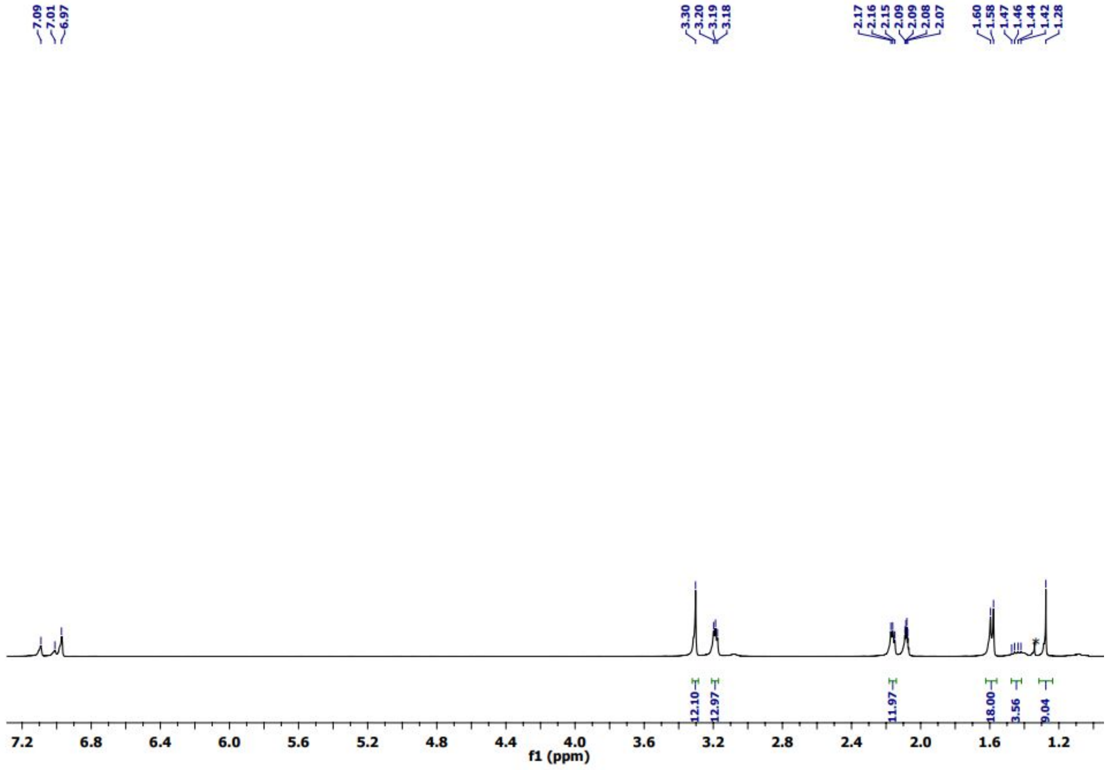

Figure S20. ^{1}H NMR Spectrum of compound [K(2.2.2-cryptand)Bi(SiMe$_3$)(Si^{i}Pr$_3$)] (400 MHz C_7D_8, 298 K)

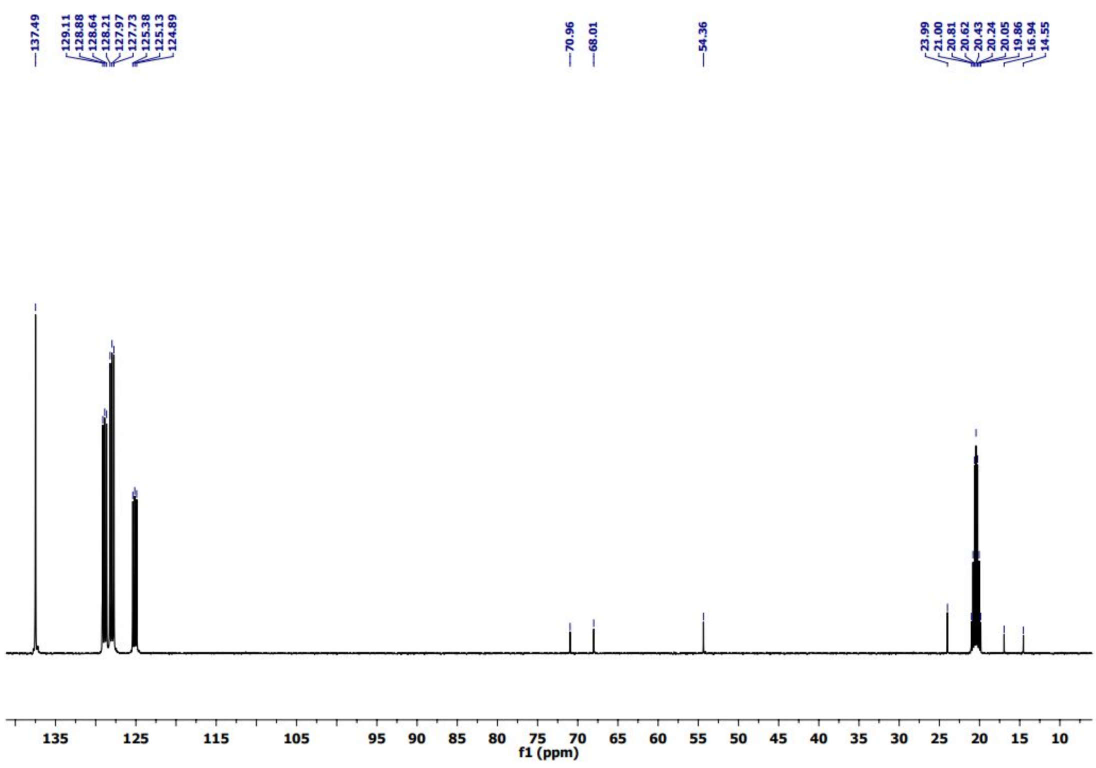

Figure S21: ^{13}C NMR Spectrum of compound [K(2.2.2-cryptand)Bi(SiMe$_3$)(Si^{i}Pr$_3$)] (400 MHz C_7D_8, 298 K)

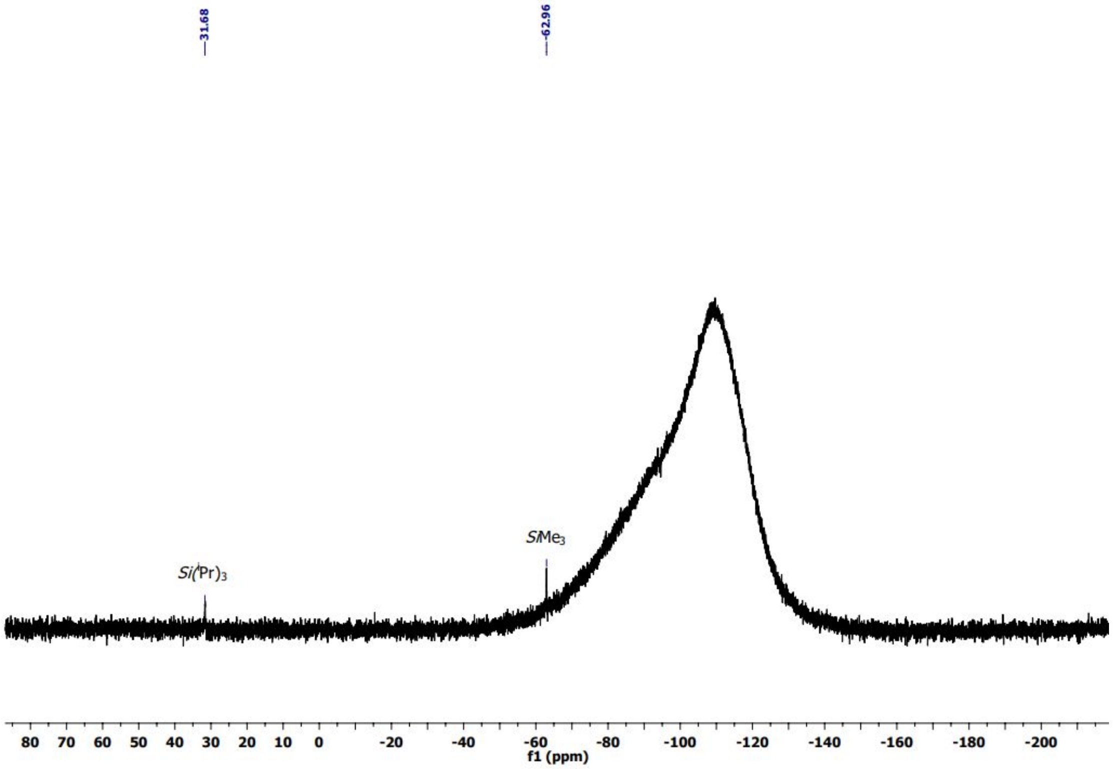

Figure S22: $^{29}Si\{H\}$ NMR Spectrum of compound [K(2.2.2-cryptand)Bi($SiMe_3$)(Si^iPr_3)] (400 MHz C_7D_8, 298 K)

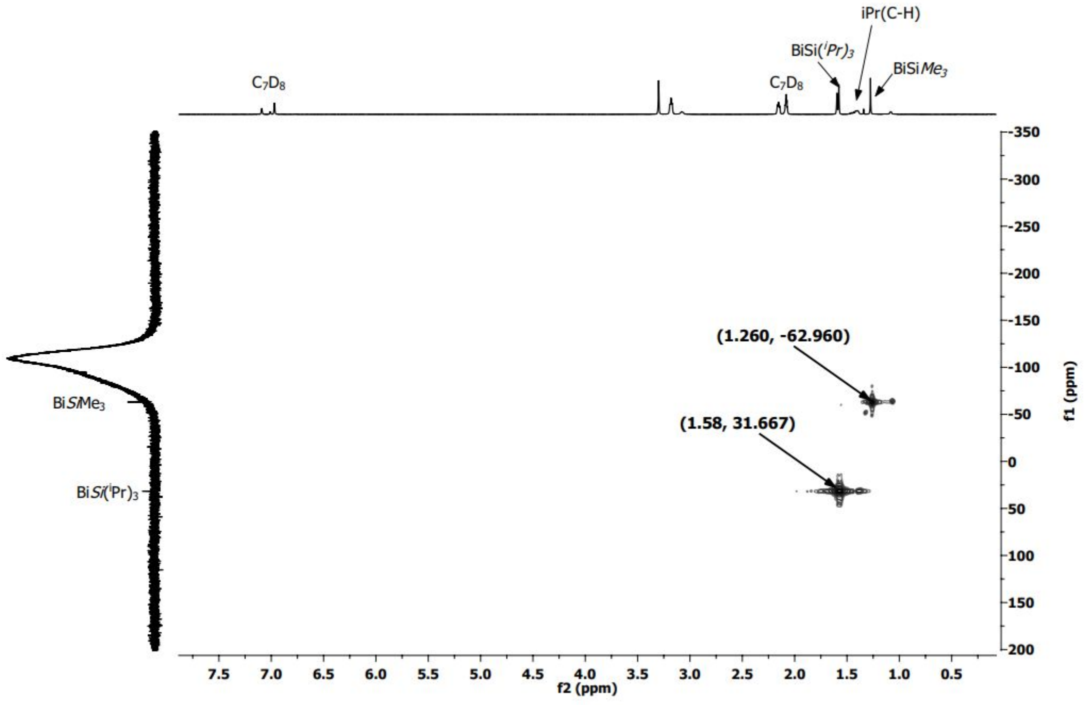

Figure S23: $^{1}H,^{29}Si$-HMQC NMR spectrum of compound [K(2.2.2cryptand)Bi($SiMe_3$)(Si^iPr_3)] (400 MHz $J_{Si\text{-}H}$ = 7Hz, C_7D_8, 298 K)

Scheme S5. Synthesis of compound ***(E)*-1b.**

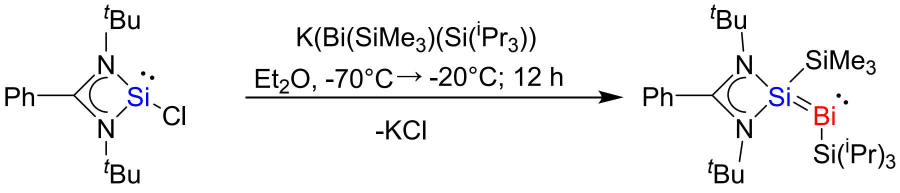

***(E)*-1b** Yeild: 60%

Synthesis of compound (E)-1b*:** In a 100 mL Schlenk flask, chlorosilylene LSiCl(1 g, 3.3 mmol) was dissolved in 10 mL of diethyl ether, and the solution was kept at -70ºC in an acetone bath. To this, a greenish-yellow solution of [KBi(SiMe$_3$)(Si(iPr)$_3$] (1.57 g, 3.3 mmol) in 10 mL of diethyl ether was added dropwise over a period of 15 min without stirring. On addition of greenish yellow solution of [KBi(SiMe$_3$)(Si(^{i}Pr)$_3$] to colourless solution of chlorosilylene, formation of dark red solution was observed. This solution was further stored at -20ºC for 14 h. The solution was then filtered, and the solvent was removed under vacuum. To the red residue, pentane was added and the solution was concentrated and kept at -20 ºC for 12 h, red needle shaped crystals of ***(E)-1b were obtained which were suitable for X-ray crystallography. The pentane solution was further decanted and the product was isolated as red solid. The decanted solution was further concentrated and stored at -20 °C, yielding a second fraction of red crystals. Yield = 60% (1.38 g, 1.98 mmol).

M.p. 85.6 ºC (decomp.).

^{1}H NMR (700 MHz, C_7D_8, 298K): δ/ppm = 0.38 (s, 9 H, SiSi*Me$_3$*), 1.16 (s, 18 H, NC(C*H*$_3$)$_3$), 1.39-1.40 (s, 21 H, BiSi*(^{i}Pr)$_3$* and C-*H* overlapping signals), 6.86-6.87 (m, 1 H, Ph-*H*), 6.93-6.95 (m, 1H, Ph-*H*), 6.98-6.99 (m, 1H, Ph-*H*), 7.05 (d, *J* = 8 Hz, *o*-Ph-H), 7.20 (m, Ph-*H*).

^{13}C{^{1}H} NMR (176 MHz, C_7D_8, 298K): δ/ppm = 0.00 (s, SiSi*Me$_3$*), 17.83 (s, ^{i}Pr(*C*-H)), 22.97 (s, *i*Pr-*C*H$_3$), 32.15 (s, C*Me$_3$)*, 55.04 (s, *C*Me$_3$), 128.26, 128.45, 129.19, 129.55, 130.21, 130.8 (Ph), 134.78 (s, N*C*N).

^{29}Si{^{1}H} NMR (139 MHz, C_7D_8, 298 K): δ/ppm = -6.81 (Si*Si*Me$_3$), 20.36 (*Si*=Bi), 44.05 (Bi*Si*Me$_3$).

^{1}H,^{29}Si-HMQC NMR (700 MHz, C_7D_8, 298 K, J_{SiH} = 7 Hz, NUS 50%) (δ_H, δ_{Si})/ppm = (1.41, -28.655), (0.42, -7.699), (0.42, 37.959).

IR (cm^{-1}): 2963.59(m), 2856.91(m), 2157.21(w), 1971.38(w), 1458.07(m), 1444.0(w), 1392.49(s), 1363.31(s), 1269.18(w), 1239.89(m), 1199.37(m), 1160.52(w), 1082.15(w), 989.33(w), 877.84(w), 831.22(s), 795.45(w), 757.13(s), 720.24(m), 707.56(m), 687.99(w).

HR-ESI-MS: (m/z): calcd for $(C_{18}H_{33}N_2Si_2)^+$ [M+H]$^+$: 333.2178 found: 333.2175. Note: The base ion peak of complex was not detected due to rapid ionization decomposition.

UV-Visible: λ_{max} = 456 nm; λ_{max} = 352, 325 nm.

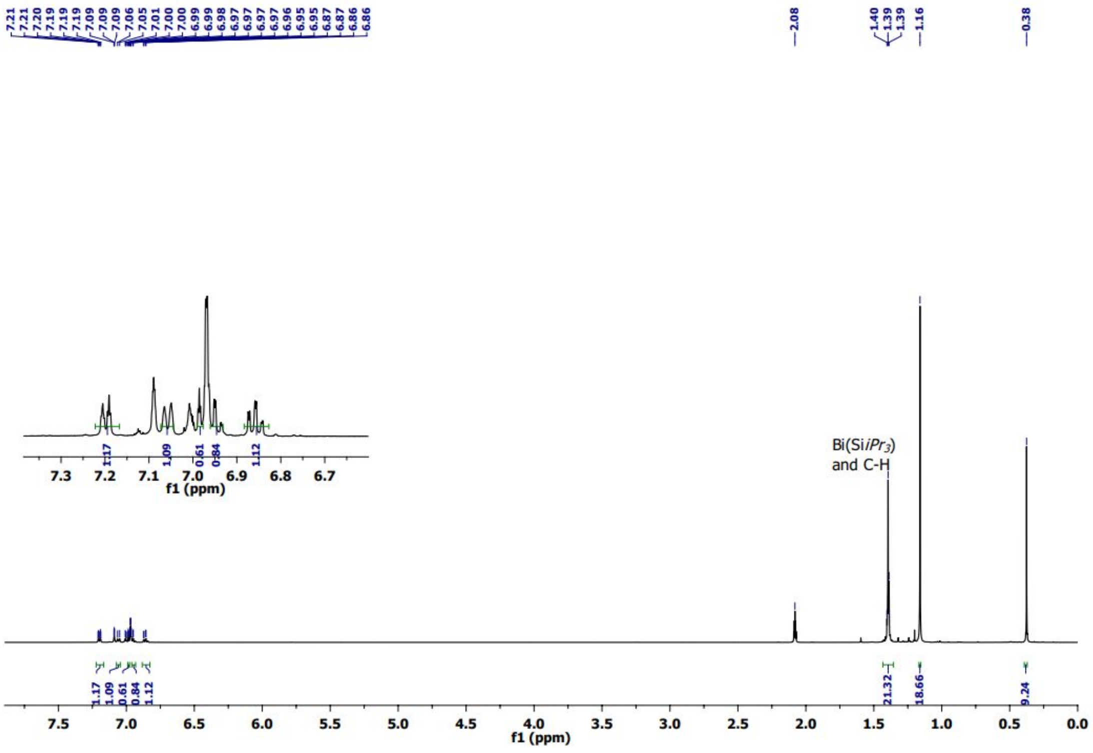

Figure S24. ^{1}H NMR Spectrum of compound ***(E)*-1b** (700 MHz C_7D_8, 298 K)

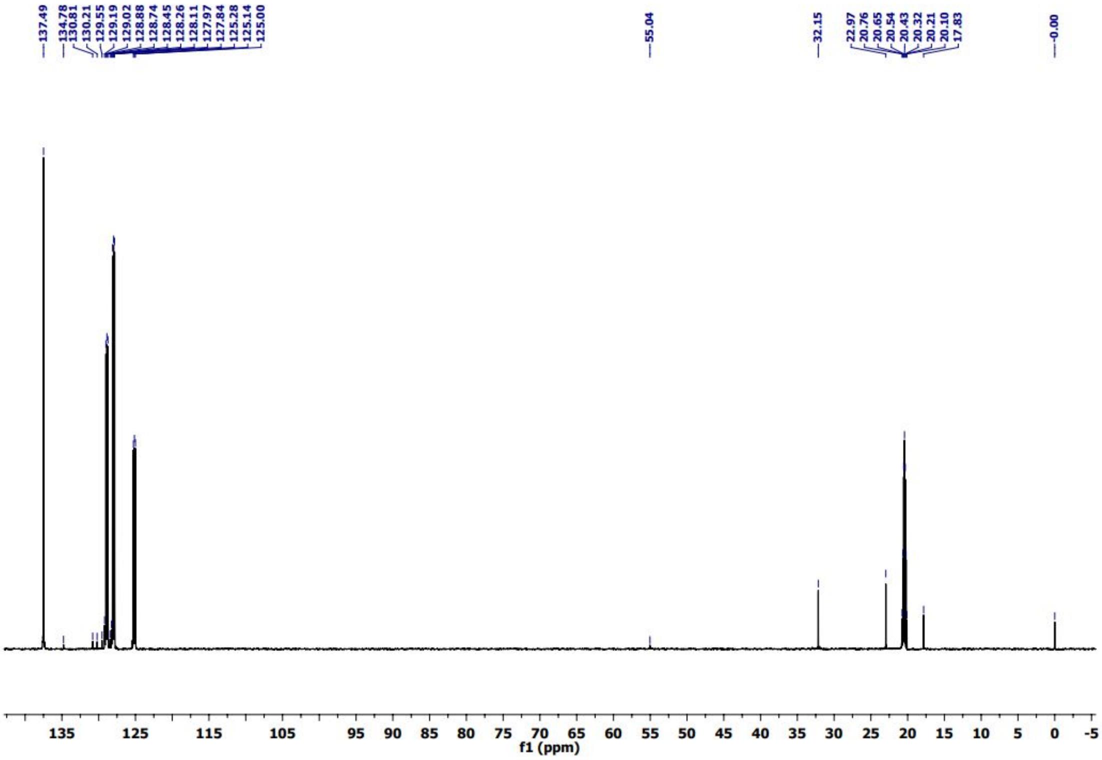

Figure S25. $^{12}C\{H\}$ NMR Spectrum of compound ***(E)*-1b** (176 MHz C_7D_8, 298 K)

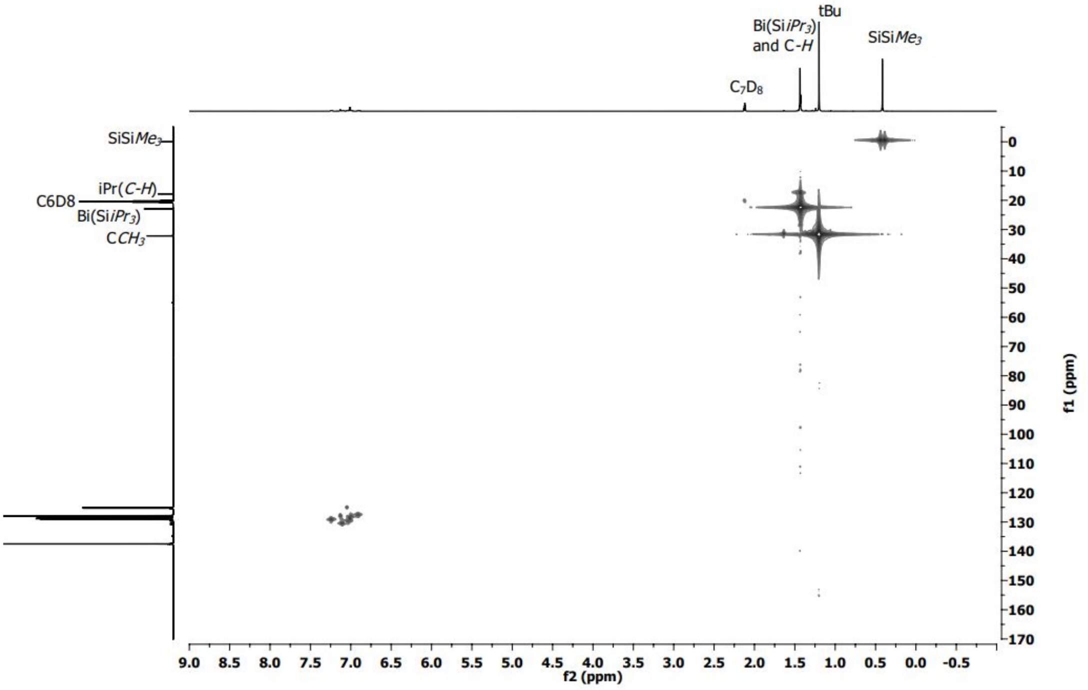

Figure S26. ^{1}H,^{13}C HMQC NMR spectrum of compound ***(E)*-1b** (700 MHz, C_7D_8, 298 K).

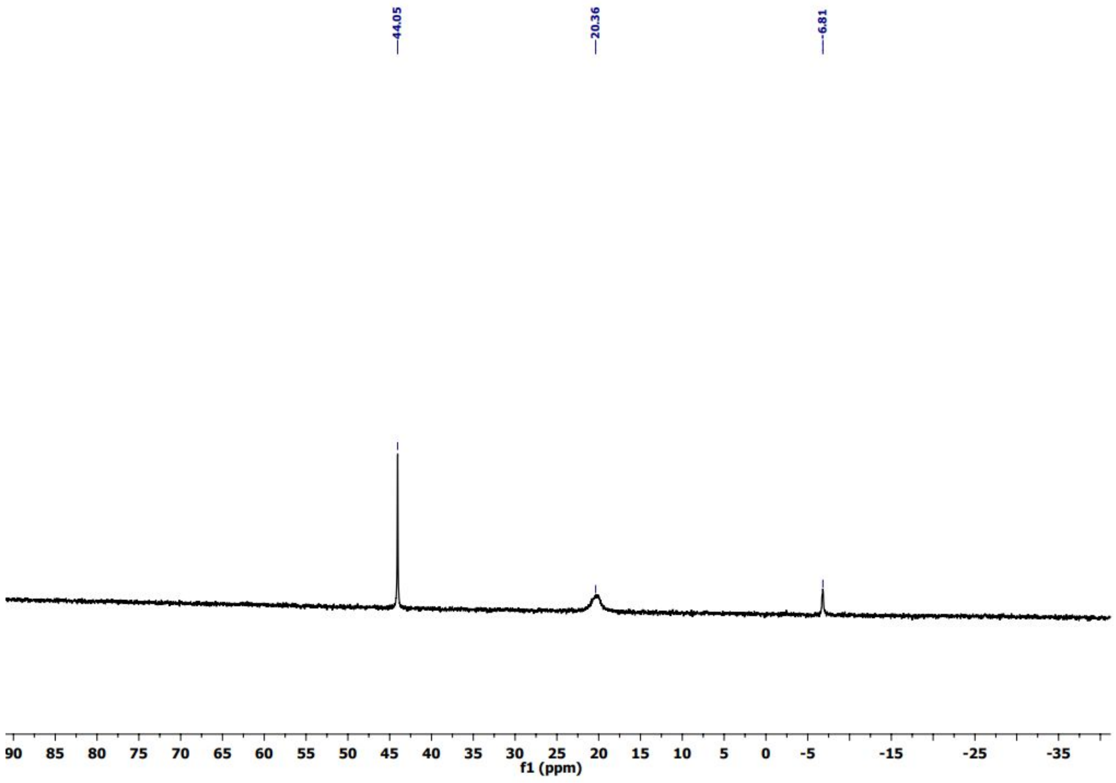

Figure S27. $^{29}Si\{H\}$ spectrum of compound ***(E)*-1b** (139 MHz, C_7D_8, 298 K).

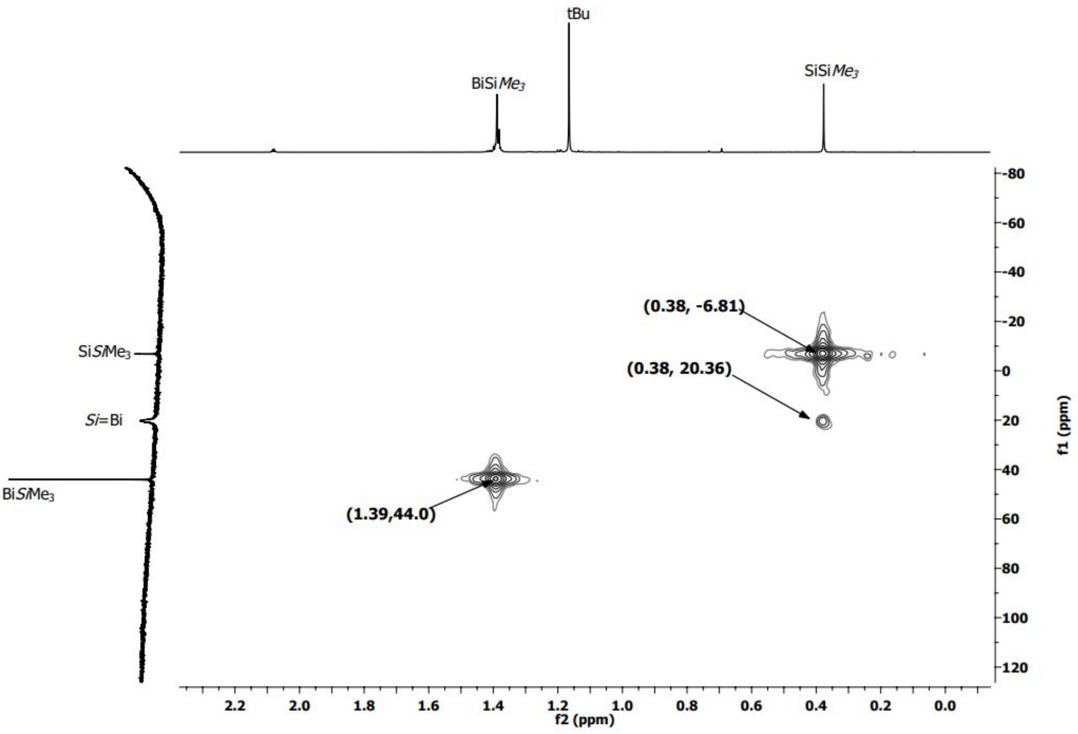

Figure S28. $^{1}H,^{13}C$ HMQC NMR spectrum of compound ***(E)*-1b** (700 MHz, C_7D_8, 298 K).

NOTE: The spectra have been zoomed in to show better correlation of peaks.

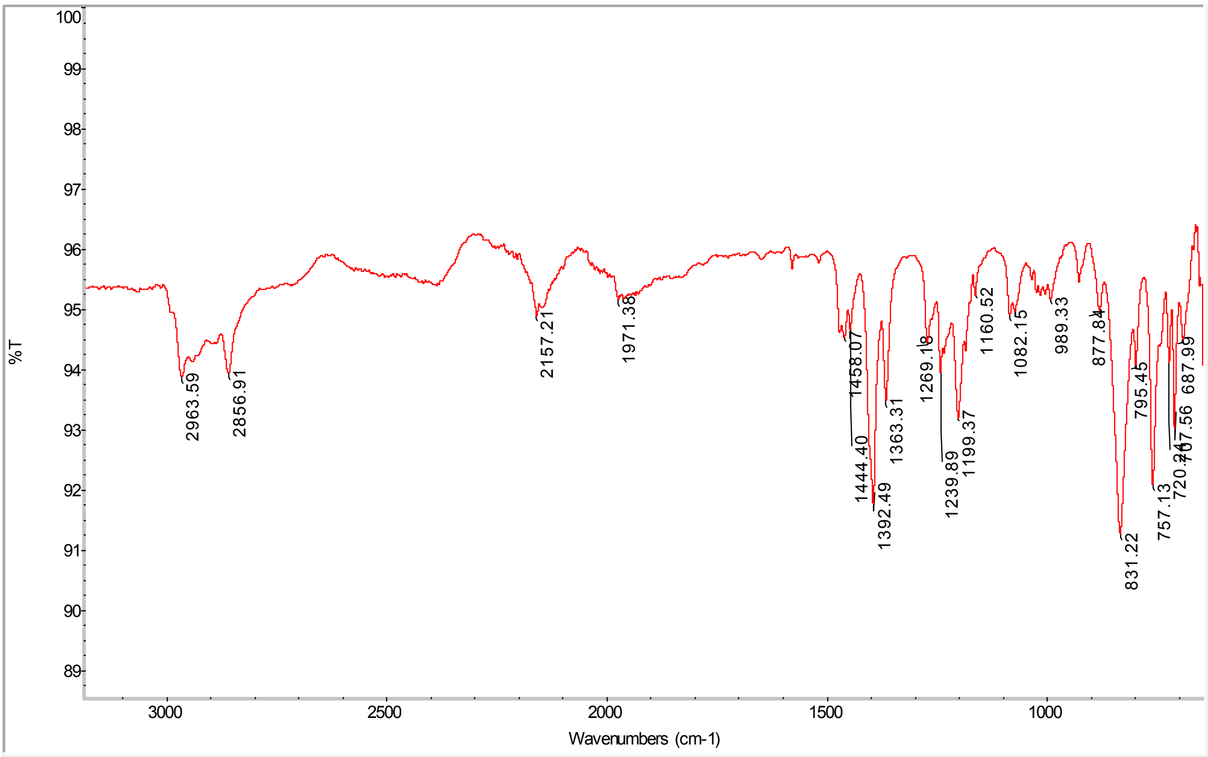

Figure S29. IR spectrum of ***(E)*-1b**

Scheme S6. Synthesis of compound ***(Z)*-2b.**

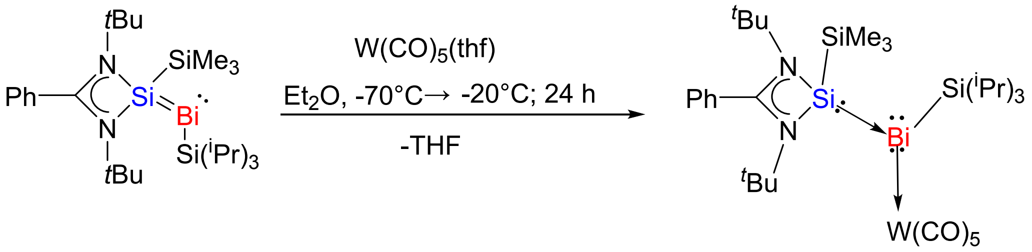

(*E*)-1b **(*Z*)-2b** Yield:47%

***Synthesis of compound (Z)-2b*:** In a 100 mL Schlenk flask [$W(CO)_6$] (114 mg, 0.324 mmol) was stirred in 5 mL of THF and irradiated with UV light (320nm-400nm) for 4 h under N_2 flow to obtain a freshly prepared yellow solution of [$W(CO)_5$(thf)] *in situ*. The freshly prepared [$W(CO)_5$(thf)] was further added to a solution of complex **(*E*)-1b** (226 mg, 0.324 mmol) in 10 mL of ether at -70ºC without stirring. The reaction mixture was then kept at -20ºC for 2 days. After 2 days, some amount of black precipitate corresponding to elemental bismuth was observed; thus, the solution was filtered, and the solvent was evacuated to obtain a brownish-black residue. This residue was washed with 4-5 times (2-5 mL) with cold pentane; the pentane solution was concentrated and kept at -20 ºC which gave orange yellow crystal suitable for X-ray crystallography. The solvent was evacuated from the remaining red solid to afford pure compound ***(Z)*-2b**. Yield = 47% (155 mg, 0.152 mmol).

M.p. 138.9 ºC (decomp.).

^{1}H NMR (500 MHz, C_7D_8, 298K): δ/ppm = 0.23 (s, 9 H, SiSi*Me₃*), 1.07 (s, 18 H, NC(CH_3)$_3$), 1.33 (d, J = 7.5 Hz, 18 H, BiSi*(iPr)*$_3$), 1.57-1.66 (m, J =7.5 Hz, iPr(C-*H*)), 6.81-6.87 (m, 1H, Ph-*H*), 6.98-6.99 (m, 2 H, Ph-*H*), 7.03-7.04 (d, J = 7.5 Hz, 1 H Ph-*H*), 7.48-7.50 (m, 1H, Ph-*H*).

^{13}C{^{1}H} NMR (125 MHz, C_7D_8, 298K): δ/ppm = -0.51 (s, SiSiMe_3), 18.88 (s, ^{i}Pr(*C*-H)), 21.55 (s, iPr-*C*H_3), 31.86 (s, CMe_3), 55.52 (s, *C*Me_3), 129.68, 130.96, 131.89, 133.40, (Ph), 168.10 (s, w, N*C*N), 203.78 (s, W(*C*O)$_5$).

^{29}Si{^{1}H} NMR (99 MHz, C_7D_8, 298 K): δ/ppm = -43.53 (*Si*=Bi), -11.22 (Si*Si*Me_3), 29.47 (Bi*Si*^{i}Pr$_3$).

^{1}H,^{29}Si-HMQC NMR (400 MHz, C_7D_8, 203 K, J_{SiH} = 7 Hz, NUS 50%) (δ_H, δ_{Si})/ppm = (0.23, -43.5), (0.23, -11.23), (1.32, 29.47).

IR (cm^{-1}): 2860.63(w), 2034.11(m), 1878.43(s), 1606.96(w), 1468.49(w), 1391.14(w), 1366.56(w), 1233.23(s), 1198.83(w), 1152.93(w), 983.50(m), 877.04(w), 834.61(m), 760.14(m), 721.92(w), 704.38(w), 628.83(m), 597.72(m), 586.13(w).

HR-ESI-MS (m/z): Repeated attempts to acquire mass data resulted in rapid decomposition of the complex under ionization conditions.

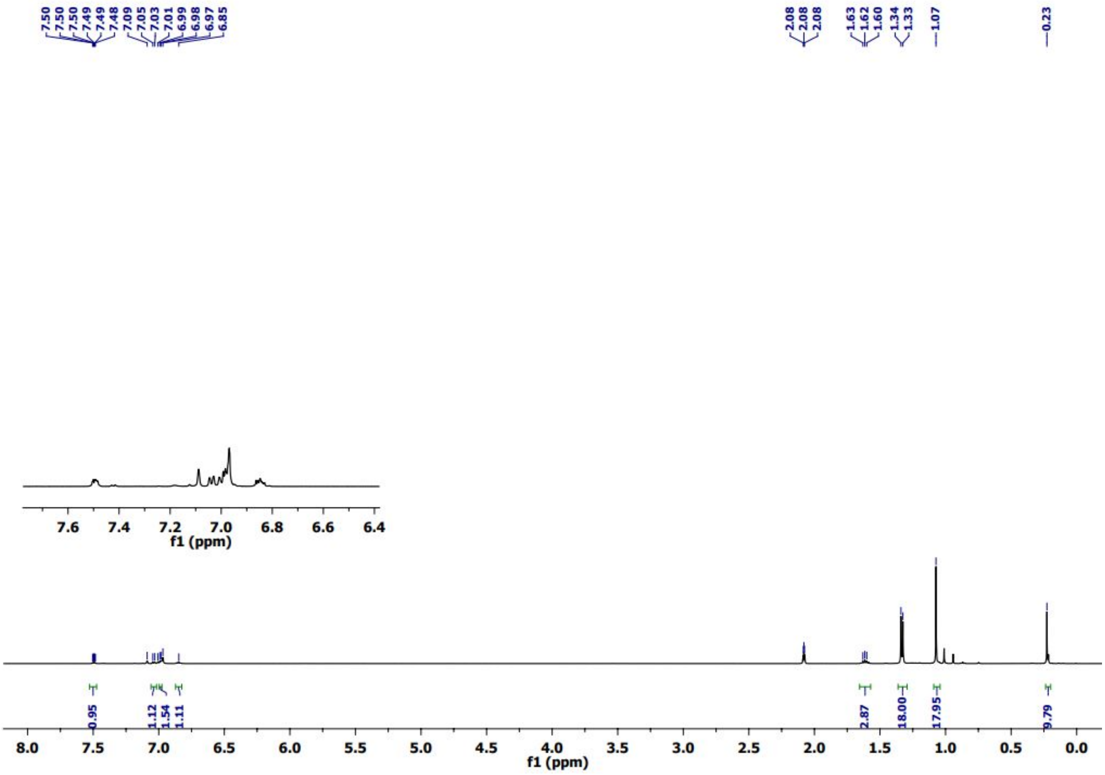

Figure S30. ^{1}H NMR Spectrum of compound ***(Z)*-2b** (500 MHz C_7D_8, 298 K)

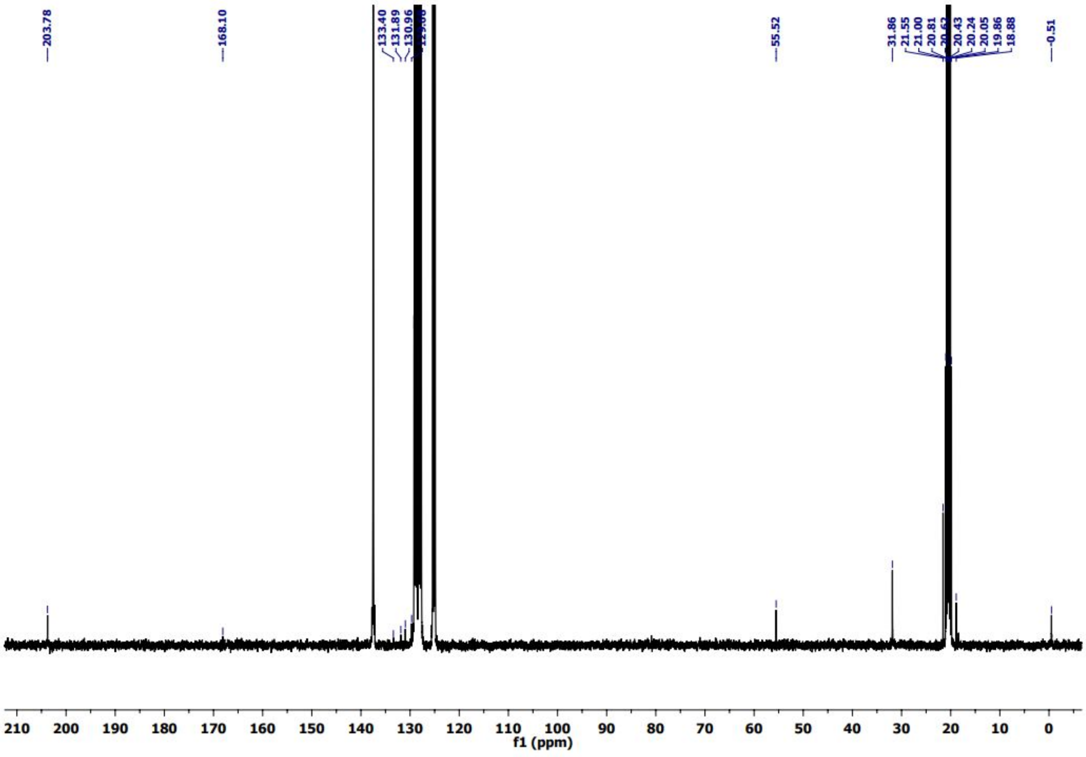

Figure S31. ^{1}H NMR Spectrum of compound ***(Z)*-2b** (125 MHz C_7D_8, 298 K)

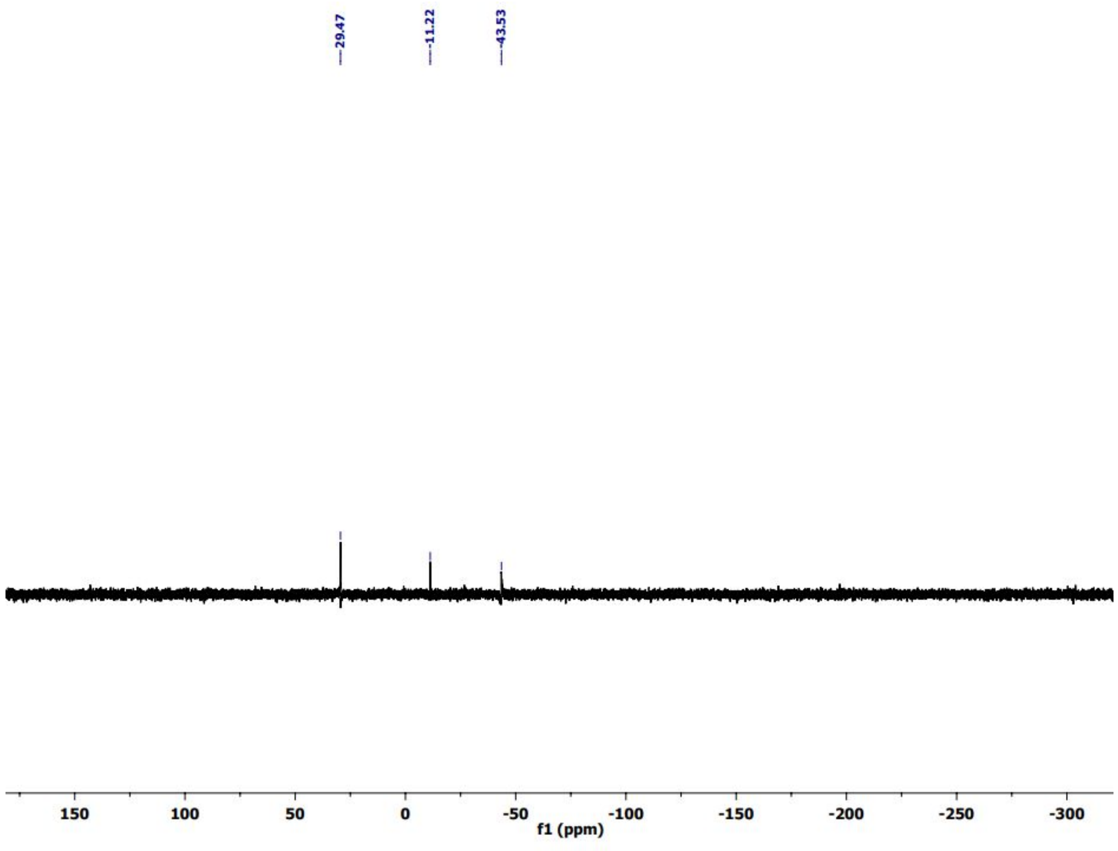

Figure S32. $^{29}Si\{H\}$ spectrum of compound ***(Z)*-2b** (99 MHz, C_7D_8, 298 K).

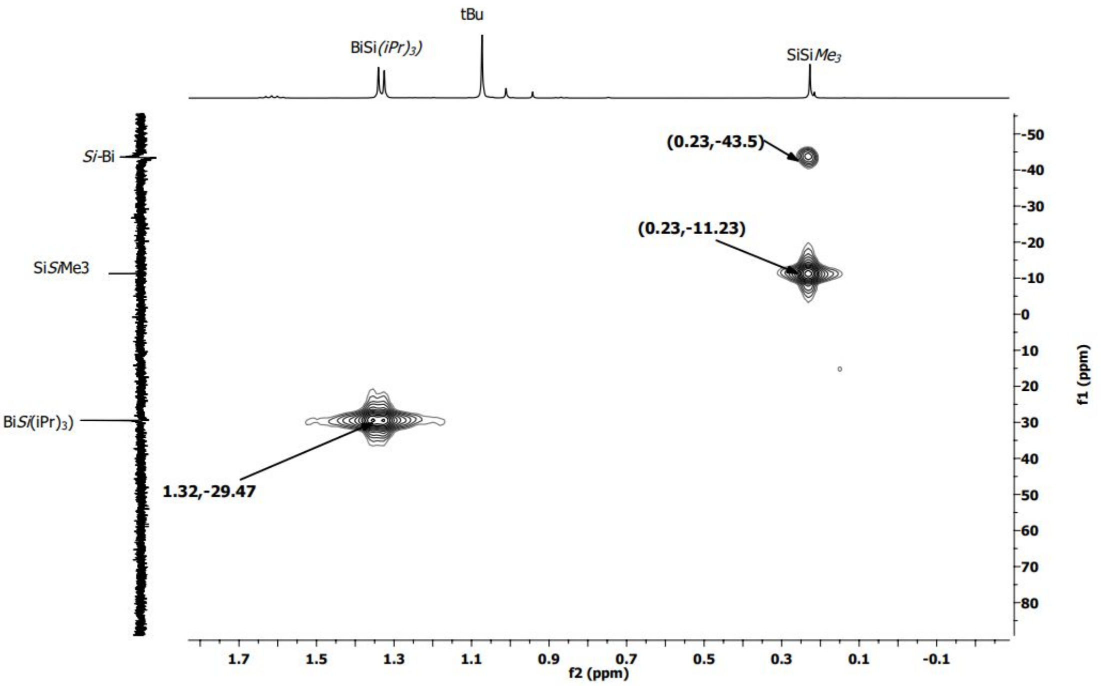

Figure S33. ^{1}H,^{29}Si HMQC NMR spectrum of compound ***(Z)*-2b** (400 MHz, C_7D_8, 298 K).

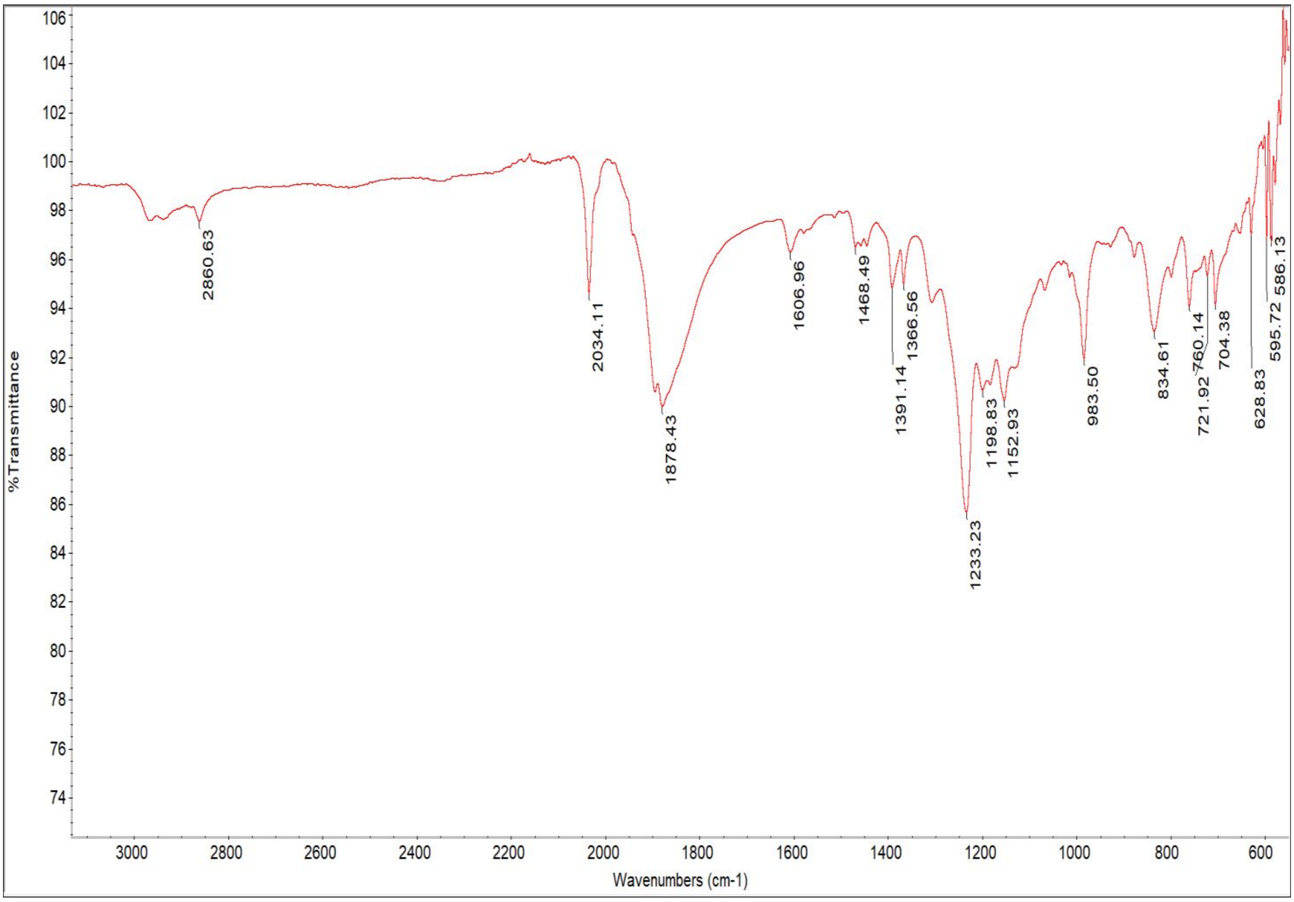

Figure S34. IR spectrum of compound ***(Z)*-2b**

A4. Crystallographic data

Table S1. Crystal data and structure refinement for ***(E)*-1a**.

Empirical formula	$C_{21}H_{41}$ Bi N_2 Si_3	
Formula weight	614.81	
Temperature	150.15 K	
Wavelength	1.54184 Å	
Crystal system	Monoclinic	
Space group	$P2_1/n$	
Unit cell dimensions	a = 9.1694(2) Å	a= 90°.
	b = 17.3149(3) Å	b= 91.7360(10)°.
	c = 17.8815(3) Å	g = 90°.
Volume	2837.69(9) $Å^3$	
Z	4	
Density (calculated)	1.439 Mg/m^3	
Absorption coefficient	13.458 mm^{-1}	
F(000)	1224	
Crystal size	0.09 x 0 x 0 mm^3	
Theta range for data collection	3.554 to 72.521°.	
Index ranges	-11<=h<=10, -18<=k<=20, -21<=l<=21	
Reflections collected	11067	
Independent reflections	5466 [$R_{(int)}$ = 0.0370]	
Completeness to theta = 67.684°	100.0 %	
Absorption correction	Semi-empirical from equivalents	
Max. and min. transmission	1.00000 and 0.18508	
Refinement method	Full-matrix least-squares on F^2	
Data / restraints / parameters	5466 / 0 / 256	
Goodness-of-fit on F^2	1.065	
Final R indices [I>2sigma(I)]	R_1 = 0.0348, wR_2 = 0.0928	
R indices (all data)	R_1 = 0.0367, wR_2 = 0.0947	
Extinction coefficient	n/a	
Largest diff. peak and hole	2.142 and -2.598 e.$Å^{-3}$	

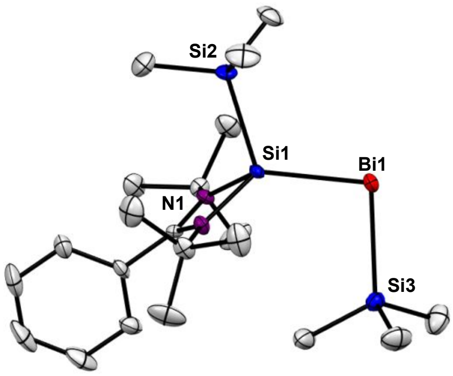

Figure S35. Molecular structure of compound ***(E)*****-1a.** Thermal ellipsoids are drawn at 50% probability level. H atoms are omitted for clarity.

Table S2. Selected interatomic distances [Å] and angles [°] for compound ***(E)*****-1a**

Bond distances [Å]		Bond angles [°]	
Bi1-Si1	2.5356(10)	Si1-Bi1-Si3	97.83(3)
Bi1-Si3	2.6370(11)	Si2-Si1-Bi1	112.48(4)
Si1-Si2	2.3505(13)	N2-Si1-Bi1	126.54(12)
Si1-N2	1.850(3)	N2-Si1-Si2	108.44(11)
Si1-N1	1.851(3)	N2-Si1-N1	71.00(14)
N2 -C7	1.337(6)	N1-Si1-Bi1	125.66(11)
		N1-Si1-Si2	105.63(11)
		C7-N2-Si1	91.0(2)
		C7-N1-Si1	91.0(2)

Table S3. Crystal data and structure refinement for ***(E)*-1b**

Empirical formula	$C_{27} H_{53} Bi N_2 Si_3$	
Formula weight	698.96	
Temperature	150.15 K	
Wavelength	1.54184 Å	
Crystal system	Monoclinic	
Space group	$P2_1/n$	
Unit cell dimensions	a = 9.2201(3) Å	a= 90°.
	b = 19.2982(6) Å	b= 101.834(3)°.
	c = 19.1679(6) Å	g = 90°.
Volume	3338.08(19) $Å^3$	
Z	4	
Density (calculated)	1.391 Mg/m^3	
Absorption coefficient	11.506 mm^{-1}	
F(000)	1416	
Crystal size	0.6 x 0.18 x 0.023 mm^3	
Theta range for data collection	3.285 to 72.561°.	
Index ranges	-11<=h<=9, -23<=k<=21, -15<=l<=23	
Reflections collected	13376	
Independent reflections	6441 [R(int) = 0.0380]	
Completeness to theta = 67.684°	99.9 %	
Absorption correction	Semi-empirical from equivalents	
Max. and min. transmission	1.00000 and 0.18552	
Refinement method	Full-matrix least-squares on F^2	
Data / restraints / parameters	6441 / 0 / 313	
Goodness-of-fit on F^2	1.028	
Final R indices [I>2sigma(I)]	R1 = 0.0301, wR2 = 0.0690	
R indices (all data)	R1 = 0.0410, wR2 = 0.0741	
Extinction coefficient	n/a	
Largest diff. peak and hole	1.260 and -1.154 e.$Å^{-3}$	

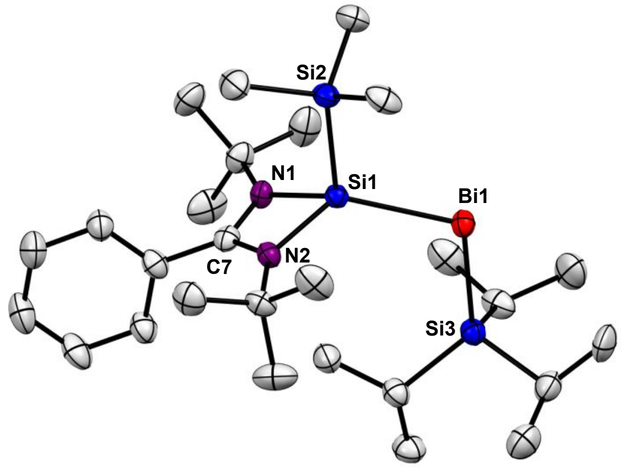

Figure S36. Molecular structure of compound ***(E)*-1b.** Thermal ellipsoids are drawn at 50% probability level. H atoms are omitted for clarity.

Table S4. Selected interatomic distances [Å] and angles [°] for compound ***(E)*-1b**

Bond distances [Å]		Bond angles [°]	
Bi1-Si1	2.5432(11)	Si1-Bi1-Si3	108.77(4)
Bi1-Si3	2.6636(11)	Si2-Si1-Bi1	109.67(5)
Si1-Si2	2.3566(14)	N2-Si1-Bi1	127.26(11)
Si1-N2	1.855(4)	N2-Si1-Si2	105.05(12)
Si1-N1	1.852(4)	N2-Si1-N1	70.59(16)
N2 -C7	1.337(6)	N1-Si1-Bi1	132.59(12)
		N1-Si1-Si2	104.81(11)
		C7-N2-Si1	91.6(3)
		C7-N1-Si1	91.5(3)

Table S5. Crystal data and structure refinement for ***(Z)*-2a**.

Empirical formula	C_{26} H_{41} Bi N_2 O_5 Si_3 W	
Formula weight	938.71	
Temperature	150.15 K	
Wavelength	1.54184 Å	
Crystal system	Triclinic	
Space group	*P*-1	
Unit cell dimensions	a = 10.1548(3) Å	a= 79.438(3)°.
	b = 12.2209(4) Å	b= 80.903(3)°.
	c = 15.4281(6) Å	g = 66.999(3)°.
Volume	1724.63(11) $Å^3$	
Z	2	
Density (calculated)	1.808 Mg/m^3	
Absorption coefficient	17.249 mm^{-1}	
F(000)	900	
Crystal size	0.02 x 0.02 x 0.015 mm^3	
Theta range for data collection	2.927 to 72.629°.	
Index ranges	-12<=h<=12, -15<=k<=14, -19<=l<=18	
Reflections collected	12651	
Independent reflections	6658 [$R_{(int)}$ = 0.0353]	
Completeness to theta = 67.684°	99.9 %	
Absorption correction	Semi-empirical from equivalents	
Max. and min. transmission	1.00000 and 0.42474	
Refinement method	Full-matrix least-squares on F^2	
Data / restraints / parameters	6658 / 0 / 355	
Goodness-of-fit on F^2	1.078	
Final R indices [I>2sigma(I)]	R_1 = 0.0368, wR_2 = 0.0942	
R indices (all data)	R_1 = 0.0392, wR_2 = 0.0961	
Extinction coefficient	n/a	
Largest diff. peak and hole	1.770 and -2.890 e.$Å^{-3}$	

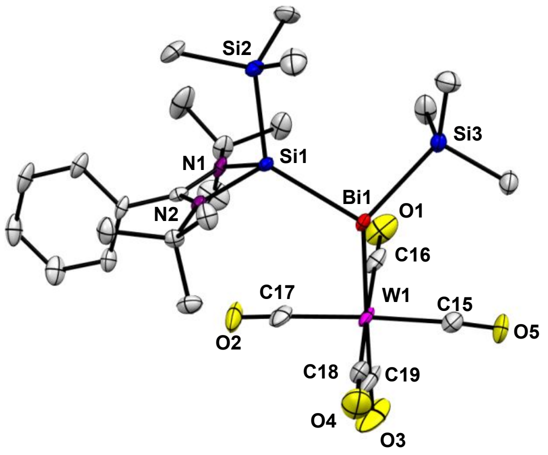

Figure S37. Molecular structure of compound ***(Z)*-2a**. Thermal ellipsoids are drawn at 50% probability level. H atoms are omitted for clarity.

Table S6. Selected interatomic distances [Å] and angles [°] for compound ***(Z)*-2a**.

Bond distances [Å]		Bond angles [°]	
Bi1-Si1	2.6172(13)	Si1-Bi1-W1	108.93(3)
Bi1-Si2	2.6690(15)	Si1-Bi1-Si2	99.30(4)
Bi1-W1	2.9559(3)	Si2-Bi1-W1	107.68(3)
Si1-Si3	2.3668(19)	Si1-Bi1-W1	108.93(3)
Si1-N2	1.830(4)	Si1-Bi1-Si2	99.30(4)
Si1-N1	1.836(5)	Si2-Bi1-W1	107.68(3)
W1-C15	2.037(7)	C15-W1-Bi1	88.48(18)
W1-C16	2.024(7)	C15-W1-C17	176.1(3)
W1-C17	2.048(7)	C15-W1-C18	86.0(3)
W1-C18	2.044(7)	C16-W1-Bi1	90.38(18)
W1-C19	1.978(7)	C16-W1-C15	91.6(3)
		C16-W1-C17	92.2(3)
		C16-W1-C18	174.5(3)
		C17-W1-Bi1	92.03(16)
		C18-W1-Bi1	84.6(2)
		C18-W1-C17	90.2(3)
		C19-W1-Bi1	176.4(2)
		C19-W1-C15	89.9(3)
		C19-W1-C16	92.8(3)

Table S7. Crystal data and structure refinement for ***(Z)*-2b**.

Empirical formula	$C_{32} H_{53} Bi N_2 O_5 Si_3 W$	
Formula weight	1022.86	
Temperature	150.15 K	
Wavelength	1.54184 Å	
Crystal system	Triclinic	
Space group	*P*-1	
Unit cell dimensions	a = 10.7832(7) Å	a= 71.847(8)°.
	b = 12.3754(9) Å	b= 75.010(7)°.
	c = 17.6946(17) Å	g = 64.594(7)°.
Volume	2005.2(3) $Å^3$	
Z	2	
Density (calculated)	1.694 Mg/m^3	
Absorption coefficient	14.890 mm^{-1}	
F(000)	996	
Crystal size	0.09 x 0.04 x 0.03 mm^3	
Theta range for data collection	2.656 to 72.676°.	
Index ranges	-12<=h<=13, -11<=k<=15, -20<=l<=21	
Reflections collected	13941	
Independent reflections	7700 [R(int) = 0.0695]	
Completeness to theta = 67.684°	99.7 %	
Absorption correction	Semi-empirical from equivalents	
Max. and min. transmission	1.00000 and 0.11658	
Refinement method	Full-matrix least-squares on F^2	
Data / restraints / parameters	7700 / 449 / 675	
Goodness-of-fit on F^2	1.011	
Final R indices [I>2sigma(I)]	R1 = 0.0492, wR2 = 0.1142	
R indices (all data)	R1 = 0.0727, wR2 = 0.1322	
Extinction coefficient	n/a	
Largest diff. peak and hole	1.867 and -1.721 e.$Å^{-3}$	

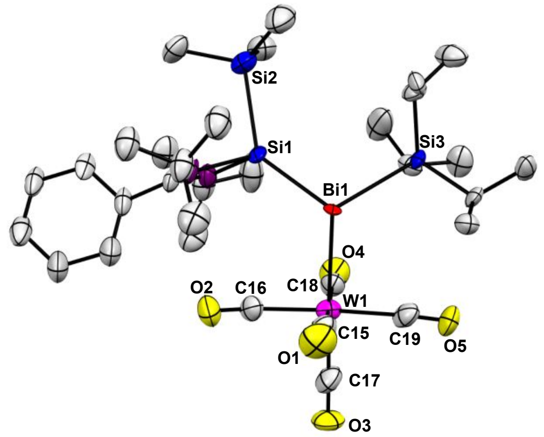

Figure S38. Molecular structure of compound ***(Z)*-2b**. Thermal ellipsoids are drawn at 50% probability level. H atoms are omitted for clarity. The most of the atoms of the molecule are disordered over two orientation Part 1 and Part 1B with an occupancy ratio 50:49

Table S8. Selected interatomic distances [Å] and angles [°] for compound ***(Z)*-2b**.

Bond distances [Å]			Bond angles [°]		
	Part 1	Part B		Part 1	Part 1B
Si1-Bi1	2.63(2)	2.641(10)	Si2-Si1-Bi1	118.8(2)	134.8(8)
Si(1)-Si(2)	2.31(2)	2.52(2)	Si3-Bi1-Si1	108.5(6)	102.8(6)
Bi(1)-Si(3)	2.58(2)	2.79(2)	Si1-Bi1-W1	108.8(5)	111.6
Bi(1)-W(1)	3.089(5)	2.817(6)	-	-	-

Table S9. Crystal data and structure refinement **3**.

Empirical formula	$C_{22} H_{32} Fe N_2 O_4 Si_2$	
Formula weight	500.52	
Temperature	150.15 K	
Wavelength	1.54184 Å	
Crystal system	Monoclinic	
Space group	$P2_1/n$	
Unit cell dimensions	a = 8.7808(2) Å	a= 90°.
	b = 16.1967(3) Å	b= 90.815(2)°.
	c = 18.0625(3) Å	g = 90°.
Volume	2568.59(9) Å^3	
Z	4	
Density (calculated)	1.294 Mg/m^3	
Absorption coefficient	5.840 mm^{-1}	
F(000)	1056	
Crystal size	0.6 x 0.23 x 0.18 mm^3	
Theta range for data collection	3.666 to 72.486°.	
Index ranges	-10<=h<=10, -19<=k<=16, -22<=l<=22	
Reflections collected	18711	
Independent reflections	5035 [$R_{(int)}$ = 0.0355]	
Completeness to theta = 67.684°	100.0 %	
Absorption correction	Semi-empirical from equivalents	
Max. and min. transmission	1.00000 and 0.37787	
Refinement method	Full-matrix least-squares on F^2	
Data / restraints / parameters	5035 / 0 / 289	
Goodness-of-fit on F^2	1.015	
Final R indices [I>2sigma(I)]	R_1 = 0.0358, wR_2 = 0.0895	
R indices (all data)	R_1 = 0.0452, wR_2 = 0.0954	
Extinction coefficient	n/a	
Largest diff. peak and hole	0.698 and -0.299 e.Å^{-3}	

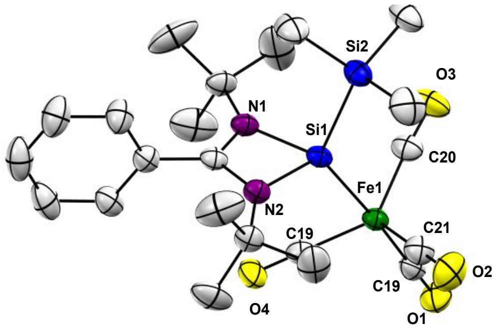

Figure S39. Molecular structure of compound **3**. Thermal ellipsoids are drawn at a 50% probability level. H atoms are omitted for clarity.

Table S10. Selected interatomic distances [Å] and angles [°] for compound **3**.

Bond distances [Å]		Bond angles [°]	
Fe1-Si1	2.2658(6)	Fe1-Si1-Si2	126.40(3)
Fe1-C19	1.773(2)	Fe1-Si1-Si2	126.40(3)
Fe1-C20	1.772(2)	N1-Si1-Fe1	118.53(6)
Fe1-C21	1.790(2)	C19-Fe1-Si1	91.38(8)
Fe1-C22	1.782(2)	C20-Fe1-Si1	87.40(8)
Si1-Si2	2.3677(7)	C21-Fe1-Si1	173.06(7)
Si1-N1	1.8390(18)	C22-Fe1-Si1	81.51(7)
Si1-N2	1.8417(18)		
N1-C1	1.339(3)		
N2-C1	1.342(3)		

Table S11. Crystal data and structure refinement for [K(2.2.2-cryptand)Bi($SiMe_3$)$_2$].

Empirical formula	$C_{24} H_{54} Bi K N_2 O_6 Si_2$	
Formula weight	770.95	
Temperature	150.15 K	
Wavelength	1.54184 Å	
Crystal system	Triclinic	
Space group	*P*-1	
Unit cell dimensions	a = 9.2014(5) Å	a= 86.553(3)°.
	b = 13.5875(6) Å	b= 83.697(4)°.
	c = 14.5062(5) Å	g = 78.329(4)°.
Volume	1764.05(14) Å^3	
Z	2	
Density (calculated)	1.451 Mg/m^3	
Absorption coefficient	11.791 mm^{-1}	
F(000)	780	
Crystal size	0.34 x 0.13 x 0.09 mm^3	
Theta range for data collection	3.067 to 72.736°.	
Index ranges	-11<=h<=11, -15<=k<=16, -17<=l<=13	
Reflections collected	12473	
Independent reflections	6809 [$R_{(int)}$ = 0.0466]	
Completeness to theta = 67.684°	99.8 %	
Absorption correction	Semi-empirical from equivalents	
Max. and min. transmission	1.00000 and 0.16325	
Refinement method	Full-matrix least-squares on F^2	
Data / restraints / parameters	6809 / 0 / 331	
Goodness-of-fit on F^2	1.034	
Final R indices [I>2sigma(I)]	R_1 = 0.0365, wR_2 = 0.0918	
R indices (all data)	R_1 = 0.0406, wR_2 = 0.0955	
Extinction coefficient	n/a	
Largest diff. peak and hole	1.274 and -2.721 e.Å^{-3}	

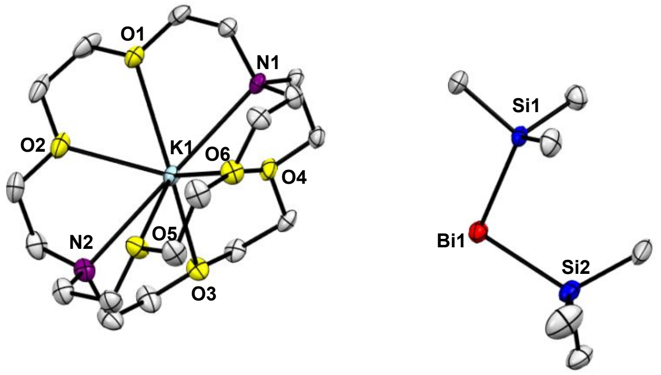

Figure S40. Molecular structure of [K(2.2.2-cryptand)Bi($SiMe_3$)$_2$]. Thermal ellipsoids are drawn at a 70% probability level. H atoms are omitted for clarity.

Table S12. Selected interatomic distances [Å] and angles [°] for [K(2.2.2-cryptand)Bi($SiMe_3$)$_2$].

Bond distances [Å]		Bond angles [°]	
Bi1-Si1	2.6129(12)	Si2-Bi1-Si1	92.18(4)
Bi1-Si2	2.6088(12)	O2-K1-O3	99.28(10)
K1-O1	2.863(3)	O4-K1-O1	100.41(10)
K1-N1	2.955(4)	O5-K1-O1	119.27(10)
K1-O2	2.807(3)	O5-K1-N1	118.46(10)
K1-N2	2.990(4)	O6-K1-N1	60.03(10)
K1-O3	2.906(3)	O6-K1-N2	119.02(10)
K1-O4	2.796(3)		
K1-O5	2.801(3)		
K1-O6	2.909(3)		

Table S13. Crystal data and structure refinement for [K(2.2.2-cryptand)Bi($SiMe_3$)(Si^iPr_3)].

Empirical formula	C_{15} H_{33} $Bi_{0.50}$ $K_{0.50}$ N O_3 Si	
Formula weight	427.55	
Temperature	150.15 K	
Wavelength	1.54184 Å	
Crystal system	Triclinic	
Space group	*P*-1	
Unit cell dimensions	a = 9.4906(2) Å	a= 89.301(2)°.
	b = 14.2594(3) Å	b= 73.114(2)°.
	c = 15.5614(4) Å	g = 89.751(2)°.
Volume	2014.98(8) $Å^3$	
Z	4	
Density (calculated)	1.409 Mg/m^3	
Absorption coefficient	10.377 mm^{-1}	
F(000)	876	
Crystal size	0.13 x 0.06 x 0.05 mm^3	
Theta range for data collection	2.968 to 72.562°.	
Index ranges	-11<=h<=11, -17<=k<=17, -19<=l<=19	
Reflections collected	15100	
Independent reflections	7774 [R(int) = 0.1471]	
Completeness to theta = 67.684°	99.9 %	
Absorption correction	Semi-empirical from equivalents	
Max. and min. transmission	1.00000 and 0.57341	
Refinement method	Full-matrix least-squares on F^2	
Data / restraints / parameters	7774 / 78 / 396	
Goodness-of-fit on F^2	1.109	
Final R indices [I>2sigma(I)]	R_1 = 0.0809, wR_2 = 0.2354	
R indices (all data)	R_1 = 0.0820, wR_2 = 0.2371	
Extinction coefficient	n/a	
Largest diff. peak and hole	3.169 and -5.612 e.$Å^{-3}$	

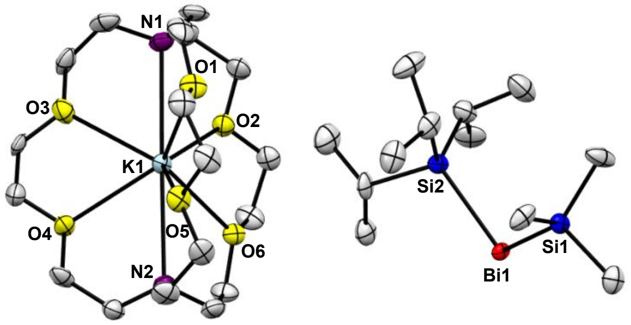

Figure S41. Molecular structure of [K(2.2.2-cryptand)Bi($SiMe_3$)(Si^iPr_3)]. Thermal ellipsoids are drawn at a 50% probability level. H atoms are omitted for clarity.

Table S14 Selected interatomic distances [Å] and angles [°] for

[K(2.2.2-cryptand)Bi($SiMe_3$) (Si^iPr_3)].

Bond distances [Å]		Bond angles [°]	
Bi1-Si1	2.597(2)	Si1-Bi1-Si2	98.47(8)
Bi1-Si2	2.619(3)	O2-K1-O5	60.0(2)
K1-O1	2.820(7)	O1-K1-O2	101.2(2)
K1-N1	3.027(9)	O2-K1-N1	60.8(2)
K1-O2	2.863(7)	O1-K1-N2	118.46(10)
K1-N2	3.019(8)	N2-K1-N1	178.9(3)
K1-O3	2.782(8)	O3-K1-N1	95.2(2)
K1-O4	2.824(7)		
K1-O5	2.893(7)		
K1-O6	2.909(3)		

Table S15 Crystal data and structure refinement for $[Bi_4(Si^iPr_3)_4]$.

Empirical formula	C_{36} H_{84} Bi_4 Si_4	
Formula weight	1465.31	
Temperature	150.15 K	
Wavelength	1.54184 Å	
Crystal system	Monoclinic	
Space group	*C*2/*c*	
Unit cell dimensions	a = 19.3867(4) Å	a= 90°.
	b = 13.6930(3) Å	b= 94.153(2)°.
	c = 18.7503(4) Å	g = 90°.
Volume	4964.42(18) $Å^3$	
Z	4	
Density (calculated)	1.961 Mg/m^3	
Absorption coefficient	28.392 mm^{-1}	
F(000)	2752	
Crystal size	0.14 x 0.07 x 0.06 mm^3	
Theta range for data collection	3.956 to 72.453°.	
Index ranges	-21<=h<=23, -16<=k<=13, -22<=l<=18	
Reflections collected	9697	
Independent reflections	4783 [R(int) = 0.0466]	
Completeness to theta = 67.684°	99.8 %	
Absorption correction	Semi-empirical from equivalents	
Max. and min. transmission	1.00000 and 0.13761	
Refinement method	Full-matrix least-squares on F^2	
Data / restraints / parameters	4783 / 169 / 241	
Goodness-of-fit on F^2	1.033	
Final R indices [I>2sigma(I)]	R_1 = 0.0455, wR_2 = 0.1245	
R indices (all data)	R_1 = 0.0523, wR_2 = 0.1331	
Extinction coefficient	n/a	
Largest diff. peak and hole	4.004 and -2.827 e.$Å^{-3}$	

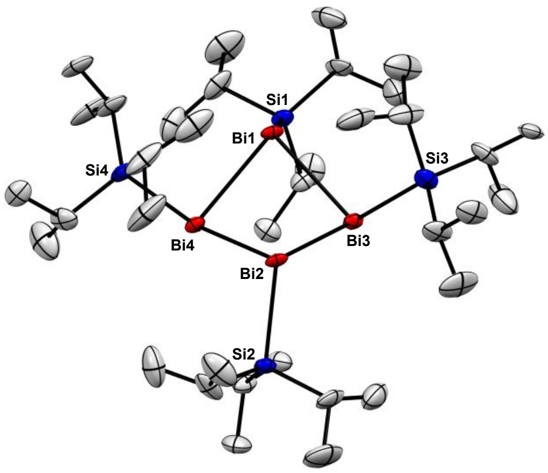

Figure S42. Molecular structure of [$Bi_4(Si^iPr_3)_4$]. Thermal ellipsoids are drawn at a 50% probability level. H atoms are omitted for clarity. The molecule is disordered over two different orientations, which was successfully refined.

Table S16. Selected interatomic distances [Å] and angles [°] for [$Bi_4(Si^iPr_3)_4$].

Bond distances [Å]		Bond angles [°]	
Bi1-Si1	2.694(2)	Bi3-Bi1-Bi4	81.939(12)
Bi2-Bi3	3.0158(4)	Bi3-Bi2-Bi4	81.985(12)
Bi1-Bi3	3.0117(4)	Si1-Bi1-Bi3	95.88(5)
Bi1-Bi4	3.0158(4)	Si1-Bi1-Bi4	105.14(5)
Bi2-Bi4	3.0118(4)	Si2-Bi2-Bi4	96.49(6)
		Si2-Bi2-Bi3	102.18(6)

NOTE: The residual electron density peaks are standard Fourier ripples due to heavy bismuth atoms.

A5. UV-Visible spectra

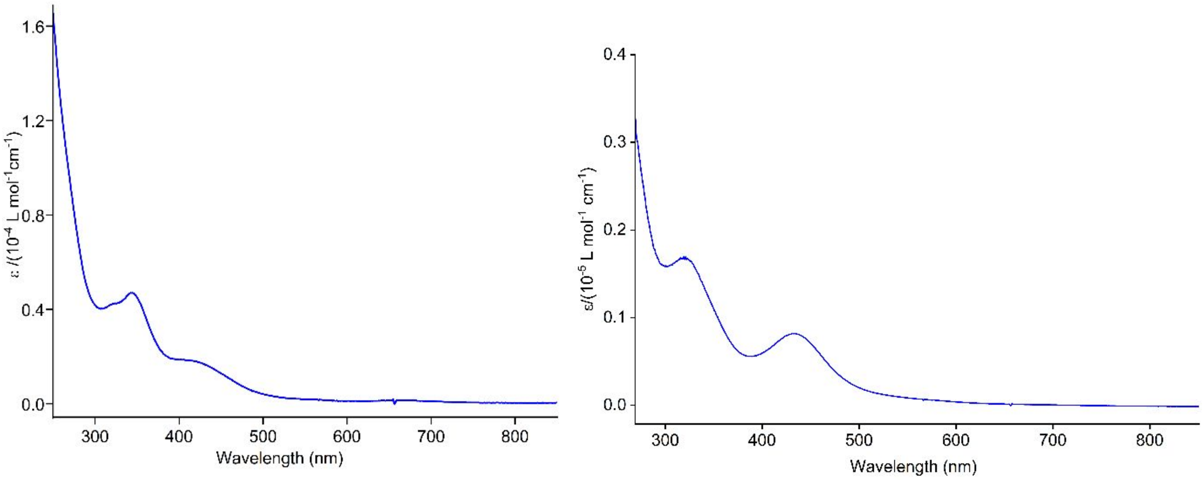

Figure S43. UV-Visible spectrum of ***(E)*-1a** (left) and ***(E)*-1b** (right) in diethyl ether.

B. Computational Details

DFT calculations were carried out with Orca 6.0 quantum chemical package.[6] Geometry optimizations were performed in the gas phase using the r^2SCAN-3c functional,[7] which combines the r^2SCAN meta-GGA functional with the D4 dispersion correction,[8] a geometrical counterpoise (gCP) correction,[9] and a short-range basis set correction (SRB). Vibrational frequency calculations were carried out at the same level of theory as the geometry optimizations (No imaginary frequency for local minima). Single-point calculations were carried out on the optimized geometries at the PBE0[10]-D4/X2C-TZVPPall[11] level of theory, including scalar relativistic effects via the exact two-component (X2C) Hamiltonian. Frontier molecular orbitals (HOMO and LUMO) were obtained from the Kohn–Sham orbital energies and visualized using Chemcraft.[12] Solvation effects of the experimentally used solvent (diethyl ether) were taken into account using the SMD[13] solvation model. Final electronic energies were computed via single-point calculations at the PW6B95-D4/ZORA[14]-def2-TZVP[15] level of theory (with SARC-ZORA-TZVP for the Bi atom) in combination with SMD (diethyl ether). Transition-state optimizations were located with NEB[16]/CI-NEB[17] method using the r^2SCAN-3c functional and the resulting transition-states were characterized by frequency calculations to confirm the presence of a single imaginary frequency corresponding to the desired reaction coordinate. Intrinsic reaction coordinate (IRC)[18] calculations were subsequently performed to ensure that each transition state connects the correct reactant and product minima. Natural bond orbital (NBO) calculations were carried out using NBO 7.0 program[19] at the PBE0-D4/def2-TZVP level of theory and visualized with Chemcraft.[12] Intrinsic bond orbital (IBOs)[20,21] computations were performed with ORCA program at the PBE0-D4/def2-TZVP level of theory and visualized by IBOview program.[20,21] Time-dependent density functional theory (TD-DFT) calculations for the UV–Vis absorption spectrum of E-1 were carried out using the PBE0 functional in conjunction with the ZORA-def2-TZVPP basis set (SARC-ZORA-TZVP for the Bi atom). Scalar relativistic effects were included via the ZORA Hamiltonian. Spin–orbit coupling (SOC)[22,23] effects were incorporated in the excited-state calculations by enabling SOC within the TD-DFT module (dosoc true) as implemented in the ORCA 6.0 quantum chemistry package. The SMD method was used with the corresponding solvent in the calculation TD-DFT, while Bondi radii[24] were chosen as the atomic radii to define the molecular cavity. The simulated result was visualized using Chemcraft.[12]

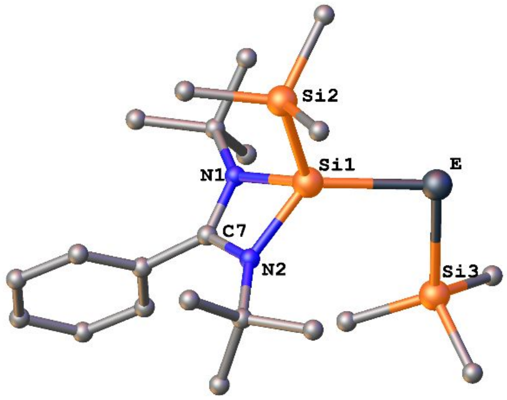

Bonds(Å) and angles (°)	Calculated [Experimental]					
	E=N	E=P	E=As	E=Sb	E=Bi	$LSi(SiMe_3)=BiSi(iPr)_3$
Si1-E	1.608	2.108	2.213	2.441	2.534 [2.536(1)]	2.534 [2.543(1)]
E-Si3	1.692	2.242	2.349	2.576	2.667 [2.637(1)]	2.683 [2.664(1)]
Si1-Si2	2.351	2.359	2.359	2.363	2.365 [2.351(1)]	2.366 [2.357(1)]
Si1-N1	1.880	1.881	1.880	1.881	1.882 [1.851(3)]	1.881 [1.855(4)]
Si1-N2	1.883	1.883	1.882	1.876	1.876 [1.850(3)]	1.876 [1.852(4)]
C7-N1	1.336	1.336	1.336	1.334	1.335 [1.337(5)]	1.341 [1.340(6)]
C7-N2	1.338	1.339	1.341	1.344	1.344 [1.338(5)]	1.337 [1.337(6)]
∠Si1-E-Si3	139.93	104.84	102.91	99.76	97.87 [97.83(3)]	108.80 [108.77(4)]
∠Si2-Si2-E	116.51	110.33	110.07	112.07	112.52 [112.48(4)]	108.20 [109.67(5)]
∠N1-Si1-N2	69.67	69.55	69.59	69.70	69.66 [71.00(11)]	69.64 [70.6(2)]

Figure S44. Optimized geometries of the complexes ($LSi(SiMe_3)=ESiMe_3$) (E = N, P, As, Sb, Bi) and $LSi(SiMe_3)=BiSi(iPr)_3$ at the r^{2}SCN-3c level. Experimental values are given in brackets for comparison. Selected bond lengths in Å, angles in °. Hydrogen atoms are omitted for clarity.

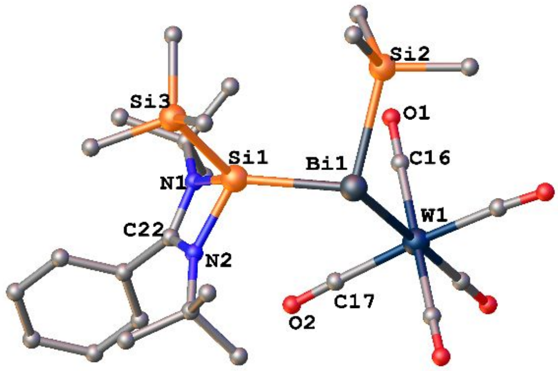

Bond lengths (Å) and angles (°)	Calculated [Experimental]	
	**(*Z*)-2a**	**(*Z*)-2b**
Si1-Bi1	2.624 [2.617(1)]	2.648 [2.63(1)/2.64(1)/]
Bi1-Si2	2.701 [2.669(2)]	2.714 [2.58(2)/2.79(2)]
Bi1-W1	3.003 [2.9559(3)]	3.012 [3.089(5)/2.817(5)]
Si1-Si3	2.381 [2.367(2)]	2.391 [2.31(2)/2.52(2)]
Si1-N1	1.848 [1.836(5)]	1.859 [1.87(2)/1.80(2)]
Si1-N2	1.853 [1.830(4)]	1.862 [1.59(4)/2.05(4)]
∠Si1-Bi1-Si2	98.03 [99.30(4)]	104.95 [99.30(4)/102.8(6)]
∠Si2-Bi1-W1	106.85 [107.68(3)]	105.12 [108.5(7)/102.8(6)]
∠Si1-Bi1-W1	107.01 [108.93(3)]	108.09 [108.8(6)/111.6(5)]
∠Si3-Si1-Bi1	118.77 [118.40(7)]	124.72 [118.2(9)/134(1)]
∠N1-Si1-N2	70.79 [71.4(2)]	70.39 [72(2)/68(1)]

Figure S45. Optimized geometries of (LSi($SiMe_3$)=Bi($SiMe_3$)W$(CO)_5$) and (LSi($SiMe_3$)=Bi(Si(iPr)$_3$)W$(CO)_5$) at the r^{2}SCN-3c level with Orca 6.0. Experimental values are given in brackets for comparison. Selected bond lengths in Å, angles in °. Hydrogen atoms are omitted for clarity.

a)

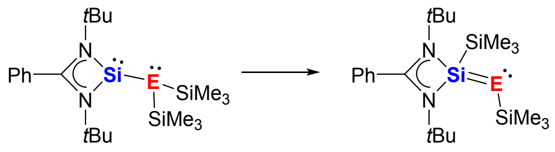

b)

**E**	Relative Gibbs free energy (kcal/mol)	Reaction energy barrier (kcal/mol)
N	7.3	31.1
P	-3.9	30.1
As	-5.9	27.8
Sb	-7.5	27.9
Bi	-9.5	26.0

c)

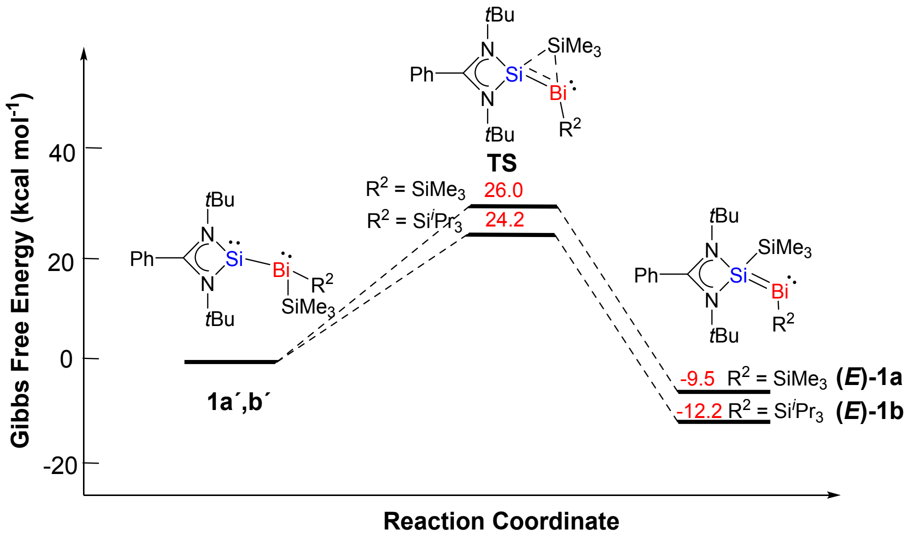

Figure S46. a) Hypothetical conversion of amidinato silylene-E complexes to Si=E (E = N, P, As, Sb, Bi) compounds. b) Relative Gibbs free energy of the corresponding reaction energy barriers in Kcal/mol calculated at the PW6B95-D4/SARC-ZORA-TZVP (for Bi and Sb) and ZORA-def2-TZVP (for other elements) level of theory with Orca 6.0. c) Depiction of relative Gibbs free energy and the reaction energy barrier in Kcal/mol for conversion of **1a´**,**b´** to ***(E)*-1a**,**b**, respectively.

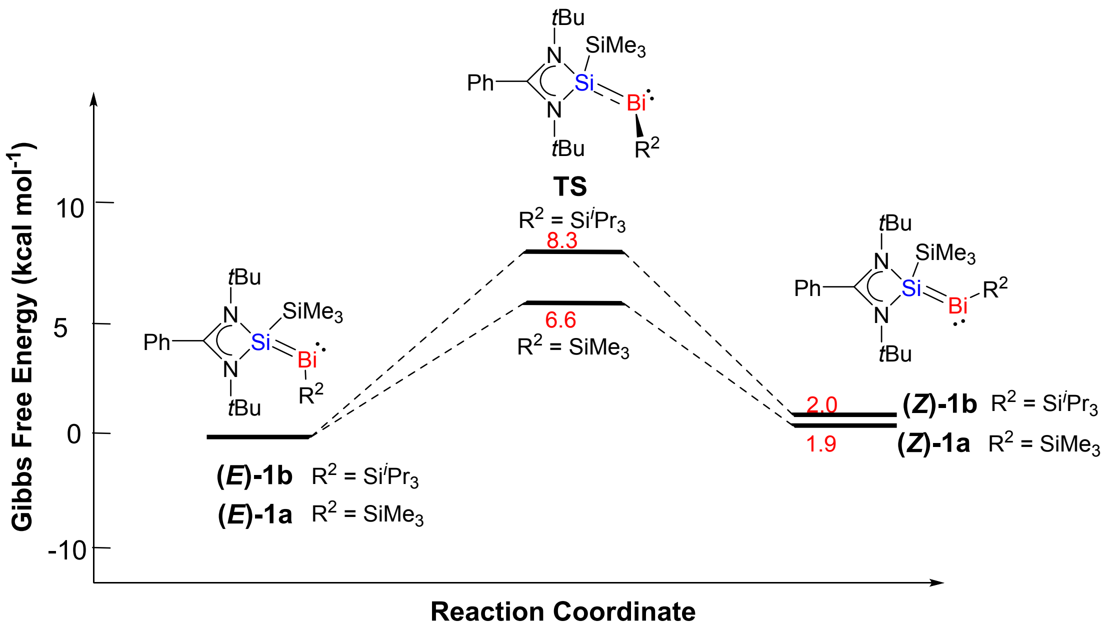

Figure S47. DFT-derived relative free energies and the reaction energy barrier for the interconversion of ***(E)/(Z)*** isomers of compound **(*E*)-1a.b** through Si=Bi p_π -bond rotation.

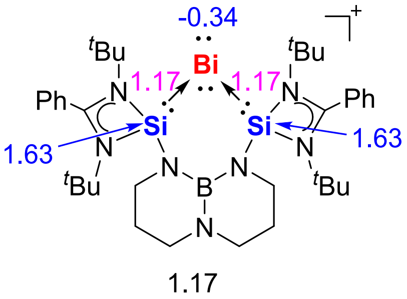

IV

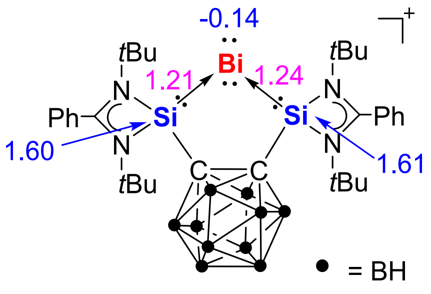

V

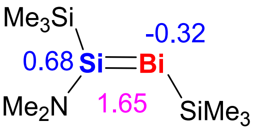

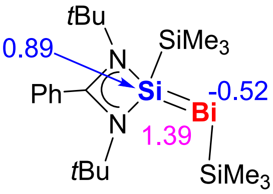

(*E*)-1a

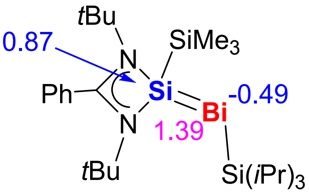

(*E*)-1b

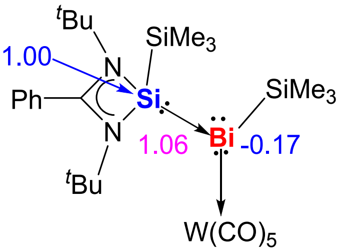

(*Z*)-2a

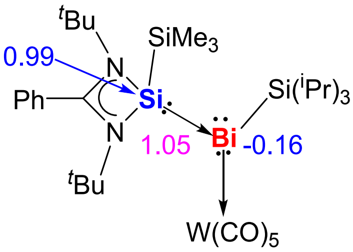

(*Z*)-2b

Figure S48. Wiberg index (pink) of the Si-Bi bonds and the NPA charges (blue) of the central Si and Bi atoms calculated by NBO at the PBE0-D4/def2-TZVP level of theory for **IV** and **V**, the hypothetic ***(E)***-silylidenbismane, **(*E*)-1a**,**b** and **(*Z*)-2a**,**b**.

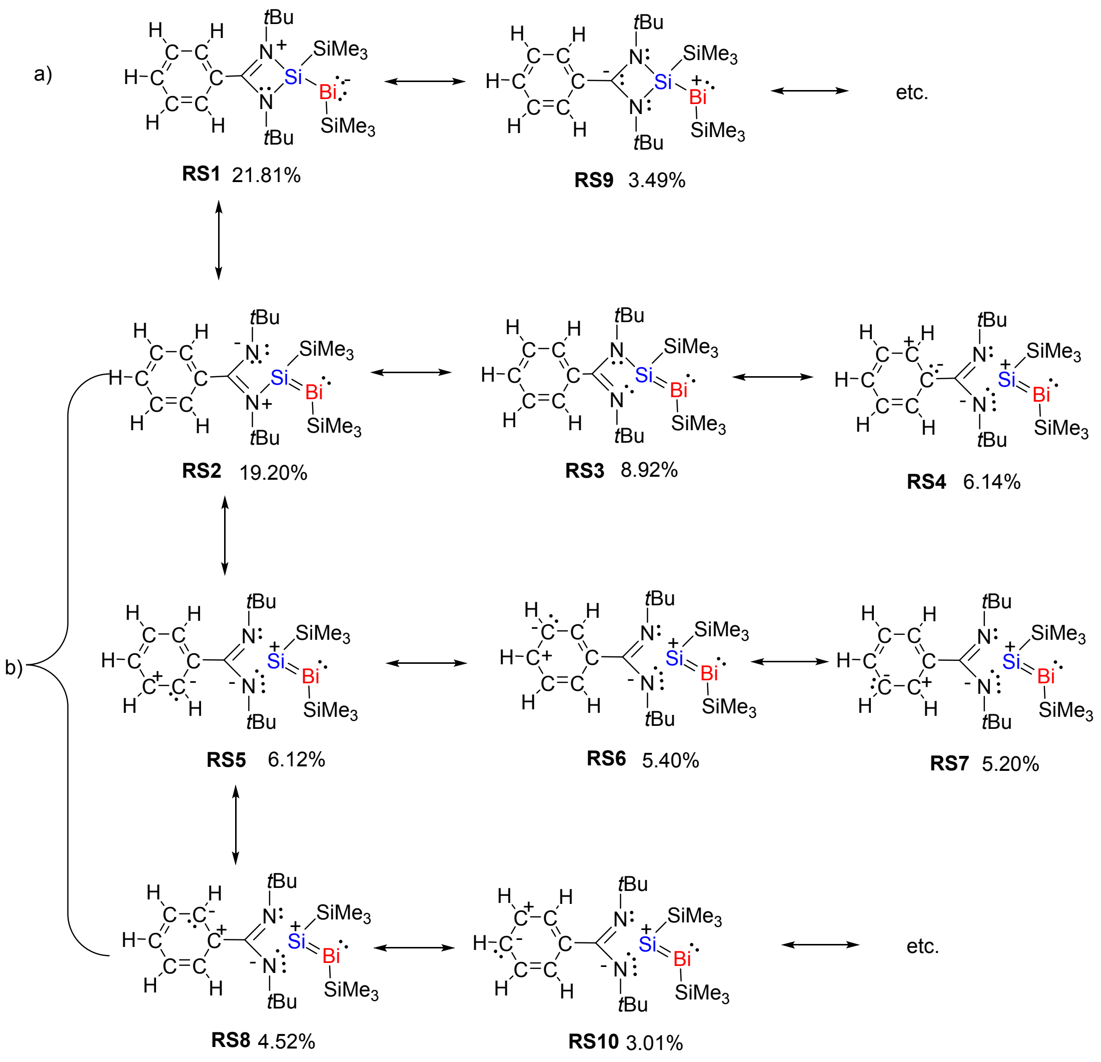

Scheme S7. Selected predominant resonance structures **RS1-10** of compound ***(E)*-1a** with resonance weight larger than 3% calculated by NBO(NRT) at the PBE0-D4/def2-TZVP level of theory: a) the resonance structures containing an Si-Bi single bond; b) those featuring an Si=Bi double bond. See more in the next Table.

Table S17. List of NRT resonance structures of **(*E*)-1a**.

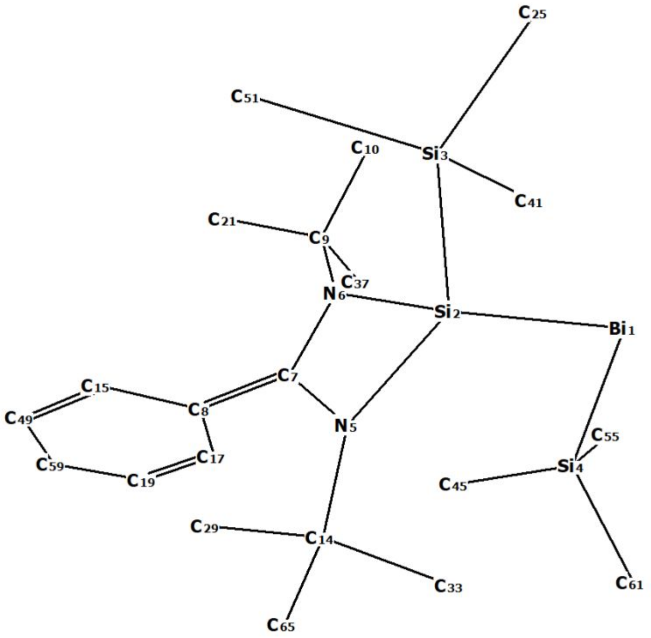

Numbering of the atoms in **(*E*)-1a**

RS 1 21.81%

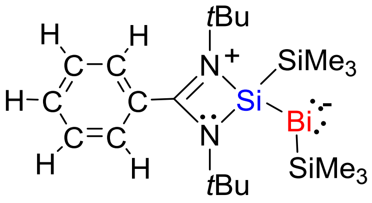

List of all Resonances

RS	Weight(%)	Added(Removed) based on **RS 1**
1	**21.81**	
2	19.20	**Bi 1-Si 2**, (Si 2- N 6), N 5- C 7, (N 6- C 7), (Bi 1), (N 5), N 6, N 6
3	8.92	**Bi 1-Si 2**, (Si 2- N 5), N 5- C 7, (N 6- C 7), (Bi 1), N 6
4	6.14	**Bi 1-Si 2**, (Si 2- N 5), (Si 2- N 6), (C 8- C 17), (C 15- C 49), C 17- C 19, (C 19- C 59), C 49- C 59, (Bi 1), N 5, N 6, C 8
5	6.12	**Bi 1-Si 2**, (Si 2- N 5), (Si 2- N 6), C 8- C 15, (C 8- C 17), (C 15- C 49), (C 19- C 59), C 49- C 59, (Bi 1), N 5, N 6, C 17
6	5.40	**Bi 1-Si 2**, (Si 2- N 5), (Si 2- N 6), C 8- C 15, (C 8- C 17), (C 15- C 49), C 17- C 19, (C 19- C 59), (Bi 1), N 5, N 6, C 49
7	5.20	**Bi 1-Si 2**, (Si 2- N 5), (Si 2- N 6), C 8- C 15, (C 8- C 17), (C 15- C 49), (C 19- C 59), C 49- C 59, (Bi 1), N 5, N 6, C 19
8	4.52	**Bi 1-Si 2**, (Si 2- N 5), (Si 2- N 6), (C 8- C 17), (C 15- C 49), C 17- C 19, (C 19- C 59), C 49- C 59, (Bi 1), N 5, N 6, C 15
9	**3.49**	(N 6- C 7), (Bi 1), N 6, C 7
10	3.01	**Bi 1-Si 2**, (Si 2- N 5), (Si 2- N 6), (C 7- C 8), C 8- C 15, (C 8- C 17),

		(C 15- C 49), C 17- C 19, (C 19- C 59), (Bi 1), N 5, N 6, C 7, C 59
11	1.70	**Bi 1-Si 2**, (Si 2- N 5), (Si 2- N 6), N 5- C 7, (N 6- C 7), (Bi 1), Si 2, N 6
12	1.65	**Bi 1-Si 2**, (Si 2- N 5), (Si 2- N 6), (C 7- C 8), C 8- C 15, (C 8- C 17), (C 15- C 49), C 17- C 19, (C 19- C 59), (Bi 1), N 5, N 6, C 7, C 8
13	1.35	**Bi 1-Si 2**, (Si 2-Si 3), (Si 2- N 5), (N 6- C 7), (C 8- C 17), C 17- C 19, (C 19- C 59), (Bi 1), N 5, N 6, C 7, C 59
14	1.26	**Bi 1-Si 2**, (Si 2- N 5), (Si 2- N 6), (C 7- C 8), C 8- C 15, (C 8- C 17), (C 15- C 49), C 17- C 19, (C 19- C 59), (Bi 1), N 5, N 6, C 7, C 8
15	1.16	**Bi 1-Si 2**, (Si 2- N 5), (Si 2- N 6), (C 8- C 17), (C 15- C 49), C 17- C 19, (C 19- H 20), (C 19- C 59), C 49- C 59, (Bi 1), N 5, N 6, C 8, C 15
16	1.14	**Bi 1-Si 2**, (Si 2- N 5), (Si 2- N 6), C 8- C 15, (C 8- C 17), (C 15- C 49), C 17- C 19, (C 19- C 59), (Bi 1), N 5, N 6, C 59
17	1.11	**Bi 1-Si 2**, (Si 2- N 5), (Si 2- N 6), (C 8- C 17), (C 15- C 49), C 17- C 19, (C 19- C 59), C 49- C 59, (C 59- H 60), (Bi 1), N 5, N 6, C 8, C 15
18	0.95	**Bi 1-Si 2**, (Si 2- N 5), (Si 2- N 6), C 8- C 15, (C 8- C 17), (C 15- H 16), (C 15- C 49), (C 19- C 59), C 49- C 59, (Bi 1), N 5, N 6, C 15, H 16
19	0.84	**Bi 1-Si 2**, (Si 2- N 5), (Si 2- N 6), C 8- C 15, (C 8- C 17), (C 15- H 16), (C 15- C 49), (C 19- C 59), C 49- C 59, (Bi 1), N 5, N 6, C 17, C 19
20	**0.78**	(Si 2- N 5), (N 6- C 7), (C 8- C 17), C 17- C 19, (C 19- C 59), (Bi 1), N 5, N 6, C 7, C 59
21	0.77	**Bi 1-Si 2**, (Si 2- N 5), (Si 2- N 6), C 8- C 15, (C 8- C 17), (C 15- C 49), (C 19- C 59), (C 49- H 50), C 49- C 59, (Bi 1), N 5, N 6, C 17, C 19
22	0.66	**Bi 1-Si 2**, (Si 2- N 5), (Si 2- N 6), N 5- C 7, (N 6- C 7), (Bi 1), Si 2, N6
23	0.57	**Bi 1-Si 2**, (Si 2-Si 3), (Si 2- N 5), (N 6- C 7), C 8- C 15, (C 8- C 17), (C 15- C 49), (Bi 1), N 5, N 6, C 7, C 17
24	0.56	**Bi 1-Si 2**, (Si 2- N 5), (Si 2- N 6), (C 8- C 17), (C 15- C 49), C 17- C 19, (C 19- H 20), (C 19- C 59), C 49- C 59, (Bi 1), N 5, N 6, C 19, H 20
25	0.55	**Bi 1-Si 2**, (Si 2- N 5), (Si 2- N 6), (C 8- C 17), (C 15- C 49), C 17- C 19, (C 19- C 59), C 49- C 59, (C 59- H 60), (Bi 1), N 5, N 6, C 59, H 60
26	0.51	**Bi 1-Si 2**, (Si 2-Si 3), (Si 2- N 5), (N 6- C 7), (C 15- C 49), (C 19- C 59), C 49- C 59, (Bi 1), N 5, N 6, C 7, C 15
27	0.48	**Bi 1-Si 2**, Bi 1-Si 2, (Bi 1-Si 4), (Si 2-Si 3), (Si 2- N 6), (Bi 1), Si 3, N 6
28	0.13	**Bi 1-Si 2**, (Si 2- N 5), (Si 2- N 6), C 8- C 15, (C 8- C 17), (C 15- C 49), (C 17- H 18), C 17- C 19, (C 19- C 59), (Bi 1), N 5, N 6, C 17, H 18
others	0.01	
	100.00	* Total *

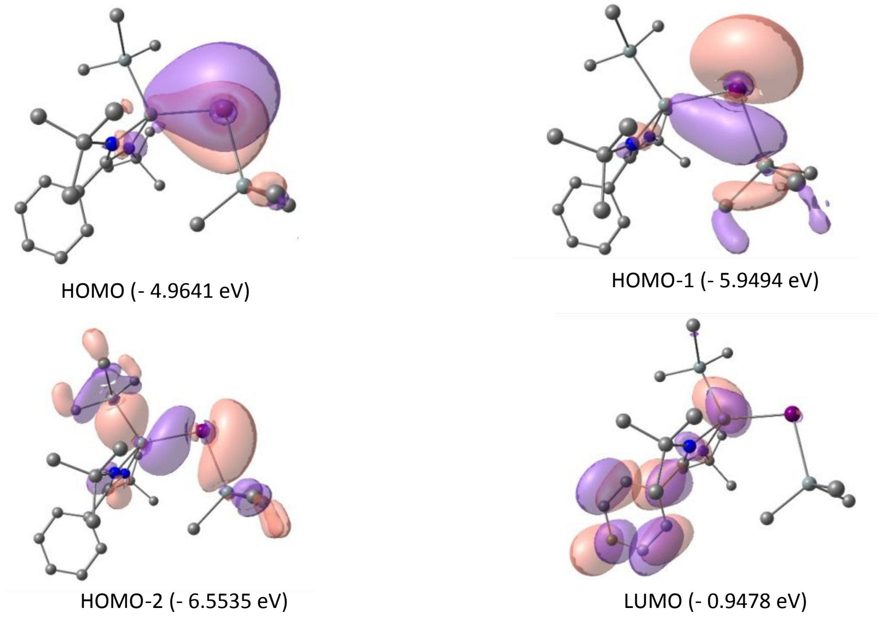

Figure S49. The plot of selected HOMOs and LUMO of ***(E)*-1a** at PBE0-D4/X2C-TZVPPall level.

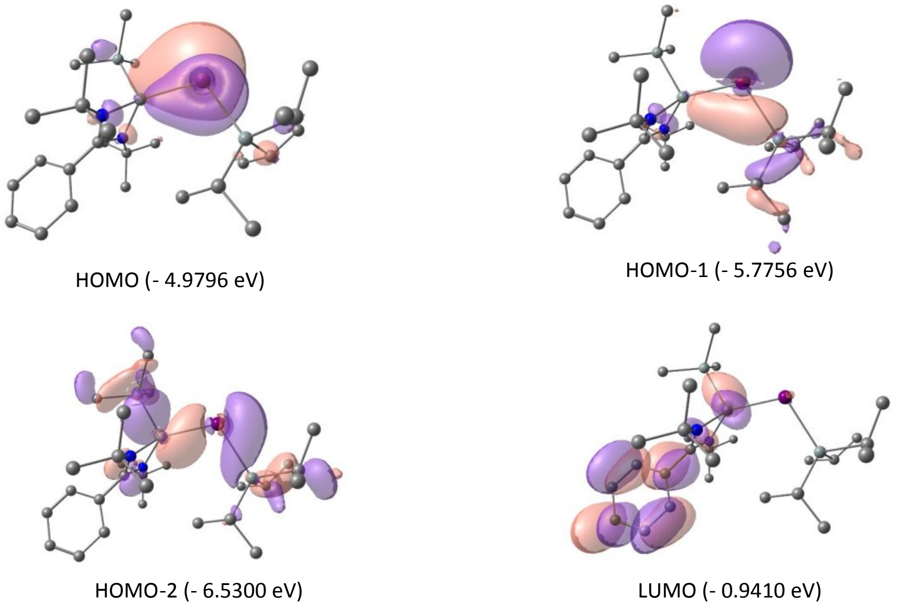

Figure S50. The plot of selected HOMOs and LUMO of ***(E)*-1b** at PBE0-D4/X2C-TZVPPall level of theory.

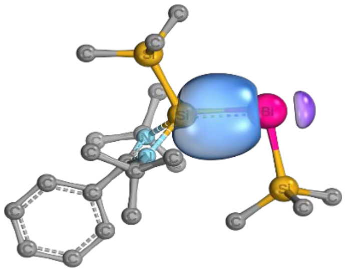

a) Si-Bi σ-bonding orbital.

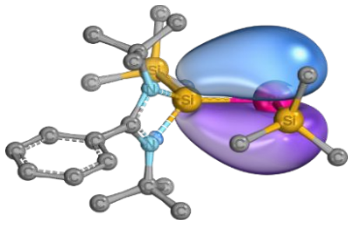

b) Si-Bi π-bonding orbital.

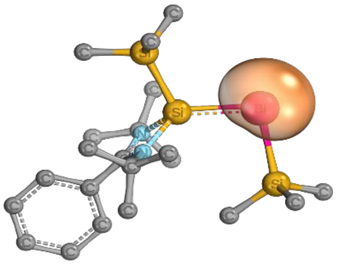

c) Bi lone pair orbital

Figure S51. Depiction of selected IBOs (a)-(c) of compound ***(E)*-1a** at the PBE0-D4/def2-TZVP level of theory.

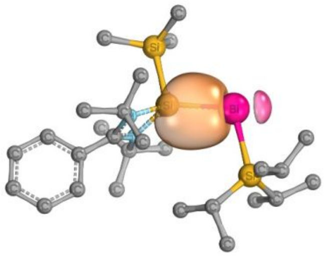

a) Si-Bi σ-bonding orbital.

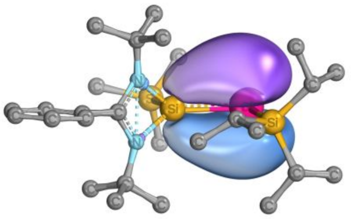

b) Si-Bi π-bonding orbital.

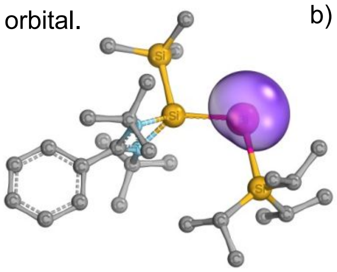

c) Bi lone pair orbital.

Figure S52. Depiction of selected IBOs (a)-(c) of compound ***(E)*-1b** at the PBE0-D4/def2-TZVP level of theory.

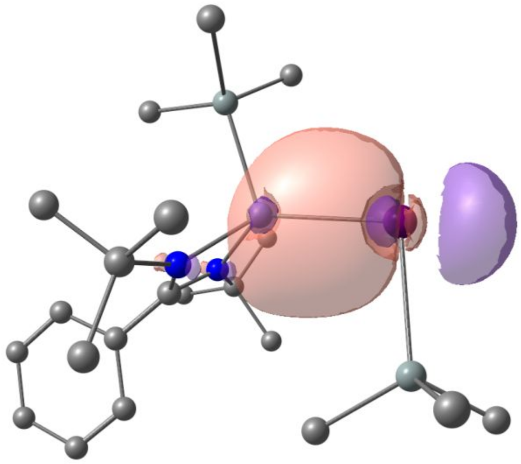

(a) Si-Bi σ-bonding orbital (BD (1)Si(56.25%,sp$^{1.21}$) – Bi(43.75%,sp$^{10.42}$), occ = 1.95 eL).

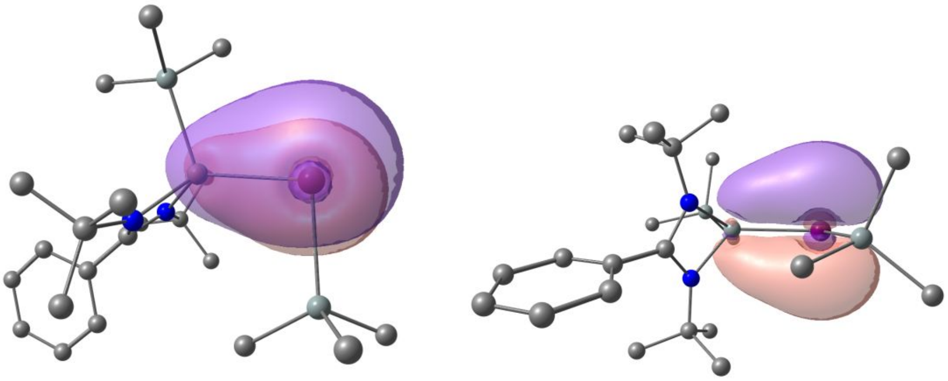

(b) Different views of the Si-Bi π-bonding orbital

(BD (2) Si(16.68%, p orbital) – Bi(83.32%, p orbital), occ = 1.93 eL).

Figure S53. Depiction of the NBOs of the Si=Bi bond in compound ***(E)*-1a** at PBE0-D4/def2-TZVP level of theory.

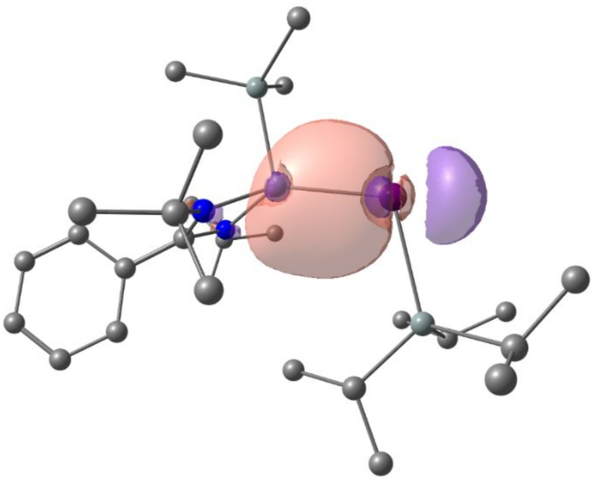

(a) Si-Bi σ-bonding orbital (BD (1)Si(56.87%,$sp^{1.14}$) – Bi(43.13%,$sp^{10.80}$), occ = 1.95 eL).

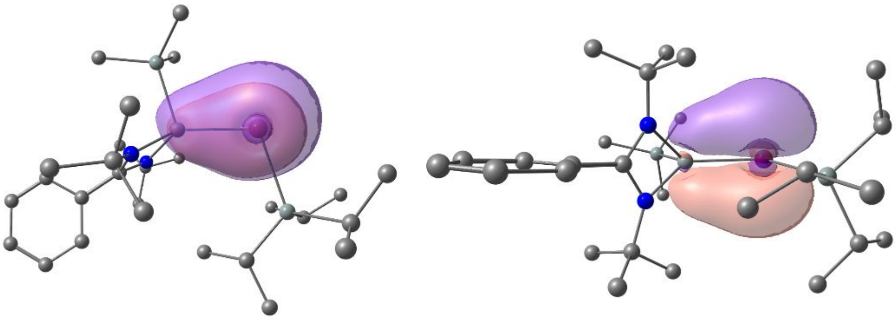

(b) Different views of the Si-Bi π-bonding orbital

(BD (2) Si(16.80%, p orbital) – Bi(83.20%, p orbital), occ = 1.91 eL).

Figure S54. Depiction of the NBOs of the Si=Bi bond in compound ***(E)*-1b** at PBE0-D4/def2-TZVP level of theory.

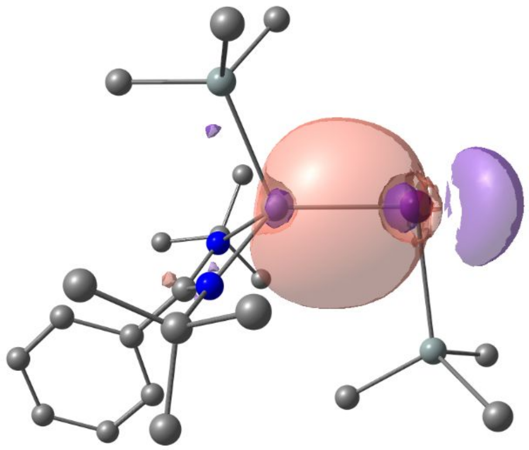

a) Si-Bi σ-bonding orbital (BD (1)Si(55.18%, $sp^{0.95}$) – Bi(42.68%,$sp^{9.61}$), occ = 2 eL).

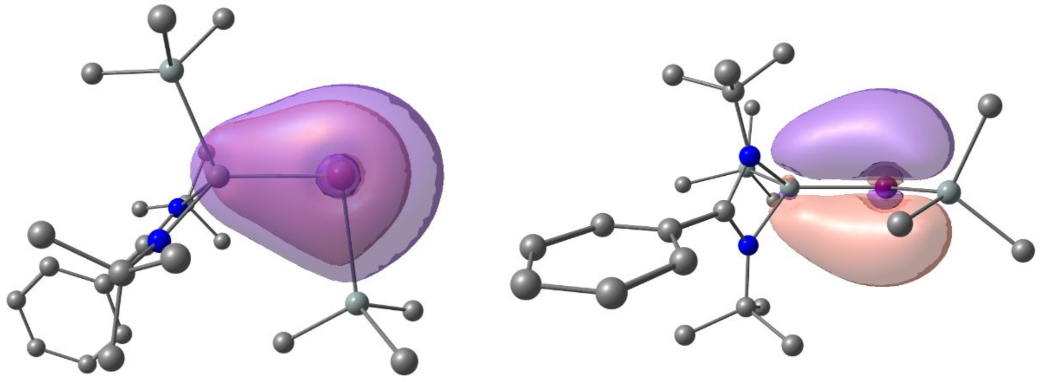

b) Different views of the Si-Bi π-bonding orbital (BD (2)Si(15.98%,p orbital) – Bi(80.32%,p orbital), occ = 2.eL).

Figure S55. Depiction of the selected NLMO (Natural Localized Molecular Orbital) of compound ***(E)*-1a** at PBE0-D4/def2-TZVP level of theory.

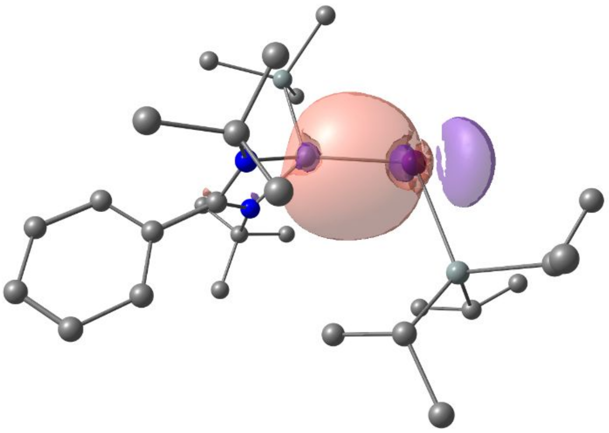

a) Si-Bi σ-bonding orbital (BD (1)Si(55.80%, $sp^{0.91}$) – Bi(42.13%,$sp^{9.68}$), occ = 2 eL).

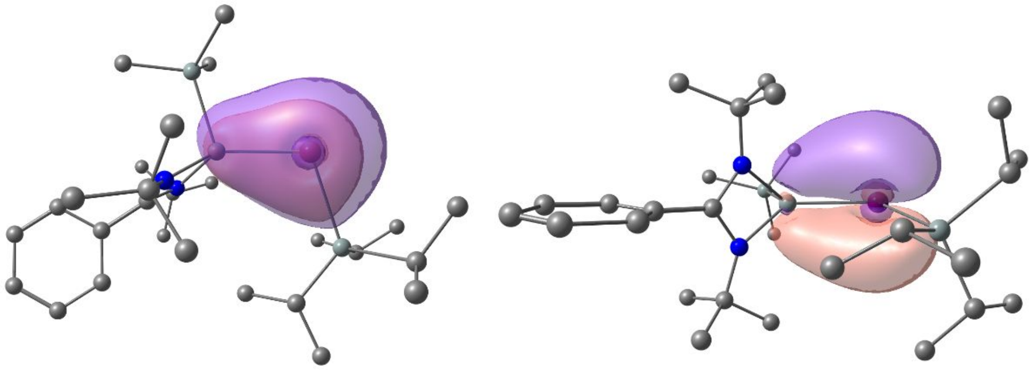

b) Different views of the Si-Bi π-bonding orbital

(BD (2)Si(15.95%,p orbital) – Bi(79.71%,p orbital), occ = 2.eL).

Figure S56. Depiction of the selected NLMO (Natural Localized Molecular Orbital) of compound ***(E)*-1b** at PBE0-D4/def2-TZVP level of theory.

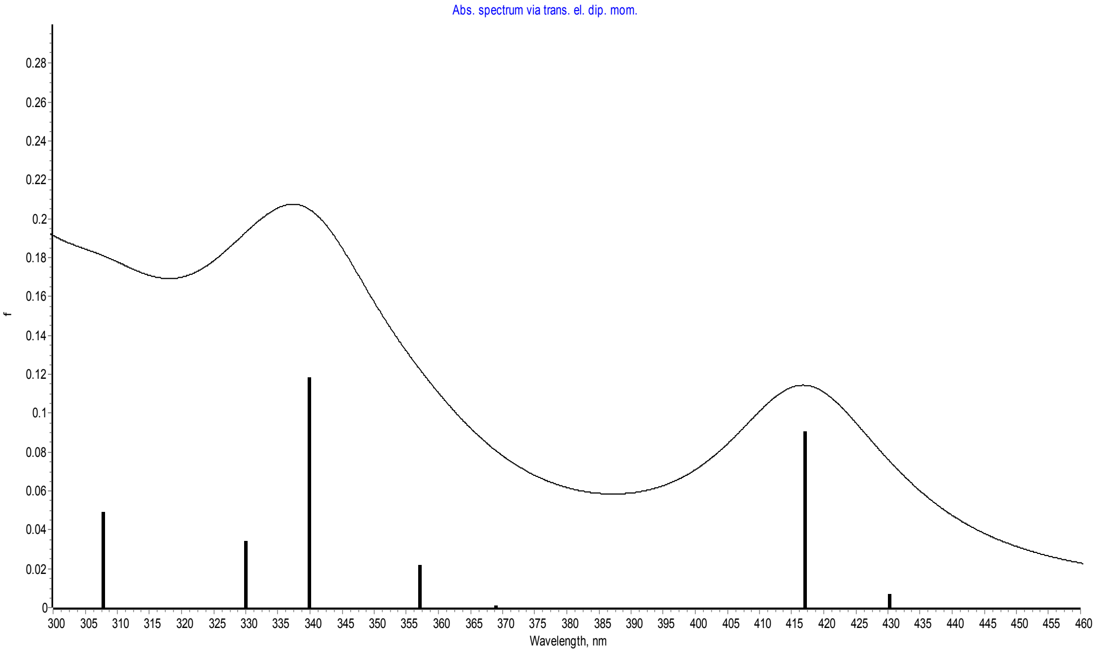

(a)

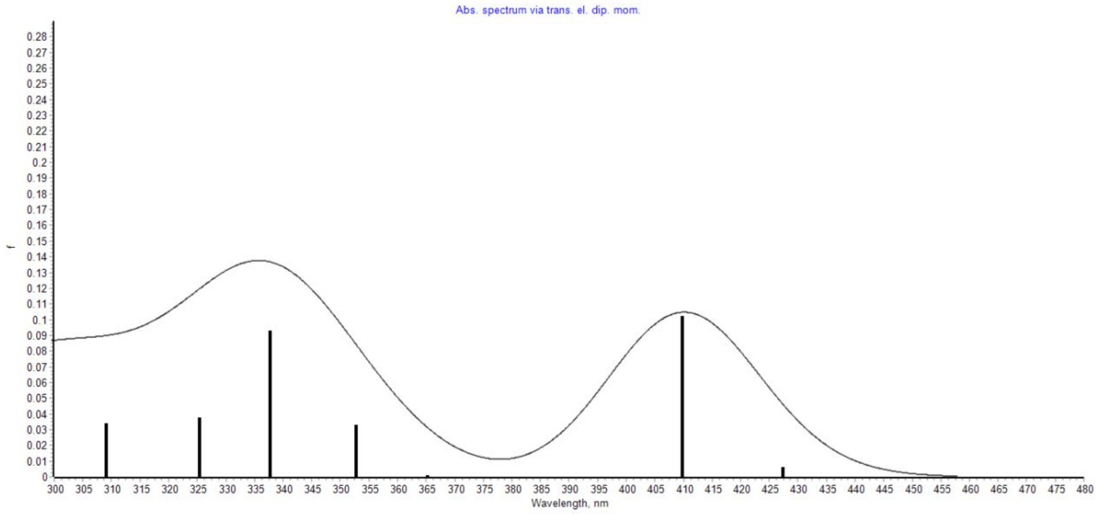

(b)

Figure S57. Simulated UV–vis spectra of **(*E*)-1a** (a) and **(*E*)-1b** (b) obtained from TD-DFT calculations at the PBE0/ZORA-def2-TZVPP level of theory (SARC-ZORA-TZVP for the Bi atom), including relativistic effects via the ZORA Hamiltonian and spin–orbit coupling. Spin–orbit coupling leads to strong mixing of the excited-state manifold, resulting in SOC-admixed states composed primarily of several close-lying TD-DFT roots. The low-energy absorption band (~420–450 nm) originates predominantly from SOC-mixed states derived mainly from the lowest excited roots, while the higher-energy band (~330–350 nm) arises from higher-lying mixed states. Orbital contributions of the parent TD-DFT states indicate that these transitions are mainly associated with HOMO→LUMO-derived and higher excited configurations.

C. References

(1) Sen, S. S.; Roesky, H. W.; Stern, D.; Henn, J.; Stalke, D. High Yield Access to Silylene RSiCl (R = PhC(N *t* Bu)$_2$) and Its Reactivity toward Alkyne: Synthesis of Stable Disilacyclobutene. *J. Am. Chem. Soc.* **2010**, *132* (3), 1123–1126.

(2) Sheldrick, G. Crystal structure refinement with SHELXL. *Acta Crystallogr. C* **2015**, 71, 3−8.

(3) Bourhis, L. J.; Dolomanov, O. V.; Gildea, R. J.; Howard, J. A. K.; Puschmann, H. The many faces of Olex2. *J. Appl. Crystallogr.* **2015**, A71, 59−75

(4) Dolomanov, O. V.; Bourhis, L. J.; Gildea, R. J.; Howard, J. A. K.; Puschmann, H. OLEX2: A Complete Structure Solution, Refinement and Analysis Program. *J. Appl. Crystallogr.* **2009**, *42* (2), 339–341.

(5) Rookes, T. M.; Wildman, E. P.; Balázs, G.; Gardner, B. M.; Wooles, A. J.; Gregson, M.; Tuna, F.; Scheer, M.; Liddle, S. T. Actinide−Pnictide (An−Pn) Bonds Spanning Non-Metal, Metalloid, and Metal Combinations (An=U, Th; Pn=P, As, Sb, Bi). *Angew. Chem., Int. Ed.* **2018**, *57*, 1332−1336

(6) Neese, F. Software Update: The ORCA Program System—Version 6.0. *WIREs Comput. Mol. Sci* **2025**, *15* (2), e70019.

(7) Grimme, S. Hansen, A. Ehlert, S. Mewes, J. -M. *J. Chem. Phys.* **2021**, *154(6)*, 064103.

(8) Caldeweyher, E.; Ehlert, S.; Hansen, A.; Neugebauer, H.; Spicher, S.; Bannwarth, C.; Grimme, S. A Generally Applicable Atomic-Charge Dependent London Dispersion Correction. *J. Chem. Phys.* **2019**, *150* (15), 154122.

(9) Brandenburg, J. G.; Grimme, S. Accurate Modeling of Organic Molecular Crystals by Dispersion-Corrected Density Functional Tight Binding (DFTB). *J. Phys. Chem. Lett.* **2014**, *5* (11), 1785–1789.

(10) Adamo, C.; Barone, V. Toward Reliable Density Functional Methods without Adjustable Parameters: The PBE0 Model. *J. Chem. Phys.* **1999**, *110* (13), 6158–6170.

(11) a) Kutzelnigg, W. Liu, W. Quasirelativistic theory equivalent to fully relativistic theory. *J. Chem. Phys.* ***2005,*** *123*, 241102; b) Pollak, P. Weigend, F. Segmented contracted error-consistent basis sets of double- and triple- ζ valence quality for one- and two-component relativistic all-electron calculations. *J. Chem. Theory Comput.* **2017***, 13*, 3696–3705; c) Franzke, Y. J. Treß, R. Pazdera, T. M. Weigend, F. Error-consistent segmented contracted all-electron relativistic basis sets of double- and triple-zeta quality for NMR shielding constants. *Phys. Chem. Chem. Phys.* **2019***, 21*, 16658–16664.

(12) Chemcraft - Graphical Software for Visualization of Quantum Chemistry Computations. https://www.chemcraftprog.com.

(13) Marenich, A. V.; Cramer, C. J.; Truhlar, D. G. Universal Solvation Model Based on Solute Electron Density and on a Continuum Model of the Solvent Defined by the Bulk Dielectric Constant and Atomic Surface Tensions. *J. Phys. Chem. B* **2009**, *113* (18), 6378–6396.

(14) van Lenthe, E. Snijders, J. G. Baerends, E. J. The zero-order regular approximation for relativistic effects: The effect of spin–orbit coupling in closed shell molecules. *J. Chem. Phys.***1996***, 105*, 6505–6516.

(15) Weigend, F.; Ahlrichs, R. Balanced Basis Sets of Split Valence, Triple Zeta Valence and Quadruple Zeta Valence Quality for H to Rn: Design and Assessment of Accuracy. *Phys. Chem. Chem. Phys.* **2005**, *7* (18), 3297.

(16) Mills, G.; Jónsson, H.; Schenter, G. K. Reversible Work Transition State Theory: Application to Dissociative Adsorption of Hydrogen. *Surf. Sci.* **1995**, *324* (2–3), 305–337.
(17) Henkelman, G.; Uberuaga, B. P.; Jónsson, H. A Climbing Image Nudged Elastic Band Method for Finding Saddle Points and Minimum Energy Paths. *J. Chem. Phys.* **2000**, *113* (22), 9901–9904.
(18) Fukui, K. The Path of Chemical Reactions - the IRC Approach. *Acc. Chem. Res.* **1981** *14*, 363–368.
(19) Glendening, E. D.; Badenhoop, J. K.; Reed, A. E.; Carpenter, J. E.; Bohmann, J. A.; Marales, C. M.; Karafiloglou, P.; Landis, C. R.; Weinhold, F. NBO 7.0, 2018.

(20) Knizia, G. Intrinsic Atomic Orbitals: An Unbiased Bridge between Quantum Theory and Chemical Concepts. *J. Chem. Theory Comput.* **2013**, *9* (11), 4834–4843.
(21) Knizia, G.; Klein, J. E. M. N. Electron Flow in Reaction Mechanisms—Revealed from First Principles. *Angew. Chem., Int. Ed.* **2015**, *54* (18), 5518–5522.
(22) Heß, B. A. Marian, C. M. Wahlgren, U. Gropen, O. A mean-field spin-orbit method applicable to correlated wavefunctions. *Chem. Phys. Lett.***1996***, 251*, 365–371.

(23) Neese, F. Efficient and accurate approximations to the molecular spin-orbit coupling operator and their use in molecular *g*-tensor calculations. *J. Chem. Phys.* **2005***, 122*, 34107.

(24) Bondi, A. Van Der Waals Volumes and Radii. *J. Phys. Chem.* **1964**, *68* (3), 441–451.
